# Food and Agricultural Approaches to Reducing Malnutrition (FAARM): protocol for a cluster-randomised controlled trial to evaluate the impact of a Homestead Food Production programme on undernutrition in rural Bangladesh

**DOI:** 10.1136/bmjopen-2019-031037

**Published:** 2019-07-04

**Authors:** Amanda S Wendt, Thalia M Sparling, Jillian L Waid, Anna A Mueller, Sabine Gabrysch

**Affiliations:** 1 Heidelberg Institute of Global Health, Heidelberg University, Heidelberg, Germany; 2 Friedman School of Nutrition Science and Policy, Tufts University, Boston, Massachusetts, USA; 3 Bangladesh Country Office, Helen Keller International, Dhaka, Bangladesh; 4 Research Department 2, Potsdam Institute for Climate Impact Research, Potsdam, Germany; 5 Institute of Public Health, Charité - Universitätsmedizin Berlin, Berlin, Germany

**Keywords:** nutrition, community child health, public health, epidemiology

## Abstract

**Introduction:**

Chronic undernutrition affects over 150 million children worldwide and has serious consequences. The causes are complex and include insufficient dietary diversity and poor hygiene practices. Systematic reviews of nutrition-sensitive agricultural interventions concluded that while these hold promise, there is insufficient evidence for their impact on child growth. The Food and Agricultural Approaches to Reducing Malnutrition (FAARM) project is a 1:1 cluster-randomised trial aiming to evaluate the impact of a Homestead Food Production (HFP) programme implemented by Helen Keller International on women’s and children’s undernutrition.

**Methods and analysis:**

The HFP intervention comprises training of women’s groups and asset distribution to support year-round home gardening, poultry rearing and improved nutrition and hygiene practices. Formal trainings are supplemented by behaviour change communication during household visits, and facilitated links between producer groups and market actors. The FAARM trial will examine if and how this complex intervention reduces undernutrition. In 2015, FAARM enrolled married women and their children (0–3 years) in 96 rural settlements of Habiganj district in Sylhet division, Bangladesh. Covariate-constrained randomisation was used to assign 48 settlements to receive a 3-year HFP intervention, with the other 48 acting as controls, targeting over 2700 women. To study impact pathways, a surveillance system collects data on all participants every 2 months. In late 2019, children 0–3 years of age (born during the intervention period) will be surveyed, thus capturing impact during the critical first 1000 days of life. Children’s length/height-for-age z-scores will be compared between intervention and control arms using mixed-effects linear regression. Secondary outcomes include women’s and children’s micronutrient status, dietary intake, dietary diversity and other indicators of child growth, development and morbidity.

**Ethics and dissemination:**

Ethical approval was received in Bangladesh and Germany. Results will be disseminated through peer-reviewed publications and presentations in Bangladesh and internationally.

**Trial registration number:**

NCT02505711; Pre-results.

**Strengths and limitations of this study**

- The trial includes 96 clusters and used covariate-constrained randomisation, thus ensuring good baseline balance.
- We evaluate programme impact on children conceived after the start of the intervention who will have benefited during their first 1000 days of life.
- A wide range of knowledge, behavioural, anthropometric and biological indicators will be assessed in women and children.
- Rigorous monitoring and surveillance systems provide a platform to examine outputs and outcomes along the causal pathway.
- We measure child growth and blood parameters at baseline in early 2015 and at endline in late 2019, but not continuously over the course of the trial.

## Introduction

Child undernutrition is responsible for an estimated 45% of child mortality, about 3.1 million deaths annually.^1^ Suboptimal nutrition during the first 1000 days, from conception to 2 years of age, can have irreversible consequences for child development and long-term health, intelligence, educational attainment and work capacity.^1 2^

To reduce undernutrition sustainably, one must address its complex root causes. The two main direct causes according to the Unicef and Lancet frameworks are nutrient-deficient diets and frequent gastrointestinal infections.^1 3^ An inadequate diet of the mother limits the nutrients the child receives during pregnancy and from breast feeding, and an inadequate diet of the young child further contributes to undernutrition.^1^ Nutrient-deficient diets of women and children are often due to insufficient household and maternal knowledge and resources, while unhygienic living conditions favour gastrointestinal infections.^1 3^ In addition to the short-term negative influence of infections on appetite and nutrient absorption, frequent subclinical gastrointestinal infections may cause chronic inflammation and damage to the mucosal barrier in the gut and disrupt the intestinal microbiome, leading to ‘environmental enteric dysfunction’, further inhibiting the absorption of nutrients.^4^

Due to the many complex causes of undernutrition, programmes that address several underlying factors of child nutrition and health are needed. However, the evidence on ‘potentially promising and culturally relevant’ interventions to diversify diets by ‘enhancement of agricultural production’ has been weak, because most such interventions ‘have only been implemented at a small scale, and have not been adequately assessed’, according to the Lancet’s 2008 undernutrition series.^5^ Evaluations of nutrition-sensitive agricultural interventions, that is, agricultural interventions focusing not only on yields, but on improving nutrition of vulnerable groups, have been relatively rare, leading to a gap in our understanding of their potential contribution to reducing child undernutrition.^6^

Previous evaluations of nutrition-sensitive agriculture programmes found that these can improve household access and consumption of targeted foods and in some cases improve household, maternal or child dietary diversity, however, the evidence of their impact on child anthropometric outcomes was very weak, according to several reviews in 2012–2013.^6–8^ This lack of evidence was largely attributed due to methodological shortcomings of previous studies, such as lack of appropriate control groups and small sample sizes.^6–8^

In 2018, Ruel *et al* evaluated the recent literature (since 2014) and found several studies which had improved methodologies and examined a wide range of nutrition-sensitive agricultural interventions, including eight Homestead Food Production (HFP) programmes.^9^ Ruel *et al*, and another recent review report that most studies showed increases in targeted food production, household and child dietary diversity, and in micronutrient intake when measured. Some also showed impacts on anaemia or micronutrient status. However, evidence on stunting was still lacking. Possible reasons cited were short programme durations, poor age targeting (outside the critical 1000 days) and insufficient statistical power to detect changes in stunting prevalence.^9 10^

With the Food and Agricultural Approaches to Reducing Malnutrition (FAARM) trial, we attempt to address these limitations through a strong cluster-randomised design, sufficient sample size and duration, recruitment strategies to target children in their first 1000 days, as well as measuring a wide range of indicators along the causal pathway.

### Study aims and objectives

The study will evaluate the impact of an HFP programme implemented by Helen Keller International (HKI) on length/height-for-age z-score among children aged 0–3 years who were conceived during the intervention period.

### Methods and analysis

#### Study design

The FAARM trial is a 1:1 cluster-randomised controlled trial with repeated cross-sectional surveys of children born to a cohort of women. To cover the first 1000 days of life, we enrolled young, married women into the HFP programme in 2015, with the target group at endline in 2019 being children under 3 years of age conceived by these women during the intervention period.

#### Study setting

The FAARM trial is conducted in Bangladesh where the prevalence of food insecurity and undernutrition is high. In 2014, over half of women consumed inadequately diverse diets, almost a fifth had a body mass index (BMI) below 18.5 kg/m^2^, and 35% of children under 5 years were stunted.^11^ Child marriage and early childbearing are also common in the country: almost half of women had their first pregnancy by age 18.^11^ Only 20% of pregnant women receive adequate antenatal care and about 40% of mothers practise exclusive breast feeding during the first 6 months.^11^ Complementary feeding practices for children aged 6–23 months are poor with only 43% receiving an adequately diverse diet.^11^ In addition, women in Bangladesh and their families struggle with persistent poverty, gender inequality and lack of women’s agency, climate change and political volatility.

Within Bangladesh, the study area was identified through an analysis of population demographics, including proxies for wealth, results from national nutrition surveys and information about other interventions in the area. Sylhet division, in North-eastern Bangladesh, has a prevalence of undernutrition above the country average, with 44% of children under five stunted and 40% of pregnant women undernourished in 2014.^11^ Sylhet division also had the largest wealth disparity and the highest fertility in Bangladesh, and performed worst on a range of gender indicators.^12–15^

#### Sample size

The study sample size was calculated in relation to the primary outcome, length/height-for-age. A continuous z-score was used, that is, length/height in SD below or above the mean for a child of a given age and sex, which can then be translated into the percentage of children ‘moderately or severely stunted’ (below −2 SD). We aimed to detect a minimum difference of 0.20–0.25 in mean z-score at a 5% significance level. This effect size has been seen in studies using educational approaches to improve child growth.^16^

The sample size for each study arm was first calculated for a simple randomised design and then multiplied by a design factor to account for the clustered design as there is greater similarity of children’s nutritional status within a cluster. The intracluster correlation of length-for-age z-score was estimated to be 0.03, and for a minimum detectable effect size of 0.22 with 90% power, sample size was calculated as 677 children per arm, assuming a cluster size of 15. To account for loss to follow-up, possibly due to late refusal, withdrawal from the study or permanent migration out of the study area, an extra 10% was added. Therefore, we aimed for approximately 745 children per arm, 1490 children in total. Assuming an average of 15 children per cluster, born to 28 women, this meant that nearly 100 clusters needed to be randomised.

#### Participant recruitment and cluster formation

**Figure 1:**
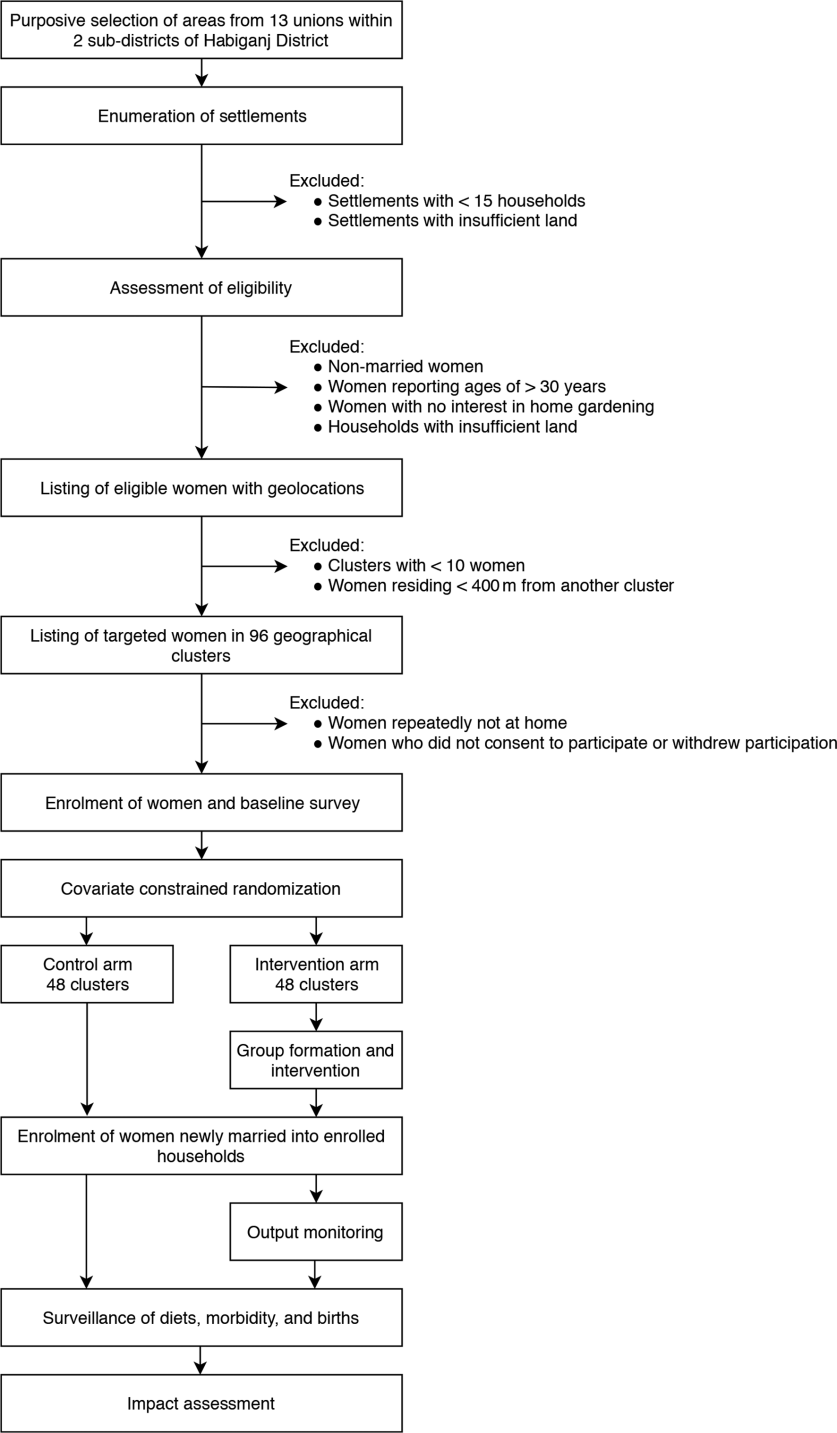
Flow chart of FAARM participant selection, randomisation, monitoring and evaluation. FAARM, Food and Agricultural Approaches to Reducing Malnutrition.

Once the study site in Baniachong and Nabiganj subdistricts of Habiganj district, Sylhet division, Bangladesh had been selected, we enumerated all households in the area to identify women eligible to participate in FAARM. A woman was eligible for inclusion if she reported being 30 years old or less, was married and her husband stayed overnight at the household at least once in the year prior to interview, had access to at least 40 square metres of land (ideally 10 square metres near the house) and was interested to participate in an HFP programme (figure 1).

**Figure 2:**
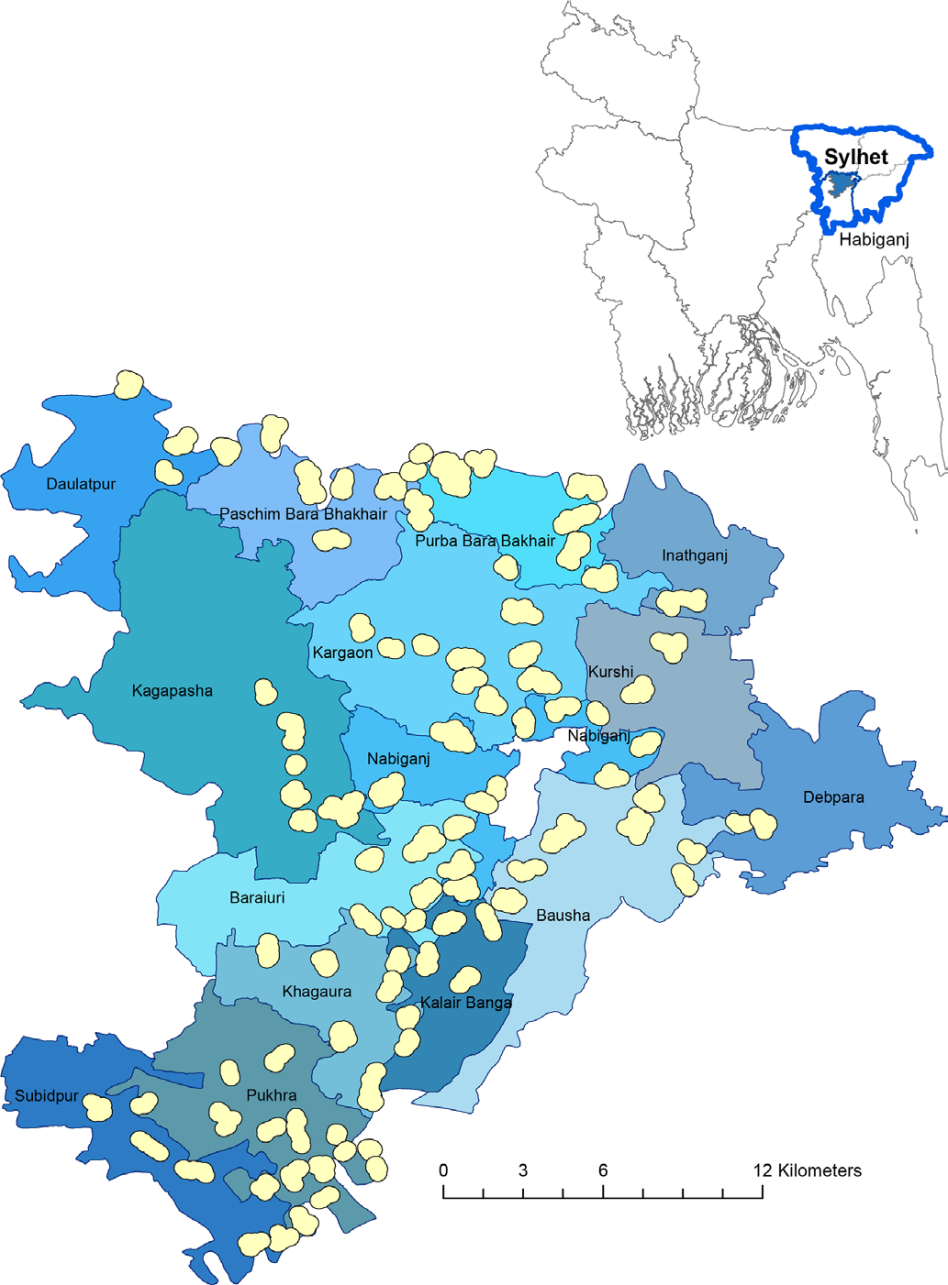
FAARM study site. The large map shows the FAARM study site with 96 clusters in 13 unions of 2 subdistricts (Nabiganj and Baniachong) and the small map on the top shows its location within Habiganj district and Sylhet division in Bangladesh. FAARM, Food and Agricultural Approaches to Reducing Malnutrition.

Once eligible women were identified, clusters (the unit of randomisation) were formed with at least 10 and no more than 65 eligible women, based on geographical location of women’s residences. A minimum distance of 400 m between settlements was maintained to limit contamination. Figure 2 shows the study site within Bangladesh, and the 96 clusters within 13 unions of Nabiganj and Baniachong subdistricts.

**Figure 3:**
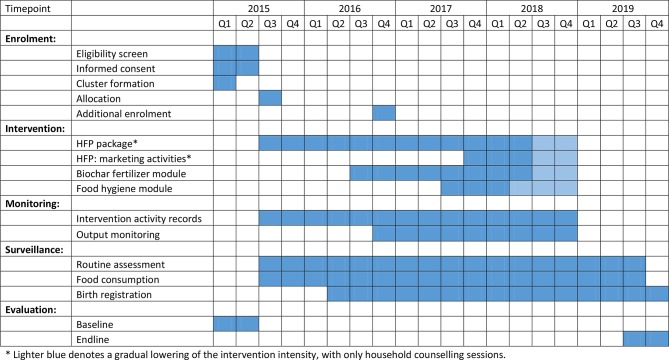
Activity timeline for FAARM trial in Sylhet, Bangladesh. FAARM, Food and Agricultural Approaches to Reducing Malnutrition; HFP, Homestead Food Production.

At the start of the baseline survey, eligible women who had been assigned to a cluster were approached for consent (figure 3). Some women were not enrolled in the trial because they had migrated since enumeration, were repeatedly not at home at the time of the survey visits or refused to participate. With limited birth records, and official birth records frequently incorrect, many adults cannot accurately report their age. During the baseline survey, age was again ascertained using event calendars to get more accurate estimates. In cases where the newly estimated age at baseline was over 30 years, we did not exclude these women from the trial. The youngest biological child of an enrolled woman was assessed at baseline if the child was born after 1 March 2012, 3 years prior to the first day of the baseline survey training.

During the first year of implementation, we additionally invited women (and their youngest biological children) to enrol who had married into households already participating in the trial (figure 1). The number of women enrolled in this way was nearly identical in the intervention and control groups.

#### Cluster randomisation

After the baseline survey, 96 clusters were randomly assigned to the intervention or control group (figure 3). We used covariate-constrained randomisation to ensure good balance, that is, that important baseline characteristics did not differ greatly between arms,^17^ using a self-programmed Stata routine.^18^ The randomisation was constrained by covariates on the cluster, household, woman and child level. (Cluster: union and number of women; Household: religion, food consumption score, household food insecurity access score, log of agricultural land size; Women: height, BMI, haemoglobin; Child: age, age-adjusted z-scores of length-for-age and weight-for-length, haemoglobin, diarrhoea prevalence.)

#### The intervention

HKI has been implementing HFP programmes in Bangladesh since 1988, working in partnership with over 70 local non-governmental organisations (NGOs).^19^ The objectives of the HFP programmes are to increase household access and consumption of nutrient-dense foods through gardening, poultry rearing and nutrition counselling, and to empower women through increased control of assets and through group participation and leadership. Together these changes are thought to improve the health and nutrition of women and children. Over time, HKI has adapted the programme based on community needs and evolving evidence and expanded to several countries in Africa and Asia.^20–22^

Similar to earlier HFP projects, FAARM’s HFP programme is implemented jointly with a local partner NGO, the Voluntary Association for Rural Development (VARD). Three full-time technical staff from HKI manage the local project office and provide support and direction to eight full-time project staff, a project coordinator and an accountant from VARD. Each project staff member is responsible for a certain geographical region for which they have monthly targets for trainings, individual counselling and asset distribution.

Prior to programme activities, 64 households with existing home gardens were visited in six villages in the project area, interviewed on previous experiences and all food crop species were identified by agricultural researchers. Most gardens had few species and plants and were only active few months per year. Key issues reported were pests, poor soil fertility, excessive shade and flooding exposure (preventing year-round gardening). Strategies to overcome these were incorporated into the HFP curriculum.

Programme activities begin with organising project participants into village-level ‘women farmer groups’ of around 16 households each (ranging from 8 to 26). This is the key intervention unit. Within these groups, members select a lead farmer family, who oversees a village model farm on their property and acts as a multiplier. The lead family receives additional training and is encouraged to implement all recommended improved techniques, produce seeds and seedlings for distribution to other participants, and host trainings and meetings. In larger clusters where one village model farm may provide for several groups, additional women serve as group leaders. At project closeout, at least one motivated participant in each group will be selected as a peer educator to remind neighbours of promoted activities after the intervention has concluded.

FAARM’s HFP intervention includes a series of trainings, usually held at the village model farm. All participants will receive trainings on year-round vegetable gardening, fruit tree production, poultry rearing and the implications of household production and consumption on health. In the first year, 1-day trainings are given on winter and summer crop cultivation, seed production and preservation, and poultry rearing, while half-day refresher trainings are given in subsequent years. The trainings emphasise sustainable gardening techniques, including the use of raised beds, live fencing, integrated pest management, composting and soil management, and seed preservation. In the third year of the intervention, once homestead production and consumption are established and there is surplus production, marketing training is given to build linkages with local vendors in order to provide opportunities for income generation for women. In response to issues previously identified in the area, additional trainings are given on sack gardening (during the rainy season when gardens are prone to flooding) and on production and application of urine-enriched biochar fertiliser (to enhance soil fertility), implemented as part of a separate study.^23^

Furthermore, project staff conduct courtyard sessions every 2 months for participants and family members, covering nutrition, health and hygiene topics. An adult learning approach is used, which includes dialogue, story-telling, role play and drawing as well as joint activities such as food preparation. Session content follows the global Essential Nutrition Actions approach, including optimal breast feeding, infant and young child feeding and nutrition for women.^24^

In addition to formal trainings and meetings, project staff visit all participants individually in their households every 2 months to review nutrition, health and hygiene messages as well as provide technical support for gardening and poultry rearing activities. These interpersonal counselling sessions provide opportunities for participants to bring up issues they are facing that may be difficult to discuss in a group setting, and for project staff to tailor messages to the unique situation of the participant and her family.

In the third year of the intervention, an additional food hygiene training is added to reinforce messages on dietary diversity and cover food-related hygiene topics more intensively, in particular hand-washing, clean utensils, food storage and reheating (see online supplementary file 1). This intervention is delivered by eight female staff and consists of eight structured events, four group events and four household visits.

10.1136/bmjopen-2019-031037.supp1 Supplementary data

FAARM’s HFP intervention also transfers assets. Seeds are distributed to all project households twice a year to coincide with the main planting seasons and include nutrient-rich, seasonally and locally appropriate vegetables such as red amaranth, water spinach, tomato, pumpkin, string bean and cabbage. Similarly, tree saplings, chosen for nutrient-rich fruits such as papaya, lemon and jujube, were transferred to households with greater land availability. Participants also receive poultry assets, primarily a reimbursement for the majority of the cost of a constructed, improved poultry shed, as well as starter feed and watering stations.

A gender-equitable approach is integrated into the HFP structure with the programme aiming to increase women’s status in the household through asset transfer and control of productive resources as well as the skill transfer associated with HFP trainings. Improving women’s status and agency is thought to further support programme activities and improve child nutrition.^25^

#### Programme theory

**Figure 4:**
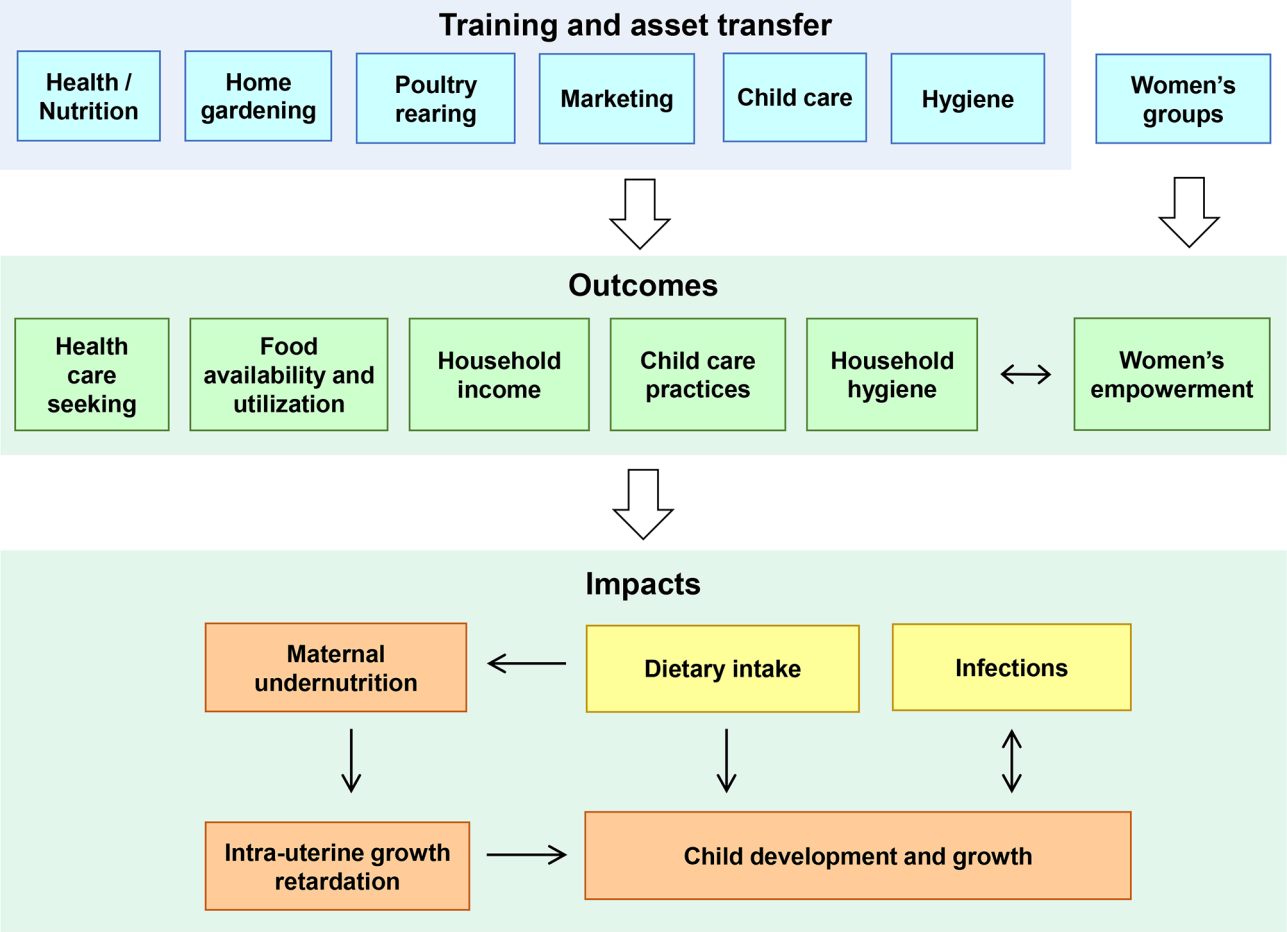
FAARM conceptual framework. The blue boxes represent components of Homestead Food Production intervention trainings and counselling sessions. These will influence the outcomes listed (the green boxes), with women’s empowerment interacting with all aspects. Better outcomes then lead to improved impacts including child development and growth. FAARM, Food and Agricultural Approaches to Reducing Malnutrition.

A conceptual framework (figure 4) was constructed to illustrate the HFP programme’s theory of change. Training on gardening and poultry rearing can increase household access to nutrient-dense foods, counselling on health, hygiene and child care topics may lead to improved practices, and the marketing of surplus products can increase household income. Throughout the programme, the focus on women’s participation and leadership may increase women’s empowerment by increasing their decision-making role and social support. When these outcomes are achieved, we expect to see improvements in dietary intake and reduced infections, leading to reduced maternal and child undernutrition and better child development.

#### Control arm

In both intervention and control settlements, there are certain benefits delivered alongside the data collection activities. These include free pregnancy tests, birth counselling to establish a birth plan, and a visit within 3 days of birth where staff weigh and measure the child and provide breast feeding counselling to the mother.

#### Monitoring plan

FAARM’s monitoring plan consists of three major elements: intervention activity records, output monitoring of intervention households and a multipart surveillance system that includes both intervention and control households (figure 3). Indicators measured through these systems are outlined in a detailed project monitoring plan based on identified project impact pathways. All systems gather data using Open Data Kit (ODK) Collect software on Android mobile devices.

##### Intervention activity records

Project monitoring forms were designed to track programme contact points, including attendance of each participant and her family members at courtyard sessions and trainings, and tracking when and what topics were included in counselling sessions. In addition, supervisors document their monitoring of project staff activities.

##### Output monitoring

Project staff conduct monitoring interviews of FAARM intervention participants on a rolling basis, with each respondent visited once per year (table 1). In these interviews, outputs and outcomes, such as garden diversity and yields, poultry production and knowledge gained from training modules, are assessed in the intervention arm.

**Table 1:** Food and Agricultural Approaches to Reducing Malnutrition data sources and data collection methods

	Data source	Data collection device	Responsible team	Population	Frequency
Survey	Baseline	Tablet	Survey team	Intervention and control group	Once in 2015
Programme monitoring	Intervention activity records	Paper, then smartphone	Implementation team	Intervention group	Rolling: during or after programme activities
Programme monitoring	Output monitoring	Smartphone	Implementation team	Intervention group	Annually on a rolling basis
Surveillance	Routine assessment	Tablet	Surveillance team	Intervention and control group	Every 2 months
Surveillance	Food consumption	Tablet	Surveillance team	Subsample: intervention and control group	Annually on a rolling basis
Surveillance	Birth registry	Tablet	Surveillance team	At birth: intervention and control group	Within 72 hours of childbirth
Survey	Endline	Tablet	Survey team	Intervention and control group	Once in 2019

##### Surveillance

FAARM’s surveillance system in intervention and control households contains three components explained in detail below: (1) routine assessment, (2) food consumption assessment and (3) birth registration (figure 3). It serves to examine indicators on the two main pathways, diet and infection, through which the intervention could impact child undernutrition, as well as assessing maternal undernutrition and intrauterine growth retardation (figure 4). All surveillance activities are conducted by locally recruited, female data collectors. Extensive training with field practice is provided before any data collector begins work. Before each 2-month data collection period, a refresher training of 1–2 days is held to review and troubleshoot as well as train on any questionnaire aspects that have been added or changed. Participants are identified for interviews using project ID cards or by verifying the names of the participant, husband and household head. Collected data are reviewed regularly for real-time monitoring and analysis. The surveillance system is summarised in table 1, while information on indicators collected is available in online supplementary table 1.

10.1136/bmjopen-2019-031037.supp2 Supplementary data

A routine assessment round is completed every 2 months. Women report their last menstrual periods using provided calendars to mark the day. Pregnancies are identified with the help of pregnancy tests, which are provided for free to women for voluntary self-administration. Pregnant women are asked about antenatal care, and their weight and mid-upper arm circumference are measured at every visit, while non-pregnant women are weighed once per year. Dietary diversity information is recorded from all pregnant women each round and from non-pregnant women every 6 months, using 21 food groups.^26^ The Edinburgh Perinatal/Postnatal Depression Scale is administered during pregnancy, shortly after delivery, and after the child has attained 6 months of age.^27 28^ For all children who are below 3 years of age (at the first day of each 2-month round), we ask mothers about recent diarrhoea and acute respiratory infection episodes. Mothers of children 0–18 months are asked all questions from the standard WHO Infant and Young Child Feeding survey module each round, while children above 18 months are asked these questions every 6 months.^29^ Starting in the second year of the trial, the six WHO motor milestones are measured on all children as appropriate for their age.^30^ Starting in the third year of the trial, all children have their weight recorded around 18 months of age. Every 4 months, women are asked about the different crop species grown in their gardens in the previous season (cool-dry, hot-dry, rainy) to assess diversity of production. Annually, questions are asked about dietary habits during Ramadan. Periodically, additional questions are added, for example, about poultry production, garden characteristics, household hygiene and food security.

Detailed quantitative food consumption information is recorded through dietary recall on a randomly selected subset of nine households per cluster, that is, a total of 864 households. To achieve even distribution over seasons and arms, the 96 clusters were divided into two phases of 48 clusters, with 24 intervention and 24 control clusters in each phase. Data are collected every 2 months, that is, six times per year, alternating between phases, with each cluster thus being visited three times per year to interview three households each time. Total food consumed by the household and how this was distributed among all household members is reported by the primary food preparer. A separate module about snacks and food intake between meals is asked to all available household members separately. In a third of the sample each round (one of the three households per cluster), diets are recorded on two non-consecutive days, less than 10 days apart, in order to enable estimation of usual intake and the proportion of the population at risk of inadequate intake. The methods used in data collection are derived from those designed by the International Food Policy Research Institute for the Bangladesh Integrated Household Survey.^31^ This procedure is repeated annually in the same households in the same season.

Birth registration activities are initiated on all identified pregnancies in the third trimester. Pregnant mothers are provided with free phone cards and encouraged to notify the team when they have delivered. An automated calling list is used by birth registration team members to give reminder calls to the pregnant women before their estimated date of birth. As soon as notified, ideally within 72 hours of birth, a trained female data collector visits the family, measures the newborn’s weight, length and head circumference, interviews the mother on the delivery, and provides initial breast feeding counselling. Birth registration data collectors are trained on standard anthropometric measurement techniques for newborns and calibrate measurement equipment on each day of use.

### Evaluation plan

#### Trial outcomes and measurements

##### Primary outcome

The primary outcome measure is length/height-for-age z-score in children under 3 years of age at endline, using the 2006 WHO child growth standards.^32^

##### Secondary outcomes

Secondary outcome measures include other measures of child growth, namely weight-for-length/height and head circumference-for-age z-scores in children 0–3 years; and length/height-for-age z-score in children 6–30 months, as well as growth outcomes at birth (length-for-gestational-age, weight-for-gestational-age and head circumference-for-gestational-age). For women’s nutritional status, indicators are BMI in non-pregnant women, and mid-upper arm circumference and gestational weight gain in pregnant women. Micronutrient status is measured through anaemia (haemoglobin), vitamin A deficiency (retinol-binding protein), iron deficiency (serum ferritin, soluble transferrin receptor) and zinc deficiency (serum zinc) in women and children, adjusting for inflammation (serum C-reactive protein, serum α−1-acid glycoprotein) when recommended (online supplementary table 2 and file 2).

Dietary diversity is measured according to published recommendations, using 10 food groups for women and 7 food groups for children.^26 29^ In addition, dietary adequacy is assessed for a subsample of women and children using the mean probability of adequate intake for 11 micronutrients (thiamine, riboflavin, niacin, vitamin B_6_, folate, vitamin B_12_, vitamin C, vitamin A, calcium, iron, zinc). Indicators for infections in children comprise 7-day period and 2-day point prevalences of diarrhoea and of acute respiratory infection. Early child development is assessed using WHO motor milestones.^30 33^

Food hygiene indicators include a hygiene behaviour score measured through structured observation in mothers with children 6–18 months of age, faecal contamination (total coliforms and *Escherichia coli*) of complementary food samples and enteropathy biomarkers (faecal myeloperoxidase, faecal α-1-antitrypsin, faecal neopterin, serum C-reactive protein, serum α−1-acid glycoprotein). This is conducted through the ‘Food Hygiene to reduce Environmental Enteric Dysfunction’ study nested into the FAARM trial (see online supplementary file 1).

##### Additional outcomes

The FAARM trial provides an ideal platform on which to explore other research topics, such as linkages between food production and diets, women’s agency and mental health, dietary influences on arsenic methylation capacity, mycotoxin exposure, (epi-)genetic factors influencing innate immunity and micronutrient metabolism, as well as diversity and composition of the mother’s and the child’s gut microbiome through which the above effects may be modified. Several of these additional research components will be explored during the course of the trial, contingent on time and funding.

##### Data collection

All measurements and samples are taken in accordance with current standards and methodology, building on questionnaire tools from HKI and James P Grant School of Public Health at BRAC University, including from the Food Security and Nutritional Surveillance Project in Bangladesh.^11^ HKI employs experienced field research assistants who receive specific training and supervision on anthropometric measurement and interview techniques. All blood sampling is undertaken by clinically trained staff, familiar with the appropriate methods of extraction, storage and processing.

Most trial outcomes are measured during the endline survey in late 2019 (figure 3). A week-long training is held for all data collectors, with separate trainings conducted for questionnaire interviewers, anthropometry and blood sampling staff, following standard measurement procedures.^34–36^ HKI staff conduct trainings for interviewers, covering survey question content, question-asking techniques and cultural sensitivity, and trainings for the anthropometry team, including standardisation exercises with local volunteers. Staff from icddr,b are responsible for training and supervision of blood and stool sampling and processing. Supervisors and trainers conduct quality control in the field and refresher trainings are given when needed. Data are collected via tablets and uploaded after each interview or at the end of the day. Datasets are examined for quality and adjustments to the questionnaires or refresher trainings with the data collectors are done as needed.

#### Data management

Questionnaires have been programmed to prevent common data collection errors. Data are compiled on a regular basis, and data quality checks are conducted. Data concerns are immediately communicated to the data collectors or research team for resolution. All quantitative and qualitative data are stored on password-protected servers and computers. Only deidentified data are shared with collaborators for analysis.

#### Statistical analysis plan

The main analysis will be to compare the mean length/height-for-age z-score in children, aged 0–35 months at the start of the endline survey, between intervention and control arms. This will be done using mixed-effects linear regression, that is, modelling between-cluster variation as a random effect in a full probability model. This method will be statistically more efficient than analysing results at the cluster level and also allows for analysis of individual-level covariates.^37^ In order to reduce between-cluster variation and thus increase power and precision of the study, we will adjust for baseline values of the main outcomes of interest (eg, mean length-for-age at cluster level) as well as potentially other relevant predictors. Secondary and other outcomes that are normally distributed will be analysed in the same way. Logarithmic transformation will be used for continuous variables with a skewed distribution (eg, agricultural yields). Binary outcomes (eg, health service use) will be analysed with mixed effects logistic regression models. Missing data on outcome measurements, including loss to follow-up, will be compared between intervention and control arms with respect to baseline covariates to assess the potential for selection bias.

### Patients and public involvement

Participants were not involved in the study design or in the setting of research objectives or outcomes. Intervention implementation is adapted to participant concerns and constraints. Key stakeholders will be involved in dissemination of research findings.

### Dissemination

#### Consent

All enrolled FAARM participants provide informed, written consent by signature or thumbprint if illiterate, for themselves and for their children, if applicable. All participants receive an information sheet with contact information of the local FAARM manager for any questions or concerns.

#### Risks and benefits

The risks from participation (time expenditure over 4 years, anthropometric measurement and biological sampling) are minimal when compared with the potential knowledge gained from this study. There are some benefits for all participants, including referral for serious conditions when detected during surveys, early pregnancy identification (through distribution of pregnancy tests) and initial breast feeding counselling. Participants in the intervention arm additionally benefit from intervention activities in the form of assets, training and support to adopt promoted behaviours.

All participants are informed about their anthropometric measurements in cases of low BMI or child growth, and haemoglobin values when anaemic. The results of the trial will be disseminated to all research and implementation partners, published in peer-reviewed journals, and presented at national and international conferences and events. Programme and policy-related findings will be distributed through the HKI network and other agricultural and nutrition fora.

#### Project status

As of April 2019, participant enrolment, baseline survey and intervention activities have been completed. The surveillance activities are ongoing and the endline survey is planned for late 2019.

## Discussion

To our knowledge, this is the first randomised controlled trial to assess the impact of a complex, nutrition-sensitive agricultural programme on children’s nutritional status that enrols women prior to pregnancy, thus capturing the first 1000 days of child development. It is particularly relevant and well placed in rural Bangladesh where stunting and micronutrient deficiencies are common. We will be able to assess whether the intervention has impacted each outcome and how this occurs, due to a comprehensive monitoring and surveillance system through which we track programme fidelity and intensity, participation and outcomes along impact pathways. By assessing participant knowledge and behaviours as well as anthropometric and biological measures, we will be able to examine changes on a wide range of indicators. In addition, the rotating data collection pattern of the surveillance system will allow the project to map out changes in diets over seasons and between intervention and control households over time. Data on the sources of foods consumed by households will enable tracking of changes in food consumption habits, comparing garden production to market purchases. A limitation of our study is the lack of continuous child growth monitoring and blood sampling during surveillance, due to financial and logistic constraints. Data collection of children will also end at 3 years of age, which may miss growth or child development impacts which appear later.

We expect that the findings from this trial will contribute to national policy discussions regarding the contribution of complex nutrition-sensitive agricultural interventions to improving nutritional status and other outcomes. Furthermore, trial results may be generalisable to other settings where poverty, food insecurity and climate change affect nutrition and health.

10.1136/bmjopen-2019-031037.supp3 Supplementary data

10.1136/bmjopen-2019-031037.supp4 Supplementary data

## Supplementary Material

Reviewer comments

Author's manuscript

## References

[1] Black RE, Victora CG, Walker SP, et al Maternal and child undernutrition and overweight in low-income and middle-income countries. Lancet 2013;382:427–51. 10.1016/S0140-6736(13)60937-X 23746772

[2] Ruel M, Hoddinott J Investing in early childhood nutrition. IFPRI Policy Briefs. Washington DC: International Food Policy Research Institute, 2008.

[3] UNICEF. Strategy for improved nutrition of children and women in development countries. New York, USA: UNICEF, 1990.

[4] Harper KM, Mutasa M, Prendergast AJ, et al Environmental enteric dysfunction pathways and child stunting: A systematic review. PLoS Negl Trop Dis 2018;12:e0006205 10.1371/journal.pntd.0006205 29351288 PMC5792022

[5] Bhutta ZA, Ahmed T, Black RE, et al What works? Interventions for maternal and child undernutrition and survival. Lancet 2008;371:417–40. 10.1016/S0140-6736(07)61693-6 18206226

[6] Masset E, Haddad L, Cornelius A, et al Effectiveness of agricultural interventions that aim to improve nutritional status of children: systematic review. BMJ 2012;344:d8222 10.1136/bmj.d8222 22251864 PMC3259800

[7] Ruel MT, Alderman H Maternal and Child Nutrition Study Group. Nutrition-sensitive interventions and programmes: how can they help to accelerate progress in improving maternal and child nutrition? Lancet 2013;382:536–51. 10.1016/S0140-6736(13)60843-0 23746780

[8] Girard AW, Self JL, McAuliffe C, et al The effects of household food production strategies on the health and nutrition outcomes of women and young children: a systematic review. Paediatr Perinat Epidemiol 2012;26(Suppl 1):205–22. 10.1111/j.1365-3016.2012.01282.x 22742612

[9] Ruel MT, Quisumbing AR, Balagamwala M Nutrition-sensitive agriculture: What have we learned so far? Glob Food Sec 2018;17:128–53. 10.1016/j.gfs.2018.01.002

[10] Harris J, Thompson S, Sparling T Leveraging Development Programs: Homestead Food Production : Ferranti P, Berry EM, Anderson JR, Encyclopedia of food security and sustainability. Oxford: Elsevier, 2019:396–400.

[11] James P Grant School of Public Health (JPGSPH). State of Food Security and Nutrition in Bangladesh: 2014. Dhaka, Bangladesh: HKI and JPGSPH, 2016.

[12] Gottlieb CA, Maenner MJ, Cappa C, et al Child disability screening, nutrition, and early learning in 18 countries with low and middle incomes: data from the third round of UNICEF’s Multiple Indicator Cluster Survey (2005-06). Lancet 2009;374:1831–9. 10.1016/S0140-6736(09)61871-7 19944864

[13] Bangladesh Bureau of Statistics, Ministry of Planning and United Nations Children’s Fund. Monitoring the Situation of Children and Women, Bangladesh: Multiple Indicator Cluster Survey 2009. 2010.

[14] Geographical Concentration of Rural Poverty in Bangladesh. Dhaka, Bangladesh: Center for Policy Dialogue, 2004.

[15] National Institute of Population Research Training - NIPORT/Bangladesh, Mitra Associates, ICF International. Bangladesh Demographic and Health Survey 2014. Dhaka, Bangladesh: NIPORT, Mitra and Associates, and ICF International, 2016.

[16] Dewey KG, Adu-Afarwuah S Systematic review of the efficacy and effectiveness of complementary feeding interventions in developing countries. Matern Child Nutr 2008;4(Suppl 1):24–85. 10.1111/j.1740-8709.2007.00124.x 18289157 PMC6860813

[17] Ivers NM, Halperin IJ, Barnsley J, et al Allocation techniques for balance at baseline in cluster randomized trials: a methodological review. Trials 2012;13:120 10.1186/1745-6215-13-120 22853820 PMC3503622

[18] Lorenz E, Gabrysch S Covariate-constrained randomization routine for achieving baseline balance in cluster-randomized trials. Stata J 2017;17:503–10. 10.1177/1536867X1701700214

[19] Helen Keller International. Homestead Food Production. http://www.hki.org/reducing-malnutrition/homestead-food-production/ (Accessed 9 April 2019).

[20] Olney DK, Vicheka S, Kro M, et al Using program impact pathways to understand and improve program delivery, utilization, and potential for impact of Helen Keller International’s homestead food production program in Cambodia. Food Nutr Bull 2013;34:169–84. 10.1177/156482651303400206 23964390

[21] Nielsen JN, Olney DK, Ouedraogo M, et al Process evaluation improves delivery of a nutrition-sensitive agriculture programme in Burkina Faso. Matern Child Nutr 2018;14:e12573 10.1111/mcn.12573 29278449 PMC6865971

[22] Talukder A, Osei AK, Haselow NJ, et al Contribution of homestead food production to improved household food security and nutrition status—lessons learned from Bangladesh, Cambodia, Nepal and the Philippines : Thompson B, Amoroso L, Improving diets and nutrition: food-based approaches. Rome, Italy: CABI International and FAO, 2014.

[23] Scaling-up “Biochar-urine nutrient cycling for health” in Bangladesh (BUNCH2Scale). 2017 http://knowledge4food.net/research-project/bangladesh-bunch2scale/ (Accessed 9 April 2019).

[24] World Health Organization (WHO). Essential Nutrition Actions: Improving maternal, newborn, infant and young child health and nutrition. Geneva, Switzerland: WHO, 2013. 25473713

[25] Hillenbrand E Transforming gender in homestead food production. Gender and Development 2010;18:411–25.

[26] FAO. FHI 360. Minimum Dietary Diversity for Women: a guide to measurement. Rome, Italy: FAO, 2016.

[27] Cox JL, Holden JM, Sagovsky R Detection of postnatal depression. Development of the 10-item Edinburgh Postnatal Depression Scale. Br J Psychiatry 1987;150:782–6. 3651732 10.1192/bjp.150.6.782

[28] Gausia K, Hamadani JD, Islam MM, et al Bangla translation, adaptation and piloting of Edinburgh Postnatal Depression Scale. Bangladesh Med Res Counc Bull 2007;33:81–7. 10.3329/bmrcb.v33i3.1138 18783062

[29] World Health Organization (WHO). Indicators for assessing infant and young child feeding practices: Part 2 Measurement. Geneva, Switzerland: WHO, 2010.

[30] WHO Multicentre Growth Reference Study Group. WHO Motor Development Study: windows of achievement for six gross motor development milestones. Acta Paediatr Suppl 2006;450:86–95. 16817682 10.1111/j.1651-2227.2006.tb02379.x

[31] Ahmed AU, Ahmad K, Chou V, et al The Status of Food Security in the Feed the Future Zone and Other Regions of Bangladesh: Results from the 2011-2012 Bangladesh Integrated Household Survey. Dhaka: International Food Policy Research Institute, 2013.

[32] World Health Organization (WHO). WHO child growth standards: length/height-for-age, weight-for-age, weight-for-length, weight-for- height and body mass index-for-age: methods and development. Geneva, Switzerland: WHO, 2006.

[33] WHO Anthro for personal computers: Software for assessing growth and development of the world’s children. 3.2.2 ed. Geneva: WHO, 2010.

[34] World Health Organization (WHO). WHO guidelines on drawing blood: best practices in phlebotomy. Geneva, Switzerland: WHO, 2010. 23741774

[35] Cogill B Anthropometric indicators measurement guide. Washington, D.C: Food and Nutrition Technical Assistance Project, Academy for Educational Development, 2003.

[36] Cashin K, Oot L Guide to anthropometry: a practical tool for program planners, managers, and implementers. 360 Washington, D.C: Food and Nutrition Technical Assistance III Project (FANTA) / FHI, 2018.

[37] Hayes RJ, Moulton LH Cluster Randomised Trials: Chapman & Hall/CRC. 2009.

