# Supplementary material for: Food and Agricultural Approaches to Reducing Malnutrition (FAARM): protocol for a cluster-randomised controlled trial to evaluate the impact of a Homestead Food Production programme on undernutrition in rural Bangladesh
Source: BMJ Open. 2019 Jul 4;9(7):e031037. doi: 10.1136/bmjopen-2019-031037 (PMC6615849; doi:10.1136/bmjopen-2019-031037)
Supplement: Supplementary data [file bmjopen-2019-031037supp001.pdf]

Supplementary File 1: Food Hygiene to reduce Environmental Enteric Dysfunction (FHEED)

In addition to promoting homestead gardens and nutrition education, a special behavior change intervention has been designed to strengthen the food hygiene component in FAARM. This food hygiene intervention uses a behavior-centered design by employing emotional drivers (such as pride, disgust and nurture) as well as engaging group activities (including games and competitions) and repeated individual household visits to improve common food hygiene practices among study participants.

The intervention focuses on the improvement of six identified key optimal feeding and food hygiene behaviors: exclusive breastfeeding up to 6 months, improved diversity and nutritional value of food (to achieve integration with the Homestead Food Production messages), cleanliness of serving utensils, proper hand-washing before feeding/eating, adequate food storage, and thorough reheating of stored food.

The food hygiene intervention is delivered to groups of 5 to 25 women by eight food hygiene promoters through two core components: group events, which facilitate exchange and interactive discussions, and individual household visits, which provide the opportunity of one-on-one counselling on key behaviors and individual questions. In total, the food hygiene promoters conducted four group events per women group and four household visits per woman over an eight-month period (July 2017 to February 2018).

The FHEED study was added as sub-study to the FAARM trial to evaluate the impact of food hygiene on the development of intestinal infections and Environmental Enteric Dysfunction (EED), as a second key pathway to child growth alongside nutrition. It specifically studies factors on the pathway from food hygiene to EED, including women´s food hygiene behaviour (measured through structured observation), complementary food contamination (log-transformed counts of total coliforms and *E.coli* in food samples), intestinal pathogen load (pathogen-specific qRT-PCR), diarrheal episodes and EED biomarkers (fecal myeloperoxidase, fecal α1-antitrypsin fecal neopterin, serum C-reactive protein, serum α1-acid glycoprotein). The FHEED study will assess these parameters in children aged 0-18 months in two cross-sectional surveys (in 2018 and 2019). In addition, children born in 2018 after the end of the food hygiene intervention will be enrolled in a cohort and sampled every two months over a 12-month period.
