# Supplementary material for: Food and Agricultural Approaches to Reducing Malnutrition (FAARM): protocol for a cluster-randomised controlled trial to evaluate the impact of a Homestead Food Production programme on undernutrition in rural Bangladesh
Source: BMJ Open. 2019 Jul 4;9(7):e031037. doi: 10.1136/bmjopen-2019-031037 (PMC6615849; doi:10.1136/bmjopen-2019-031037)
Supplement: Supplementary data [file bmjopen-2019-031037supp002.pdf]

Table S1: Surveillance indicators collected during routine assessment of the FAARM trial in Sylhet, Bangladesh

Category	*Indicator*	*Definition*	*Frequency of collection*
Pregnancy & birth identification	Last menstrual period	First day of last menstrual period	Every two months
	Pregnancy status	Identification of new, continued, or loss of pregnancy	Every two months
	Newborn identification	Newborn date of birth record	Every two months
Prenatal and postnatal health	Intended pregnancy	If the woman had intended to get pregnant at this time	Once pregnancy is identified
	ANC attendance	Antenatal care-seeking and home visits by health workers	Every two months during pregnancy
	ANC services	Woman's weight taken, blood pressure measured, urine sample taken, blood sample taken, ultrasonography done, child position checked, eye check for anemia, tetanus toxoid injection given	Every two months during pregnancy
	Iron and folic acid consumption	Intake of iron and folic acid in the previous 7 days	Every two months during pregnancy
	Resting time	Hours of rest and sleep in the previous 24 hours	Every two months during pregnancy
	Night blindness	Difficulty seeing at dusk or night	Every two months during pregnancy
	Pregnancy complications	Reporting of twins, severe headaches, high blood pressure, etc.	Once per pregnancy (≥ 6 months of gestation)
	Past complications	Reporting of past pregnancy complications	Asked once during first reported pregnancy

	Birth delivery plan	Intended place of delivery	Once per pregnancy (≥ 6 months), confirmed in follow-up visits
	Depression	Edinburgh Postnatal Depression Scale (adapted to Bangladesh)[1]	Once during pregnancy (≥ 6 months gestation), once when child is born and once when child is 6-12 months of age
Women's nutrition	Dietary diversity	Information on 21 food groups[2 3]	Every 6 months for all women<br>Every 2 months during pregnancy
	Weight	Weight of women in kg using Tanita scales	Every 12 months for all women<br>Every 2 months during pregnancy
Children's nutrition	IYCF	WHO IYCF indicators[4]	
	Dietary diversity	Information on 21 food groups[2 3]	
	Weight	Weight of children in kg using Tanita scales	When child is approximately 18 months of age
Child morbidity	Diarrhea	7-day period prevalence; 2-day point prevalence	Every two months
	Respiratory distress	7-day period prevalence	Every two months
	Sick child feeding & healthcare seeking	If child was taken to health care facility, child feeding behaviors	Every two months, when child was reported as ill
Child development	Motor Milestones	Child performance of developmental motor milestones according to age[5]	When child reaches appropriate age for milestone
	Language	Child language questionnaire adapted from Frongillo et al, 2017[6]	Every two months when child is 5-11 months of age
Agriculture	Crop diversity	Listing of vegetables, fruits, and spices grown in the past growing season.	

ANC: antenatal care; **IYCF**: Infant and young child feeding;

References:

1. Gausia K, Hamadani JD, Islam MM, et al. Bangla translation, adaptation and piloting of Edinburgh Postnatal Depression Scale. *Bangladesh Medical Research Council Bulletin* 2007;33(3):81-7. [published Online First: 2008/09/12]
2. Wiesmann D, Arimond M, Loechi C. Dietary diversity as a measure of the micronutrient adequacy of women's diets: results from rural Mozambique site. Washington, DC: Food and Nutrition Technical Assistance II Project, FHI 360, 2009.
3. FAO, FHI 360. Minimum Dietary Diversity for Women: A Guide to Measurement. Rome, Italy: FAO 2016.
4. World Health Organization (WHO). Indicators for assessing infant and young child feeding practices: Part 2 Measurement. Geneva, Switzerland: WHO 2010.
5. WHO Motor Development Study: windows of achievement for six gross motor development milestones. *Acta Paediatrica (Oslo, Norway: 1992) Supplement* 2006;450:86-95. [published Online First: 2006/07/05]
6. Frongillo EA, Nguyen PH, Saha KK, et al. Large-Scale Behavior-Change Initiative for Infant and Young Child Feeding Advanced Language and Motor Development in a Cluster-Randomized Program Evaluation in Bangladesh. *The Journal of Nutrition* 2017;147(2):256-63. doi: 10.3945/jn.116.240861 [published Online First: 2016/12/30]
