# Supplementary material for: Food and Agricultural Approaches to Reducing Malnutrition (FAARM): protocol for a cluster-randomised controlled trial to evaluate the impact of a Homestead Food Production programme on undernutrition in rural Bangladesh
Source: BMJ Open. 2019 Jul 4;9(7):e031037. doi: 10.1136/bmjopen-2019-031037 (PMC6615849; doi:10.1136/bmjopen-2019-031037)
Supplement: Supplementary data [file bmjopen-2019-031037supp003.pdf]

Table S2: FAARM trial measures of anthropometry, biomarkers, and food contaminants

Category	*Indicator*	*Time of measurement*	*Equipment / Laboratory test*
Anthropometry	Woman weight	Baseline;<br>Routine Assessment (once per year, every 2 months during pregnancy);<br>Endline	Baseline/Endline: SECA 874 digital floor scale<br>RA: Tanita weight scales
	Woman height	Baseline;<br>Endline[a]	SECA 217 stadiometer
	Woman MUAC	Routine Assessment<br>(every 2 months during pregnancy)	SECA 212 measuring tape
	Child weight	Baseline;<br>Routine Assessment<br>(around 18 months);<br>Endline	Baseline/Endline: SECA 874 digital floor scale<br>RA: SECA 334
	Child length	Baseline;<br>Birth Registration (newborn length);<br>Endline	Baseline/Endline: SECA 416 digital baby scale<br>BR: SECA 232 measuring rod (until March 2017); locally made length board (from April 2017)
	Child MUAC	Birth Registration	SECA 212
	Child head circumference	Birth Registration	SECA 212
Biomarkers	Hemoglobin	Baseline;<br>Endline	Hemocue 201+<br>Sysmex XP-100 Hematology Analyzer
	Serum ferritin[b]	Baseline;<br>Endline	Sandwich ELISA[1]
	Serum transferrin receptor[b]	Baseline;<br>Endline	Sandwich ELISA[1]

	Retinol-binding protein[b]	Baseline; Endline	Sandwich ELISA[1]
	C-reactive protein[b]	Baseline; Endline	Sandwich ELISA[1]
	Serum α-1-acid glycoprotein[b]	Baseline; Endline	Sandwich ELISA[1]
	Serum zinc[b]	Endline	Colorimetric method
	Feacal myeloperoxidase[b]	2018 (Food hygiene evaluation); Endline	Sandwich ELISA[1]
	Feacal α-1-antitrypsin[b]	2018 (Food hygiene evaluation); Endline	Sandwich ELISA[1]
	Feacal neopterin[b]	2018 (Food hygiene evaluation); Endline	Sandwich ELISA[1]
Food contaminants	Coliforms[b]	2018 (Food hygiene evaluation)	Pour plating on Tryptone Bile X-Glucuronide (TBX) Agar
	Escherichia coli[b]	2018 (Food hygiene evaluation)	Pour plating on Tryptone Bile X-Glucuronide (TBX) Agar

[a] For those women who were not measured at baseline. [b] These measurements will be conducted in a sub-sample of the population.
MUAC: mid-upper arm circumference

1. Erhardt JG, Estes JE, Pfeiffer CM, et al. Combined measurement of ferritin, soluble transferrin receptor, retinol binding protein, and C-reactive protein by an inexpensive, sensitive, and simple sandwich enzyme-linked immunosorbent assay technique. *The Journal of Nutrition* 2004;134(11):3127-32. doi: 10.1093/jn/134.11.3127
