# Supplementary material for: Food and Agricultural Approaches to Reducing Malnutrition (FAARM): protocol for a cluster-randomised controlled trial to evaluate the impact of a Homestead Food Production programme on undernutrition in rural Bangladesh
Source: BMJ Open. 2019 Jul 4;9(7):e031037. doi: 10.1136/bmjopen-2019-031037 (PMC6615849; doi:10.1136/bmjopen-2019-031037)
Supplement: Supplementary data [file bmjopen-2019-031037supp004.pdf]

Supplementary File 2:

Blood samples:

During the baseline survey in 2015, 300 µL of whole capillary blood was collected (in Microvette^{TM} tubes) from enrolled women and children. Blood samples were then transported to the field laboratory maintaining a cold chain (+4°C to +8°C at all times) and stored in a refrigerator at 4°C over night. The following morning, samples were centrifuged in an Eppendorf Mini-Spin centrifuge for 10 minutes at 13,400 rounds per minute. The supernatant was pipetted into 50 µL aliquots (approximately 2-3 per sample). Serum and red blood cells were then stored in a -20°C freezer on site. Samples that were transported to Germany on dry ice were again stored in a -20°C freezer before analysis.

At the endline survey in 2019, we will collect venous blood from enrolled woman (in total 8 mL) and children (in total 5 mL) into the Vacutainer^{TM} collection system EDTA and serum tubes, through puncture of a cubital vein. Blood samples will be transported to the field laboratory maintaining a cold chain (+4°C to +8°C at all times) and stored in a refrigerator at 4°C over night. The following morning, EDTA samples will be used for complete blood count analysis. Serum and red blood cells will be stored in a -20°C freezer on site. Samples will then be transported to Germany on dry ice and again stored in a -20°C freezer before analysis.

Faecal samples:

Stool samples are collected during a cross-sectional survey in all children aged 0-18 months in 2018 and at study endline in 2019. In a subset (150 households), we additionally collect maternal stool samples. Stool collection sets (stool collection tubes, a sterile stick for stool collection and gloves) are handed to the mothers with instructions one day prior to collection. We collect one native stool sample and one stool sample preserved in 7 mL RNA-later for nucleic acid extraction. Stool samples are aliquoted and stored in a -20°C freezer on site until shipment to Germany on dry ice for further analysis and long-term storage at -80°C.
