# Supplementary material for: Food and Agricultural Approaches to Reducing Malnutrition (FAARM): protocol for a cluster-randomised controlled trial to evaluate the impact of a Homestead Food Production programme on undernutrition in rural Bangladesh
Source: BMJ Open. 2019 Jul 4;9(7):e031037. doi: 10.1136/bmjopen-2019-031037 (PMC6615849; doi:10.1136/bmjopen-2019-031037)
Supplement: Author's manuscript [file bmjopen-2019-031037.draft_revisions.pdf]

BMJ Open is committed to open peer review. As part of this commitment we make the peer review history of every article we publish publicly available.

When an article is published we post the peer reviewers' comments and the authors' responses online. We also post the versions of the paper that were used during peer review. These are the versions that the peer review comments apply to.

The versions of the paper that follow are the versions that were submitted during the peer review process. They are not the versions of record or the final published versions. They should not be cited or distributed as the published version of this manuscript.

BMJ Open is an open access journal and the full, final, typeset and author-corrected version of record of the manuscript is available on our site with no access controls, subscription charges or pay-per-view fees (http://bmjopen.bmj.com).

If you have any questions on BMJ Open's open peer review process please email info.bmjopen@bmj.com

BMJ Open

Food and Agricultural Approaches to Reducing Malnutrition (FAARM): Protocol for a cluster-randomised controlled trial to evaluate the impact of a Homestead Food Production programme on undernutrition in rural Bangladesh

Journal:	*BMJ Open*
Manuscript ID	bmjopen-2019-031037
Article Type:	Protocol
Date Submitted by the Author:	12-Apr-2019

Complete List of Authors:	Wendt, Amanda; University of Heidelberg, Heidelberg Institute of Global Health<br>Sparling, Thalia M; University of Heidelberg, Heidelberg Institute of Global Health<br>Waid, Jillian; University of Heidelberg, Heidelberg Institute of Global Health; Hellen Keller International<br>Mueller, Anna; University of Heidelberg, Heidelberg Institute of Global Health<br>Gabrysch, Sabine; University of Heidelberg, Heidelberg Institute of Global Health

Keywords:	Nutrition < TROPICAL MEDICINE, Community child health < PAEDIATRICS, PUBLIC HEALTH, EPIDEMIOLOGY

SCHOLARONE™
Manuscripts

Title:
Food and Agricultural Approaches to Reducing Malnutrition (FAARM): Protocol for a cluster-randomised controlled trial to evaluate the impact of a Homestead Food Production programme on undernutrition in rural Bangladesh

Authors: Amanda S. Wendt, Thalia M. Sparling, Jillian L. Waid, Anna A. Müller, Sabine Gabrysch

Addresses / affiliations:
Heidelberg Institute of Global Health, Heidelberg University, 69120 Heidelberg, Germany

Amanda S. Wendt
Postdoctoral researcher

Thalia M. Sparling
Postdoctoral researcher

Jillian L. Waid
Doctoral student

Anna A. Müller
Postdoctoral researcher

Sabine Gabrysch
Associate professor

Helen Keller International, Bangladesh Country Office, Dhaka 1212, Bangladesh

Jillian L. Waid
Director, Research, Learning and Evaluation

Correspondence to: Sabine Gabrysch, sabine.gabrysch@uni-heidelberg.de

Word count: 5,443

Abbreviations:

BMI: Body mass index
FAARM: Food and Agricultural Approaches to Reducing Malnutrition
HFP: Homestead Food Production
HKI: Helen Keller International
NGO: Non-governmental organization
VARD: Voluntary Association for Rural Development
WHO: World Health Organization
UNICEF: United Nations Children's Fund

For peer review only

Abstract

Introduction: Chronic undernutrition affects over 150 million children worldwide and has serious consequences. The causes are complex and include insufficient dietary diversity and poor hygiene practices. Systematic reviews of nutrition-sensitive agricultural interventions concluded that while these hold promise, there is insufficient evidence for their impact on child growth. The Food and Agricultural Approaches to Reducing Malnutrition (FAARM) project is a 1:1 cluster-randomised trial aiming to evaluate the impact of a Homestead Food Production (HFP) programme implemented by Helen Keller International (HKI) on women's and children's undernutrition.

Methods and analysis: The HFP intervention comprises training of women's groups and asset distribution to support year-round home gardening, poultry rearing, and improved nutrition and hygiene practices. Formal trainings are supplemented by behaviour change communication during household visits, and facilitated links between producer groups and market actors. The FAARM trial will examine if and how this complex intervention reduces undernutrition. In 2015, FAARM enrolled married women and their children (0-3 years) in 96 rural settlements of Habiganj district in Sylhet division, Bangladesh. Covariate-constrained randomization was used to assign 48 settlements to receive a three-year HFP intervention, with the other 48 acting as controls, targeting over 2700 women. To study impact pathways, a surveillance system collects data on all participants every two months. In late 2019, children 0-3 years of age (born during the intervention period) will be surveyed, thus capturing impact during the critical first 1,000 days of life. Children's length-for-age z-scores will be compared between intervention and control arms using mixed effects linear regression. Secondary outcomes include women's and children's micronutrient status, dietary intake, dietary diversity, and other indicators of child growth, development, and morbidity.

For peer review only

Ethics and dissemination: Ethical approval was received in Bangladesh and Germany. Results will be disseminated through peer-reviewed publications and presentations in Bangladesh and internationally.

Trial Registration: ClinicalTrials.gov ID: NCT02505711. Registered: 22 July 2015.

Strengths and limitations of this study

- The trial includes 96 clusters and used covariate-constrained randomization, thus ensuring good baseline balance.
- We evaluate programme impact on children conceived after the start of the intervention who will have benefitted during their full first 1,000 days of life.
- A wide range of knowledge, behavioural, anthropometric, and biological indicators will be assessed in women and children.
- Rigorous monitoring and surveillance systems provide a platform to examine outputs and outcomes along the causal pathway.
- We measure child growth and blood parameters at baseline in early 2015 and at endline in late 2019, but not continuously over the course of the trial.

For peer review only

Introduction

Child undernutrition is responsible for an estimated 45% of child mortality, about 3.1 million deaths annually (1). Suboptimal nutrition during the first 1,000 days, from conception to two years of age, can have irreversible consequences for child development and long-term health, intelligence, educational attainment and work capacity (1, 2).

To reduce undernutrition sustainably, one must address its complex root causes. The two main direct causes according to the UNICEF and Lancet frameworks are nutrient-deficient diets and frequent gastrointestinal infections (1, 3). An inadequate diet of the mother limits the nutrients the child receives during pregnancy and from breastfeeding, and an inadequate diet of the young child further contributes to undernutrition (1). Nutrient-deficient diets of women and children are often due to insufficient household and maternal knowledge and resources, while unhygienic living conditions favour gastrointestinal infections (1, 3). In addition to the short-term negative influence of infections on appetite and nutrient absorption, frequent subclinical gastrointestinal infections may cause chronic inflammation and damage to the mucosal barrier in the gut and disrupt the intestinal microbiome, leading to "environmental enteric dysfunction", further inhibiting the absorption of nutrients (4).

Due to the many complex causes of undernutrition, programmes that address several underlying factors of child nutrition and health are needed. However, the evidence on "potentially promising and culturally relevant" interventions to diversify diets by "enhancement of agricultural production" has been weak, because most such interventions "have only been implemented at a small scale, and have not been adequately assessed", according to The Lancet's 2008 undernutrition series (5). Evaluations of nutrition-sensitive agricultural interventions, i.e. agricultural interventions focusing not only on yields, but on improving nutrition of vulnerable groups, have been relatively rare, leading to a gap in our understanding of their potential contribution to reducing child undernutrition (6).

For peer review only - http://bmjopen.bmj.com/site/about/guidelines.xhtml

Previous evaluations of nutrition-sensitive agriculture programmes found that these can improve household access and consumption of targeted foods and in some cases improve household, maternal, or child dietary diversity, however, the evidence of their impact on child anthropometric outcomes was very weak, according to several reviews in 2012-2013 (6-8). This lack of evidence was largely attributed due to methodological shortcomings of previous studies, such as lack of appropriate control groups and small sample sizes (6-8).

In 2018, Ruel et al. evaluated the recent literature (since 2014) and found several studies which had improved methodologies and examined a wide range of nutrition-sensitive agricultural interventions, including eight homestead food production programmes (9). Ruel et al. report that most studies showed increases in targeted food production, household and child dietary diversity, and in micronutrient intake when measured. Some also showed impacts on anaemia or micronutrient status. However, evidence on stunting was still lacking. Possible reasons cited were short programme durations, poor age targeting (outside the critical 1,000 days), and insufficient statistical power to detect changes in stunting prevalence (9, 10).

With the Food and Agricultural Approaches to Reducing Malnutrition (FAARM) trial, we attempt to address these limitations through a strong cluster-randomised design, sufficient sample size and duration, recruitment strategies to target children in their first 1,000 days, as well as measuring a wide range of indicators along the causal pathway.

Study aims and objectives

The study will evaluate the impact of a Homestead Food Production (HFP) programme implemented by Helen Keller International (HKI) on length-for-age z-score among children aged 0-3 years who were conceived during the intervention period.

For peer review only - http://bmjopen.bmj.com/site/about/guidelines.xhtml

Methods and analysis

Study design

The FAARM trial is a 1:1 cluster-randomised controlled trial with repeated cross-sectional surveys of children born to a cohort of women. To cover the full first 1,000 days of life, we enrolled young, married women into the HFP programme in 2015, with the target group at endline in 2019 being children under 3 years of age conceived by these women during the intervention period.

Study setting

The FAARM trial is conducted in Bangladesh where prevalence of food insecurity and undernutrition is high. In 2014, over half of women consumed inadequately diverse diets, almost a fifth had a BMI below 18.5, and 35% of children under five years were stunted (11). Child marriage and early childbearing are also common in the country: almost half of women had their first pregnancy by age 18 (11). Only 20% of pregnant women receive adequate antenatal care and about 40% of mothers practice exclusive breastfeeding during the first six months (11). Complementary feeding practices for children aged 6-23 months are poor with only 43% receiving an adequately diverse diet (11). In addition, women in Bangladesh and their families struggle with persistent poverty, gender inequality and lack of women's agency, climate change, and political volatility.

Within Bangladesh, the study area was identified through an analysis of population demographics, including proxies for wealth, results from national nutrition surveys, and information about other interventions in the area. Sylhet Division, in north-eastern Bangladesh, has a prevalence of undernutrition above the country average, with 44% of children under five stunted and 40% of pregnant women undernourished in 2014 (11). Sylhet

For peer review only

For peer review only - http://bmjopen.bmj.com/site/about/guidelines.xhtml

Division also has the largest wealth disparity and the highest fertility in Bangladesh, and performs worst on a range of gender indicators (12-15).

Sample size

The study sample size was calculated in relation to the primary outcome, length-for-age. A continuous z-score was used, i.e. length in standard deviations below or above the mean for a child of a given age and sex, which can then be translated into the percentage of children "moderately or severely stunted" (below -2 standard deviations). We aimed to detect a minimum difference of 0.20 to 0.25 in mean z-score at a 5% significance level. This effect size has been seen in studies using educational approaches to improve child growth (16).

The sample size for each study arm was first calculated for a simple randomized design and then multiplied by a design factor to account for the clustered design and the greater similarity of children's nutritional status within a cluster. The intra-cluster correlation of length-for-age z-score was estimated to be 0.03, and for a minimum detectable effect size of 0.22 with 90% power, sample size was calculated as 677 children per arm, assuming a cluster size of 15. To account for loss to follow-up, possibly due to late refusal, withdrawal from the study or permanent migration out of the study area, an extra 10% was added. Therefore, we aimed for approximately 745 children per arm, 1490 children in total. Assuming an average of 15 children per cluster, born to 28 women, this meant that nearly 100 clusters needed to be randomized.

Participant recruitment and cluster formation

Once the study site in Baniachong and Nabiganj sub-districts of Habiganj district, Sylhet division, Bangladesh had been selected, we enumerated all households in the area to identify

For peer review only

women eligible to participate in FAARM. A woman was eligible for inclusion if she reported to be less than 30 years old[1], was married and her husband stayed overnight at the household at least once in the year prior to interview, had access to at least 40 square meters of land (ideally 10 square meters near the house) and was interested to participate in a homestead food production programme (Figure 1).

Once eligible women were identified, clusters (the unit of randomization) were formed with at least 10 and no more than 65 eligible women, based on geographic location of settlements. A minimum distance of 400 meters between settlements was maintained to limit contamination. Figure 2 shows the study site within Bangladesh, and the 96 clusters within 13 unions of Nabiganj and Baniachong sub-districts.

At the start of the baseline survey, trained data collectors approached eligible women for consent who had been assigned to a cluster (Figure 3). Some women were not enrolled into the trial because they had migrated since enumeration, were repeatedly not at home at the time of the survey visits, or refused to participate. During the baseline survey, age was again ascertained using event calendars to get more accurate estimates. In cases where the newly estimated age at baseline was over 30 years, we did not exclude these women from the trial. The youngest biological child of an enrolled woman was assessed at baseline if the child was born after March 1, 2012, three years prior to the first day of the baseline survey training.

During the first year of implementation, we additionally invited women (and their youngest biological children) to enrol who had married into households already participating in the trial

[1] With limited birth records, and official birth records frequently incorrect, many adults cannot accurately report their age.

For peer review only - http://bmjopen.bmj.com/site/about/guidelines.xhtml

(Figure 1). The number of women enrolled in this way was nearly identical in the intervention and control groups.

Cluster randomization

After the baseline survey, 96 clusters were randomly assigned to the intervention or control group (Figure 3). We used covariate-constrained randomization to ensure good balance, i.e. that important baseline characteristics do not differ greatly between arms (17, 18). The randomization was constrained by covariates on the cluster, household, woman, and child level.[2]

The intervention

HKI has been implementing HFP programmes in Bangladesh since 1988, working in partnership with over 70 local non-governmental organizations (NGOs) (19). The objectives of the HFP programmes are to increase household access and consumption of nutrient-dense foods through gardening, poultry rearing and nutrition counselling, and to empower women through increased control of assets and through group leadership and participation. Together these changes are thought to improve the health and nutrition of women and children. Over time, HKI has adapted the programme based on community needs and evolving evidence and expanded to several countries in Africa and Asia (20-22).

Similar to earlier HFP projects, FAARM's HFP programme is implemented jointly with a local partner NGO, the Voluntary Association for Rural Development (VARD). Three full-time technical staff from HKI manage the local project office and provide support and direction to eight full-time project staff, a project coordinator and an accountant from VARD.

[2] Cluster: union and number of women; Household: religion, food consumption score, household food insecurity access score, log of agricultural land size; Women: height, BMI, haemoglobin; Child: age, age-adjusted z-scores of length-for-age and weight-for-length, haemoglobin, diarrhoea prevalence.

For peer review only - http://bmjopen.bmj.com/site/about/guidelines.xhtml

Each project staff member is responsible for a certain geographic region for which they have monthly targets for trainings, individual counselling and asset distribution.

Prior to programme activities, 64 households with existing home gardens were visited in six villages in the project area, interviewed on previous experiences and all food crop species were identified by agricultural researchers. Most gardens had few species and plants and were only active few months per year. Key issues reported were pests, poor soil fertility, excessive shade, and flooding exposure (preventing year-round gardening). Strategies to overcome these were incorporated into the HFP curriculum.

Programme activities begin with organizing project participants into village-level “women farmer groups” of around 16 households each (range from 8 to 26). This is the key intervention unit. Within these groups, members select a lead farmer family, who oversees a village model farm on their property and acts as a multiplier. The lead family receives additional training and is encouraged to implement all recommended improved techniques, produce seeds and seedlings for distribution to other participants, and host trainings and meetings. In larger clusters where one village model farm may provide for several groups, additional women serve as group leaders. At project closeout, at least one motivated participant in each group will be selected as a peer educator to remind neighbours of promoted activities after the intervention has concluded.

FAARM’s HFP intervention includes a series of trainings, usually held at the village model farm. All participants will receive trainings on year-round vegetable gardening, fruit tree production, poultry rearing, and the implications of household production and consumption on health. In the first year, one-day trainings are given on winter and summer crop cultivation, seed production and preservation, and poultry rearing, while half-day refresher trainings are

For peer review only

given in subsequent years. The trainings emphasize sustainable gardening techniques, including the use of raised beds, live fencing, integrated pest management, composting and soil management, and seed preservation. In the third year of the intervention, once homestead production and consumption are established and there is surplus production, marketing training is given to build linkages with local vendors in order to provide opportunities for income generation for women. In response to issues previously identified in the area, additional trainings are given on sack gardening (during the rainy season when gardens are prone to flooding) and on production and application of urine-enriched biochar fertilizer (to enhance soil fertility), implemented as part of a separate study (23).

Furthermore, project staff conduct courtyard sessions every two months for participants and family members, covering nutrition, health and hygiene topics. An adult learning approach is used, which includes dialogue, story-telling, role play and drawing as well as joint activities such as food preparation. Session content follows the global Essential Nutrition Actions approach, including optimal breastfeeding, infant and young child feeding and nutrition for women (24).

In addition to formal trainings and meetings, project staff visit all participants individually in their households every two months to review nutrition, health and hygiene messages as well as provide technical support for gardening and poultry rearing activities. These interpersonal counselling sessions provide opportunities for participants to bring up issues they are facing that may be difficult to discuss in a group setting and for project staff to tailor messages to the unique situation of the participant and her family.

In the third year of the intervention, an additional food hygiene training is added to reinforce messages on dietary diversity and cover food-related hygiene topics more intensively, in

For peer review only

particular hand-washing, clean utensils, food storage, and re-heating (see Supplementary File 1). This intervention is delivered by eight female staff and consists of eight structured events, four group events and four household visits.

FAARM's HFP intervention also transfers assets. Seeds are distributed to all project households twice a year to coincide with the main planting seasons and include nutrient-rich, seasonally and locally appropriate vegetables such as red amaranth, water spinach, tomato, pumpkin, string bean and cabbage. Similarly, tree saplings, chosen for nutrient-rich fruits such as papaya, lemon and jujube, were transferred to households with greater land availability. Participants also receive poultry assets, primarily a reimbursement for the majority of the cost of a constructed, improved poultry shed, as well as starter feed and watering stations.

A gender-equitable approach is integrated into the HFP structure with the programme aiming to increase women's status in the household through asset transfer and control of productive resources as well as the skill transfer associated with HFP trainings. Improving women's status and agency is thought to further support programme activities and improve child nutrition (25).

Programme theory

A conceptual framework (Figure 4) was constructed to illustrate the HFP programme's theory of change. Training on gardening and poultry rearing can increase household access to nutrient-dense foods, counselling on health, hygiene, and child care topics may lead to improved practices, and the marketing of surplus products can increase household income. Throughout the programme, the focus on women's participation and leadership may increase women's empowerment by increasing their decision-making role and social support. When

For peer review only

these outcomes are achieved, we expect to see improvements in dietary intake and reduced infections, leading to reduced maternal and child undernutrition and better child development.

Control arm

In both intervention and control settlements, there are certain benefits delivered alongside the data collection activities. These include free pregnancy tests, birth counselling to establish a birth plan, and a visit within three days of birth where staff weigh and measure the child and provide breastfeeding counselling to the mother.

Monitoring plan

FAARM's monitoring plan consists of three major elements: intervention activity records, output monitoring of intervention households, and a multi-part surveillance system that includes both intervention and control households (Figure 3). Indicators measured through these systems are outlined in a detailed project monitoring plan based on identified project impact pathways. All systems gather data using ODK Collect software (www.ona.io) on android mobile devices.

Intervention activity records

Project monitoring forms were designed to track programme contact points, including asset distribution, attendance of each participant and her family members at courtyard sessions and trainings, and tracking when and what topics were included in counselling sessions. In addition, supervisors document their monitoring of project staff activities.

Output monitoring

Project staff conduct monitoring interviews of FAARM intervention participants on a rolling basis, with each respondent visited once per year (Table 1). In these interviews, outputs and

For peer review only

outcomes, such as garden diversity and yields, poultry production, and knowledge gained from training modules are assessed in the intervention arm (Figure 4).

Surveillance

FAARM's surveillance system in intervention and control households contains three components explained in detail below: 1) routine assessment, 2) food consumption assessment, and 3) birth registration (Figure 3). It serves to examine indicators on the two main pathways, diet and infection, through which the intervention could impact child undernutrition, as well as assessing maternal undernutrition and intrauterine growth retardation (Figure 4). All surveillance activities are conducted by locally-recruited, female data collectors. Extensive training with field practice is provided before any data collector begins work. Before each two-month data collection period, a 1-2-day refresher training is held to review and troubleshoot as well as train on any questionnaire aspects that have been added or changed. Collected data are downloaded and reviewed regularly for real-time monitoring and analysis. The surveillance system is summarized in Table 1, while information on indicators collected is available in Table S1.

A routine assessment round is completed every two months. Women report their last menstrual periods using provided calendars to mark the day. Pregnancies are identified with the help of pregnancy tests, which are provided for free to women for voluntary self-administration. Pregnant women are asked about antenatal care, and their weight and mid-upper arm circumference are measured at every visit, while non-pregnant women are weighed once per year. Dietary diversity information is recorded from all pregnant women each round and from non-pregnant women every six months using 21 food groups (26). The Edinburgh Perinatal/Postnatal Depression Scale is administered during pregnancy, shortly after delivery, and after the child has attained six months of age (27, 28). For all children who are below three years of age (at the first day of each two-month round), we ask mothers about recent

For peer review only

For peer review only - http://bmjopen.bmj.com/site/about/guidelines.xhtml

diarrhoea and acute respiratory infection episodes. Mothers of children 0 to 18 months are asked all questions from the standard WHO Infant and Young Child Feeding survey module each round, while children above 18 months are asked these questions every six months (29). Starting in the second year of the trial, the six WHO motor milestones are measured on all children as appropriate for their age (30). Starting in the third year of the trial, all children have their weight recorded around 18 months of age. Every four months, women are asked about the different crop species grown in their gardens in the previous season (cool-dry, hot-dry, rainy) to assess diversity of production. Annually, questions are asked about dietary habits during Ramadan. Periodically, additional questions are added, e.g. about poultry production, garden characteristics, household hygiene, and food security.

Detailed quantitative food consumption information is recorded through dietary recall on a randomly selected subset of nine households per cluster, i.e. a total of 864 households. To achieve even distribution over seasons and arms, the 96 clusters were divided into two phases of 48 clusters, with 24 intervention and 24 control clusters in each phase. Data are collected every two months, i.e. six times per year, alternating between phases, with each cluster thus being visited three times per year to interview three households each time. Total food consumed by the household and how this was distributed among all household members is reported by the primary food preparer. A separate module about snacks and food intake between meals is asked to all available household members separately. In a third of the sample each round (one of the three households per cluster), diets are recorded on two non-consecutive days, less than ten days apart, in order to enable estimation of usual intake and the proportion of the population at risk of inadequate intake. The methods used in data collection are derived from those designed by International Food Policy Research Institute

For peer review only - http://bmjopen.bmj.com/site/about/guidelines.xhtml

(IFPRI) for the Bangladesh Integrated Household Survey [18]. This procedure is repeated annually in the same households in the same season.

Birth registration activities are initiated on all identified pregnancies in the third trimester. Pregnant mothers are provided with free phone cards and encouraged to notify the team when they have delivered. An automated calling list is used by birth registration team members to give reminder calls to the pregnant women before their estimated date of birth. As soon as notified, ideally within 72 hours of birth, a trained female data collector visits the family, measures the newborn's weight, length, and head circumference, interviews the mother on the delivery, and provides initial lactation counselling. Birth registration data collectors are trained on standard anthropometric measurement techniques for newborns and calibrate measurement equipment on each day of use.

For peer review only

Evaluation plan

Trial outcomes and measurements

Primary outcome

The primary outcome measure is length-for-age z-score in children under three years of age at endline, using the 2006 WHO child growth standards (31).

Secondary outcomes

Secondary outcome measures include other measures of child growth, namely weight-for-length and head circumference-for-age z-scores in children 0-3 years; and length-for-age z-score in children 6-30 months, as well as growth outcomes at birth (length-for-gestational-age, weight-for-gestational-age, and head circumference-for-gestational-age). For women’s

For peer review only - http://bmjopen.bmj.com/site/about/guidelines.xhtml

nutritional status, indicators are BMI in non-pregnant women, and mid-upper arm circumference and gestational weight gain in pregnant women. Micronutrient status is measured through anaemia (haemoglobin), vitamin A deficiency (retinol-binding protein), iron deficiency (serum ferritin, soluble transferrin receptor), and zinc deficiency (serum zinc) in women and children, adjusting for inflammation (serum C-reactive protein, serum α-1-acid glycoprotein) when recommended (Table S2 & Supplementary File 2).

Dietary diversity is measured according to published recommendations, using 10 food groups for women and 7 food groups for children (26, 29). In addition, dietary adequacy is assessed for a subsample of women and children using the mean probability of adequate intake for 11 micronutrients (thiamine, riboflavin, niacin, vitamin B6, folate, vitamin B12, vitamin C, vitamin A, calcium, iron, zinc). Indicators for infections in children comprise 7-day period and 2-day point prevalences of diarrhoea and of acute respiratory infection. Early child development is assessed using WHO motor milestones (30, 32).

Food hygiene indicators include a hygiene behaviour score measured through structured observation in mothers with children 6-18 months of age, faecal contamination (total coliforms and *E. coli*) of complementary food samples, and enteropathy biomarkers (faecal myeloperoxidase, faecal α1-antitrypsin, faecal neopterin, serum C-reactive protein, serum α-1-acid glycoprotein). This is conducted through the "Food Hygiene to reduce Environmental Enteric Dysfunction" study nested into the FAARM trial (see Supplementary File 1).

Additional outcomes

The FAARM trial provides an ideal platform on which to explore other research topics, such as linkages between food production and diets, women's agency and mental health, child language development, dietary influences on arsenic methylation capacity, mycotoxin exposure, (epi-)genetic factors influencing innate immunity and micronutrient metabolism, as well as diversity and composition of the mother's and the child's gut microbiome through

For peer review only

For peer review only - http://bmjopen.bmj.com/site/about/guidelines.xhtml

which the above effects may be modified. Several of these additional research components will be explored during the course of the trial, contingent on time and funding.

All measurements and samples are taken in accordance with current standards and methodology, building on questionnaire tools from HKI and James P Grant School of Public Health at BRAC University, including from the Food Security and Nutritional Surveillance Project in Bangladesh (11). HKI employs experienced field research assistants who receive specific training and supervision on anthropometric measurement and interview techniques. All blood sampling is undertaken by clinically trained staff, familiar with the appropriate methods of extraction, storage and processing.

Data collection

Most trial outcomes are measured during the endline survey in late 2019 (Figure 3). A week-long training is held for all data collectors, with separate trainings conducted for questionnaire interviewers, anthropometry and blood sampling staff, following standard measurement procedures (33-35). HKI staff conduct trainings for interviewers, covering survey question content, question-asking techniques, and cultural sensitivity, and for the anthropometry team, standardization exercises with local volunteers. Staff from icddr,b are responsible for training and supervision of blood and stool sampling and processing. Supervisors and trainers conduct quality control in the field and refresher trainings are given when needed. Data are collected via tablets and uploaded after each interview or at the end of the day. Datasets are examined for quality and adjustments to the questionnaires or refresher trainings with the data collectors are done as needed.

Data management

For peer review only

Questionnaires have been programmed to prevent common data collection errors. Data are downloaded and compiled on a regular basis, and data quality checks are conducted. Data concerns are immediately communicated to the data collectors or research team for resolution. All quantitative and qualitative data are stored on password-protected servers and computers. Only deidentified data are shared with collaborators for analysis.

Statistical analysis plan

The main analysis will be to compare the mean length-for-age z-score in children, aged 0-35 months at the start of the endline survey, between intervention and control arms. This will be done using mixed effects linear regression, i.e. modelling between-cluster variation as a random effect in a full probability model. This method will be statistically more efficient than analysing results at the cluster level and also allows for analysis of individual-level covariates (36). In order to reduce between-cluster variation and thus increase power and precision of the study, we will adjust for baseline values of the main outcomes of interest (e.g. mean length-for-age at cluster level) as well as potentially other relevant predictors. Secondary and other outcomes that are normally distributed will be analysed in the same way. Logarithmic transformation will be used for continuous variables with a skewed distribution (e.g. agricultural yields). Binary outcomes (e.g. health service use) will be analysed with mixed effects logistic regression models. Missing data on outcome measurements, including loss to follow-up, will be compared between intervention and control arms with respect to baseline covariates to assess the potential for selection bias.

Patients and public involvement

Participants were not involved in the study design or in the setting of research objectives or outcomes. Intervention implementation is adapted to participant concerns and constraints. Key stakeholders will be involved in dissemination of research findings.

For peer review only

Ethics and dissemination

Ethical approvals

The full FAARM trial protocol was positively reviewed by the University of Heidelberg in Germany (Reference: S-121/2014). The trial protocol without biological sample collection was approved by the James P. Grant School of Public Health, BRAC University (IRB Registration: IRB00003441) and the blood sampling by the Bangladesh Medical Research Council (Reference: BMRC/NREC/2013-2016/844). The food hygiene intervention and evaluation protocol including stool sampling and blood sampling at endline was approved by the University of Heidelberg (Reference: S-606/2017) and the International Centre for Diarrhoeal Disease Research, Bangladesh (Reference: PR-17126). Any protocol changes are reported to the respective ethical boards for approval. The FAARM trial is registered at Clinicaltrials.gov (ID: NCT02505711).

Consent

All enrolled FAARM participants provide informed, written consent by signature, or thumbprint if illiterate, for themselves and for their children, if applicable. All participants receive an information sheet with contact information of the local FAARM manager for any questions or concerns.

Risks and benefits

The risks from participation (time expenditure over four years, anthropometric measurement and biological sampling) are minimal when compared to the potential knowledge gained from this study. There are some benefits for all participants, including referral for serious conditions when detected during surveys, early pregnancy identification (through distribution

For peer review only

For peer review only - http://bmjopen.bmj.com/site/about/guidelines.xhtml

of pregnancy tests), and initial breastfeeding counselling. Participants in the intervention arm additionally benefit from intervention activities as they receive assets, training and support to adopt promoted behaviours.

All participants are informed about their anthropometric measurements in cases of low BMI or child growth, and haemoglobin values when anemic. The results of the trial will be disseminated to all research and implementation partners, published in peer-reviewed journals, and presented at national and international conferences and events. Programme- and policy-related findings will be distributed through the HKI network and other agricultural and nutrition fora.

Project status

As of April 2019, participant enrolment, baseline survey and intervention activities have been completed. The monitoring, and surveillance activities are ongoing and the endline survey is planned for late 2019.

Discussion

To our knowledge, this is the first randomized controlled trial to assess the impact of a complex, nutrition-sensitive agricultural programme on children's nutritional status that enrols women prior to pregnancy, thus capturing the full first 1,000 days of child development. It is particularly relevant and well-placed in rural Bangladesh where stunting and micronutrient deficiencies are common. We will be able to assess not only *whether* the intervention has impacted each outcome but also *how* this occurs, due to a comprehensive monitoring and surveillance system through which we track programme fidelity and intensity, participation, and outcomes along impact pathways. By assessing participant knowledge and behaviours as well as anthropometric and biological measures, we will be able to examine

For peer review only

changes on a wide range of indicators. In addition, the rotating data collection pattern of the surveillance system will allow the project to map out changes in diet over seasons and between intervention and control households over time. Data on the sources of foods consumed by households will enable tracking of changes in food consumption habits, comparing garden production to market purchases. A limitation of our study is the lack of continuous child growth monitoring and blood sampling during surveillance, due to financial and logistic constraints. Data collection of children will also end at three years of age, which may miss growth or child development impacts which appear later.

We expect the findings from this trial will contribute to national policy discussions regarding the contribution of complex nutrition-sensitive agricultural interventions to improving nutritional status and other outcomes. Furthermore, trial results may be generalizable to other settings where poverty, food insecurity and climate change affect nutrition and health.

Acknowledgements

The authors would like to thank the generous funders that make this research possible. We also thank all researchers who contributed their time and knowledge to this study, in particular Tarique Huda, Shafinaz Sobhan, Philipp Zanger, Nicholas Kyei, Andreas Deckert, Andrea Seiermann, Simone Jacobs, Malabika Sarker, Irmgard Jordan, Katja Kehlenbeck, Sebastian Kern, Hans-Peter Schmidt, Lisa Hefele, Wolf-Peter Schmidt, Jody Harris, Om Prasad Gautam, and Sheela Sinharoy as well as our administrator Elke Braun-van der Hoeven, and Gabriele Stieglbauer for assistance with data management. We furthermore thank HKI for their support in establishing and conducting the FAARM trial, in particular the leadership provided by Zaman Talukder, Meredith Jackson-de Graffenried, Sheela Sinharoy, Amin Uddin and Shirin Afroz at the HKI country office, Nancy Haselow and Ame Stormer at the HKI regional office, and Rolf Klemm and Jennifer Nielsen at HKI headquarters. Most

For peer review only

importantly, the trial would not be possible without the dedicated research and implementation staff working on the ground to carry the project to fruition. We thank the HKI research team led by Abdul Kader, with field leadership from Shobha Tripura and Sailen Akhter and analysis support from Chowdhury Asif, Md. Arafat Rahman, and Masum Ali. We also warmly thank our implementation team from HKI – Zillur Rahman, Basana Marma and Md. Wali-Ul Islam – and the VARD team carrying out the daily work in the villages. Lastly, we want to thank all the women, children and families in Sylhet who generously give their time and their effort to participate in FAARM.

Author contributions

SG conceptualized the study initially, then jointly with JLW. SG, JLW and TMS selected the study site. SG, JLW, ASW, TMS and AAM developed and oversaw surveys, monitoring and surveillance systems. All authors contributed to the development, review, and finalization of this study protocol manuscript.

Funding statement

The FAARM trial is primarily supported by the German Ministry for Education and Research (BMBF) (Award number: 01ER1201). Additional funding for the surveillance programme and other project components comes from the Nutrition Embedding Evaluation Programme (NEEP), funded by the UK Department for International Development (DFID), the Food & Business Applied Research Fund (ARF) of the Dutch Ministry of Foreign Affairs, the Thrasher Research Fund, and the Alexander von Humboldt Foundation. Further support through charitable donations to HKI for the implementation of the HFP programme is provided by the Carrefour social responsibility programme and the Best in Brands social marketing programme. Funding organizations did not have a role in the study design, hiring or training of staff, intervention delivery, data collection, analysis, or interpretation of data.

For peer review only

For peer review only - http://bmjopen.bmj.com/site/about/guidelines.xhtml

Declaration of interests

The authors declare no conflict of interest. The content of this publication is solely the responsibility of the authors.

For peer review only

For peer review only - http://bmjopen.bmj.com/site/about/guidelines.xhtml

References

1. Black RE, Victora CG, Walker SP, Bhutta ZA, Christian P, de Onis M, et al. Maternal and child undernutrition and overweight in low-income and middle-income countries. Lancet. 2013;382(9890):427-51.

2. Ruel M, Hoddinott J. Investing in early childhood nutrition. IFPRI Policy Briefs. Washington DC: International Food Policy Research Institute; 2008.

3. UNICEF. Strategy for improved nutrition of children and women in development countries. New York, USA: UNICEF; 1990.

4. Harper KM, Mutasa M, Prendergast AJ, Humphrey J, Manges AR. Environmental enteric dysfunction pathways and child stunting: A systematic review. PLoS Negl Trop Dis. 2018;12(1):e0006205.

5. Bhutta ZA, Ahmed T, Black RE, Cousens S, Dewey K, Giugliani E, et al. What works? Interventions for maternal and child undernutrition and survival. Lancet. 2008;371(9610):417-40.

6. Masset E, Haddad L, Cornelius A, Isaza-Castro J. Effectiveness of agricultural interventions that aim to improve nutritional status of children: systematic review. BMJ. 2012;344:d8222.

7. Ruel MT, Alderman H. Nutrition-sensitive interventions and programmes: how can they help to accelerate progress in improving maternal and child nutrition? Lancet. 2013;382(9891):536-51.

8. Girard AW, Self JL, McAuliffe C, Olude O. The effects of household food production strategies on the health and nutrition outcomes of women and young children: a systematic review. Paediatr Perinat Epidemiol. 2012;26 Suppl 1:205-22.

9. Ruel MT, Quisumbing AR, Balagamwala M. Nutrition-sensitive agriculture: What have we learned so far? Global Food Security. 2018;17:128-53.

10. Harris J, Thompson S, Sparling T. Leveraging Development Programs: Homestead Food Production. In: Ferranti P, Berry EM, Anderson JR, editors. Encyclopedia of Food Security and Sustainability. Oxford: Elsevier; 2019. p. 396-400.

11. Helen Keller International (HKI), James P. Grant School of Public Health (JPGSPH). State of Food Security and Nutrition in Bangladesh: 2014. Dhaka, Bangladesh: HKI and JPGSPH; 2016.

12. Gottlieb CA, Maenner MJ, Cappa C, Durkin MS. Child disability screening, nutrition, and early learning in 18 countries with low and middle incomes: data from the third round of UNICEF's Multiple Indicator Cluster Survey (2005-06). Lancet. 2009;374(9704):1831-9.

13. Monitoring the Situation of Children and Women, Bangladesh: Multiple Indicator Cluster Survey 2009. Bangladesh Bureau of Statistics, Ministry of Planning and United Nations Children's Fund (UNICEF); 2010.

14. Suan Pheng Kam MLB, Tahmina Latif, M A H Chowdhury, S Ghulam Hussain, Mahbub Ahmed, Anwar Iqbal, L Villano, Mahabub Hossain. Geographical Concentration of Rural Poverty in Bangladesh. Dhaka, Bangladesh: Center for Policy Dialogue; 2004.

15. National Institute of Population Research Training - NIPORT/Bangladesh, Mitra Associates, ICF International. Bangladesh Demographic and Health Survey 2014. Dhaka, Bangladesh: NIPORT, Mitra and Associates, and ICF International; 2016.

16. Dewey KG, Adu-Afarwuah S. Systematic review of the efficacy and effectiveness of complementary feeding interventions in developing countries. Matern Child Nutr. 2008;4 Suppl 1:24-85.

For peer review only - http://bmjopen.bmj.com/site/about/guidelines.xhtml

17. Ivers NM, Halperin IJ, Barnsley J, Grimshaw JM, Shah BR, Tu K, et al. Allocation techniques for balance at baseline in cluster randomized trials: a methodological review. Trials. 2012;13:120.
18. Lorenz E, Gabrysch S. Covariate-constrained randomization routine for achieving baseline balance in cluster-randomized trials. Stata Journal. 2017;17(2):503-10.
19. Helen Keller International. Homestead Food Production [Available from: http://www.hki.org/reducing-malnutrition/homestead-food-production/.
20. Olney DK, Vicheka S, Kro M, Chakriya C, Kroeun H, Hoing LS, et al. Using program impact pathways to understand and improve program delivery, utilization, and potential for impact of Helen Keller International's homestead food production program in Cambodia. Food Nutr Bull. 2013;34(2):169-84.
21. Nielsen JN, Olney DK, Ouedraogo M, Pedehombga A, Rouamba H, Yago-Wienne F. Process evaluation improves delivery of a nutrition-sensitive agriculture programme in Burkina Faso. Matern Child Nutr. 2018;14(3):e12573.
22. Improving diets and nutrition: food-based approaches. Thompson B, Amoroso L, editors. Rome, Italy: CABI International and FAO; 2014.
23. Scaling-up "Biochar-urine nutrient cycling for health" in Bangladesh (BUNCH2Scale) 2017 [cited 2019 January 9]. Available from: http://knowledge4food.net/research-project/bangladesh-bunch2scale/.
24. World Health Organization (WHO). Essential Nutrition Actions: Improving maternal, newborn, infant and young child health and nutrition. Geneva, Switzerland: WHO; 2013.
25. Talukder A, Haselow NJ, Osei AK, Villate E, Reario D, Kroeun H, et al. Homestead food production model contributes to improved household food security and nutrition status of young children and women in poor populations. Field Actions Science Reports. 2000(Special Issue 1).
26. FAO, FHI 360. Minimum Dietary Diversity for Women: A Guide to Measurement. Rome, Italy: FAO; 2016.
27. Cox JL, Holden JM, Sagovsky R. Detection of postnatal depression. Development of the 10-item Edinburgh Postnatal Depression Scale. Br J Psychiatry. 1987;150:782-6.
28. Gausia K, Hamadani JD, Islam MM, Ali M, Algin S, Yunus M, et al. Bangla translation, adaptation and piloting of Edinburgh Postnatal Depression Scale. Bangladesh Med Res Counc Bull. 2007;33(3):81-7.
29. World Health Organization (WHO). Indicators for assessing infant and young child feeding practices: Part 2 Measurement. Geneva, Switzerland: WHO; 2010.
30. WHO Motor Development Study: windows of achievement for six gross motor development milestones. Acta Paediatr Suppl. 2006;450:86-95.
31. World Health Organization (WHO). WHO child growth standards : length/height-for-age, weight-for-age, weight-for-length, weight-for-height and body mass index-for-age : methods and development. Geneva, Switzerland: WHO; 2006.
32. WHO Anthro for personal computers: Software for assessing growth and development of the world's children. 3.2.2 ed. Geneva: WHO; 2010.
33. World Health Organization (WHO). WHO guidelines on drawing blood: best practices in phlebotomy. Geneva, Switzerland: WHO; 2010.
34. Cogill B. Anthropometric Indicators Measurement Guide. Washington, D.C.: Food and Nutrition Technical Assistance Project, Academy for Educational Development; 2003.

For peer review only

35. Cashin K, Oot L. Guide to anthropometry: a practical tool for program planners, managers, and implementers. Washington, D.C.: Food and Nutrition Technical Assistance III Project (FANTA) / FHI 360; 2018.
36. Hayes RJ, Moulton LH. Cluster Randomised Trials: Chapman & Hall/CRC; 2009.

For peer review only

Table 1: FAARM data sources and data collection methods

	**Data source**	**Data collection device**	**Responsible team**	**Population**	**Frequency**
Survey	Baseline	Tablet	Survey team	Intervention and control group	Once in 2015
Programme monitoring	Intervention activity records	Paper, then Smartphone	Implementation team	Intervention group	Rolling: During or after programme activities
Programme monitoring	Output monitoring	Smartphone	Implementation team	Intervention group	Annually on a rolling basis
Surveillance	Routine assessment	Tablet	Surveillance team	Intervention and control group	Every 2 months
Surveillance	Food consumption	Tablet	Surveillance team	Sub-sample: Intervention and control group	Annually on a rolling basis
Surveillance	Birth registry	Tablet	Surveillance team	At birth: Intervention and control group	Within 72 hours of childbirth
Survey	Endline	Tablet	Survey team	Intervention and control group	Once in 2019

For peer review only

Figure titles and legends

Figure 1: Flowchart of FAARM participant selection and randomization
FAARM: Food and Agricultural Approaches to Reducing Malnutrition

Figure 2: FAARM study site

The large map shows the FAARM study site with 96 clusters in 13 unions of 2 sub-districts (Nabiganj and Baniachong) and the small map on the top shows its location within Habiganj District and Sylhet Division in Bangladesh.
FAARM: Food and Agricultural Approaches to Reducing Malnutrition

Figure 3: Activity timeline for FAARM trial in Sylhet, Bangladesh
FAARM: Food and Agricultural Approaches to Reducing Malnutrition

Figure 4: FAARM conceptual framework
The blue boxes represent components of Homestead Food Production intervention trainings and counselling sessions. These will influence the outcomes listed (the green boxes) with women's empowerment interacting with all aspects. Better outcomes then lead to improved impacts including child development and growth.
FAARM: Food and Agricultural Approaches to Reducing Malnutrition

For peer review only

For peer review only - http://bmjopen.bmj.com/site/about/guidelines.xhtml

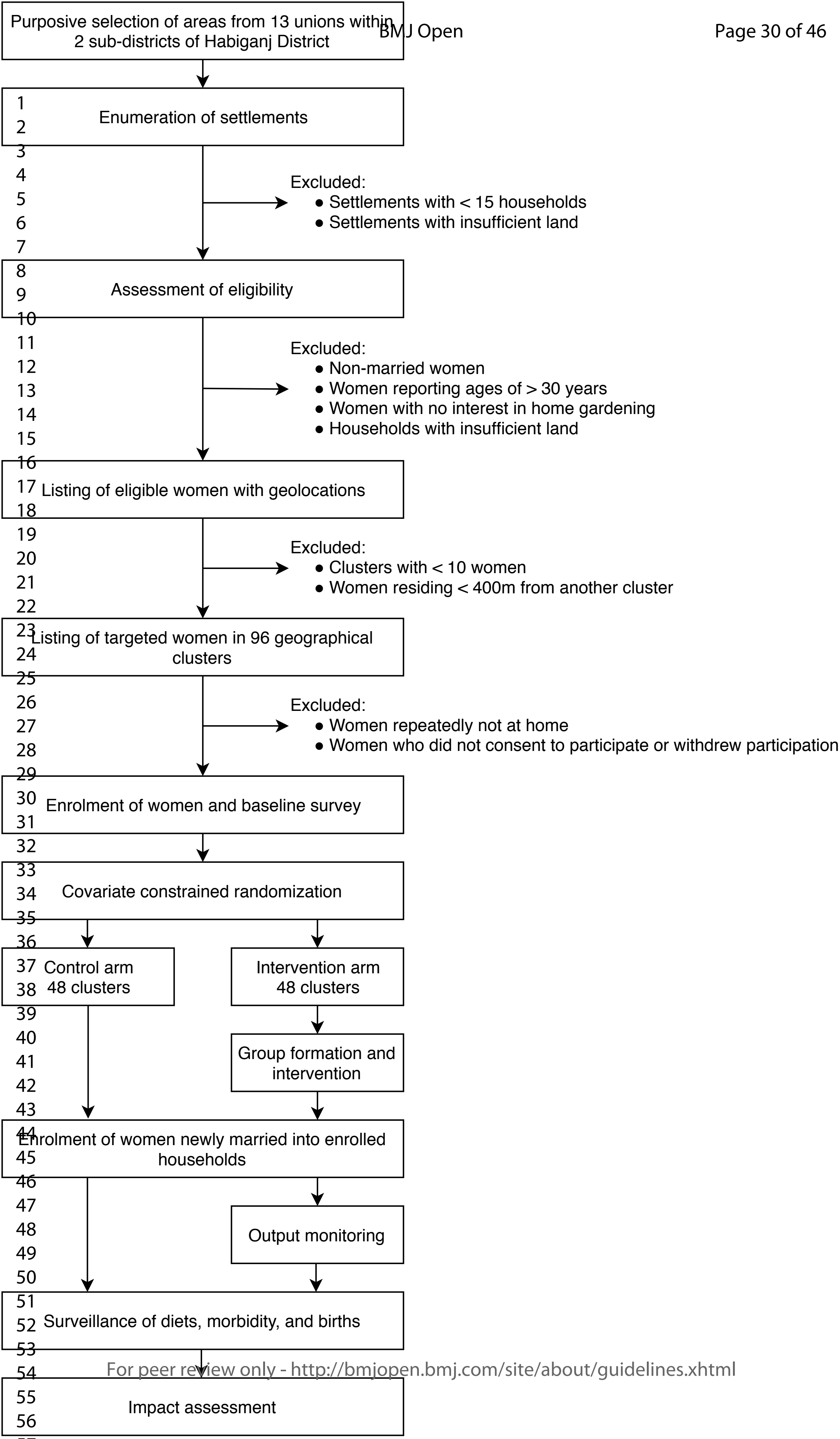
Purposive selection of areas from 13 unions within 2 sub-districts of Habiganj District
Enumeration of settlements
Excluded:
● Settlements with < 15 households
● Settlements with insufficient land
Assessment of eligibility
Excluded:
● Non-married women
● Women reporting ages of > 30 years
● Women with no interest in home gardening
● Households with insufficient land
Listing of eligible women with geolocations
Excluded:
● Clusters with < 10 women
● Women residing < 400m from another cluster
Listing of targeted women in 96 geographical clusters
Excluded:
● Women repeatedly not at home
● Women who did not consent to participate or withdrew participation
Enrolment of women and baseline survey
Covariate constrained randomization
Control arm 48 clusters
Intervention arm 48 clusters
Group formation and intervention
Enrolment of women newly married into enrolled households
Output monitoring
Surveillance of diets, morbidity, and births
Impact assessment

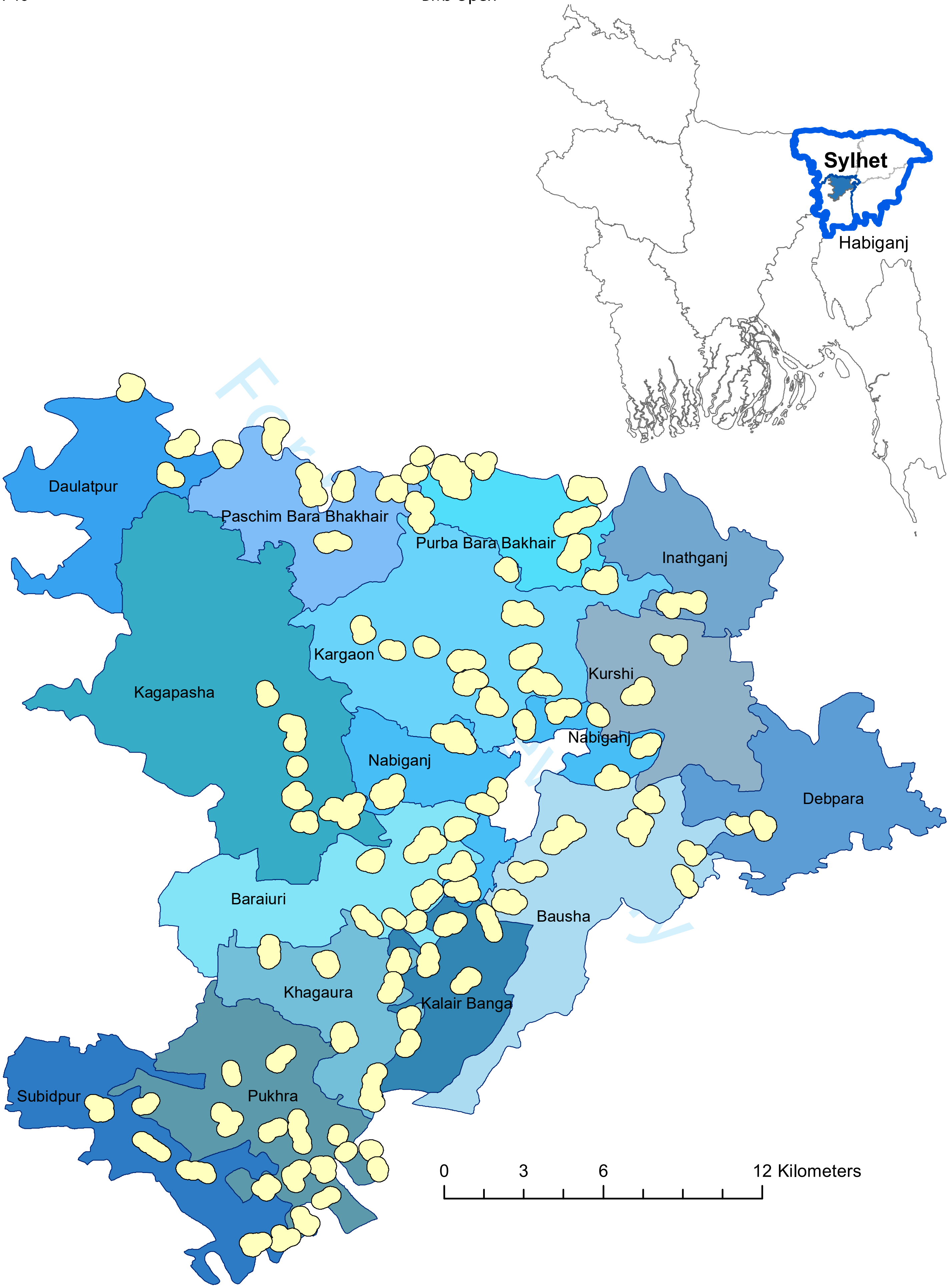
Sylhet
Habiganj
Daulatpur
Paschim Bara Bhakhair
Purba Bara Bakhair
Inathganj
Kargaon
Kagapasha
Kurshi
Nabiganj
Nabiganj
Debpara
Baraiuri
Bausha
Khagaura
Kalair Banga
Subidpur
Pukhra
0
3
6
12 Kilometers

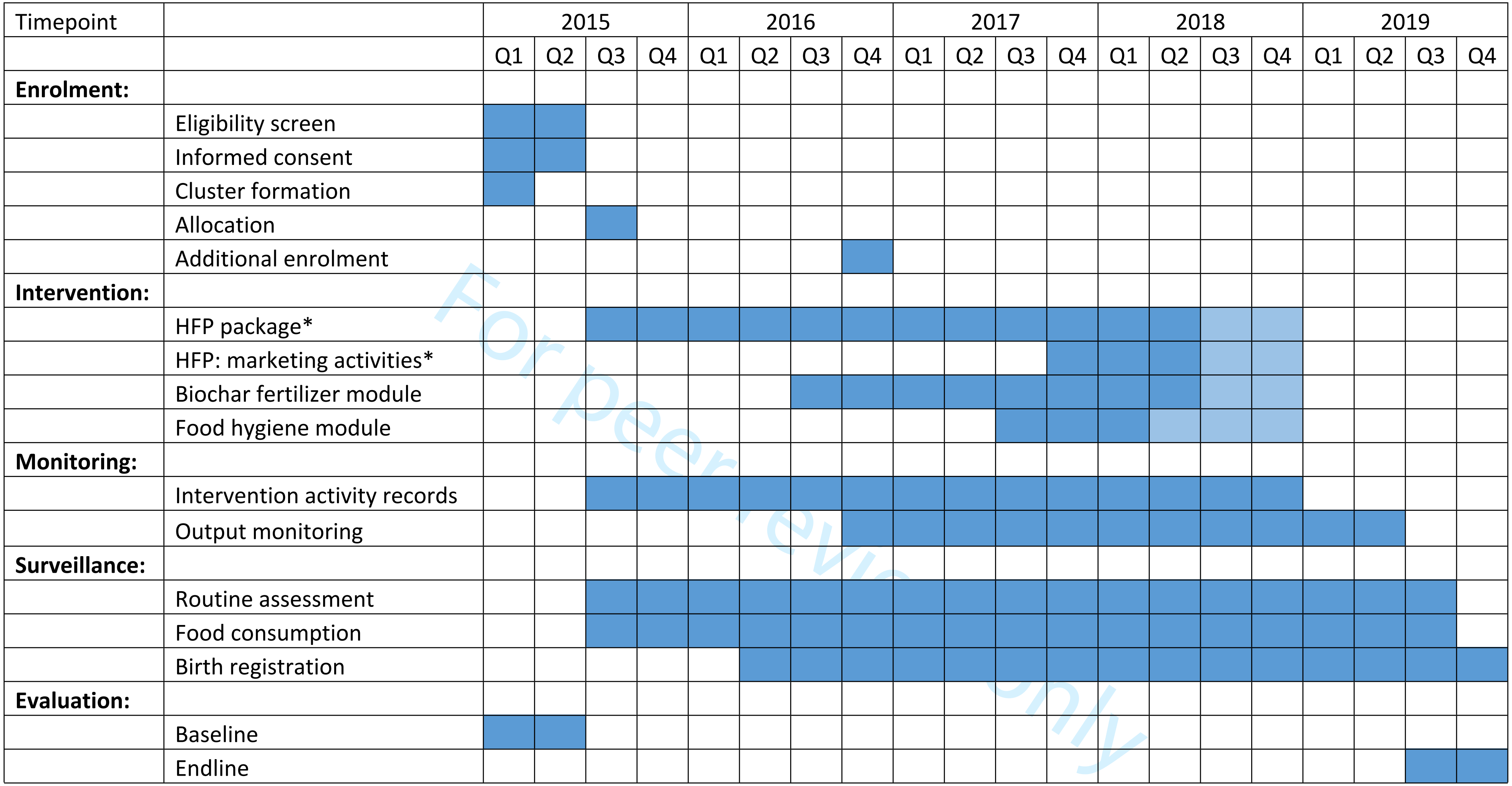

* Lighter blue denotes a gradual lowering of the intervention intensity, with only household counselling sessions.

For peer review only

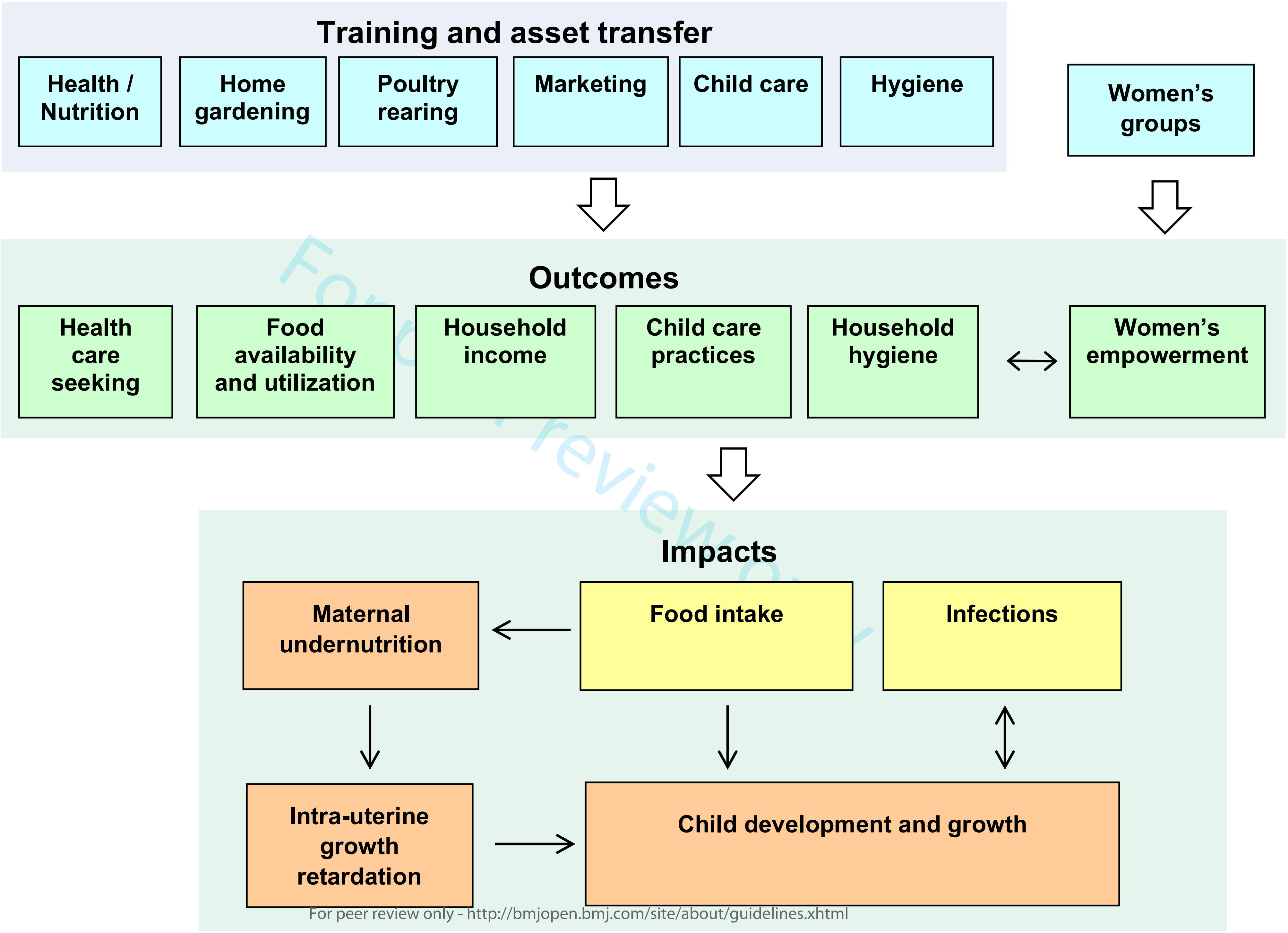

For peer review only - http://bmjopen.bmj.com/site/about/guidelines.xhtml

Supplementary File 1: Food Hygiene to reduce Environmental Enteric Dysfunction (FHEED)

In addition to promoting homestead gardens and nutrition education, a special behavior change intervention has been designed to strengthen the food hygiene component in FAARM. This food hygiene intervention uses a behavior-centered design by employing emotional drivers (such as pride, disgust and nurture) as well as engaging group activities (including games and competitions) and repeated individual household visits to improve common food hygiene practices among study participants.

The intervention focuses on the improvement of six identified key optimal feeding and food hygiene behaviors: exclusive breastfeeding up to 6 months, improved diversity and nutritional value of food (to achieve integration with the Homestead Food Production messages), cleanliness of serving utensils, proper hand-washing before feeding/eating, adequate food storage, and thorough reheating of stored food.

The food hygiene intervention is delivered to groups of 5 to 25 women by eight food hygiene promoters through two core components: group events, which facilitate exchange and interactive discussions, and individual household visits, which provide the opportunity of one-on-one counselling on key behaviors and individual questions. In total, the food hygiene promoters conducted four group events per women group and four household visits per woman over an eight-month period (July 2017 to February 2018).

The FHEED study was added as sub-study to the FAARM trial to evaluate the impact of food hygiene on the development of intestinal infections and Environmental Enteric Dysfunction (EED), as a second key pathway to child growth alongside nutrition. It specifically studies factors on the pathway from food hygiene to EED, including women´s food hygiene behaviour (measured through structured observation), complementary food contamination (log-transformed counts of total coliforms and *E.coli* in food samples), intestinal pathogen load (pathogen-specific qRT-PCR), diarrheal episodes and EED biomarkers (fecal myeloperoxidase, fecal α1-antitrypsin fecal neopterin, serum C-reactive protein, serum α1-acid glycoprotein). The FHEED study will assess these parameters in children aged 0-18 months in two cross-sectional surveys (in 2018 and 2019). In addition, children born in 2018 after the end of the food hygiene intervention will be enrolled in a cohort and sampled every two months over a 12-month period.

For peer review only

Supplementary File 2:

Blood samples:

During the baseline survey in 2015, 300 µL of whole capillary blood was collected (in Microvette™ tubes) from enrolled women and children. Blood samples were then transported to the field laboratory maintaining a cold chain (+4°C to +8°C at all times) and stored in a refrigerator at 4°C over night. The following morning, samples were centrifuged in an Eppendorf Mini-Spin centrifuge for 10 minutes at 13,400 rounds per minute. The supernatant was pipetted into 50 µL aliquots (approximately 2-3 per sample). Serum and red blood cells were then stored in a -20°C freezer on site. Samples that were transported to Germany on dry ice were again stored in a -20°C freezer before analysis.

At the endline survey in 2019, we will collect venous blood from enrolled woman (in total 8 mL) and children (in total 5 mL) into the Vacutainer™ collection system EDTA and serum tubes, through puncture of a cubital vein. Blood samples will be transported to the field laboratory maintaining a cold chain (+4°C to +8°C at all times) and stored in a refrigerator at 4°C over night. The following morning, EDTA samples will be used for complete blood count analysis. Serum and red blood cells will be stored in a -20°C freezer on site. Samples will then be transported to Germany on dry ice and again stored in a -20°C freezer before analysis.

Faecal samples:

Stool samples are collected during a cross-sectional survey in all children aged 0-18 months in 2018 and at study endline in 2019. In a subset (150 households), we additionally collect maternal stool samples. Stool collection sets (stool collection tubes, a sterile stick for stool collection and gloves) are handed to the mothers with instructions one day prior to collection. We collect one native stool sample and one stool sample preserved in 7 mL RNA-later for nucleic acid extraction. Stool samples are aliquoted and stored in a -20°C freezer on site until shipment to Germany on dry ice for further analysis and long-term storage at -80°C.

For peer review only

Table S1: Surveillance indicators collected during routine assessment of the FAARM trial in Sylhet, Bangladesh

Category	*Indicator*	*Definition*	*Frequency of collection*
Pregnancy & birth identification	Last menstrual period	First day of last menstrual period	Every two months
	Pregnancy status	Identification of new, continued, or loss of pregnancy	Every two months
	Newborn identification	Newborn date of birth record	Every two months
Prenatal and postnatal health	Intended pregnancy	If the woman had intended to get pregnant at this time	Once pregnancy is identified
	ANC attendance	Antenatal care-seeking and home visits by health workers	Every two months during pregnancy
	ANC services	Woman's weight taken, blood pressure measured, urine sample taken, blood sample taken, ultrasonography done, child position checked, eye check for anemia, tetanus toxoid injection given	Every two months during pregnancy
	Iron and folic acid consumption	Intake of iron and folic acid in the previous 7 days	Every two months during pregnancy
	Resting time	Hours of rest and sleep in the previous 24 hours	Every two months during pregnancy
	Night blindness	Difficulty seeing at dusk or night	Every two months during pregnancy
	Pregnancy complications	Reporting of twins, severe headaches, high blood pressure, etc.	Once per pregnancy (≥ 6 months of gestation)
	Past complications	Reporting of past pregnancy complications	Asked once during first reported pregnancy

For peer review only - http://bmjopen.bmj.com/site/about/guidelines.xhtml

	Birth delivery plan	Intended place of delivery	Once per pregnancy (≥ 6 months), confirmed in follow-up visits
	Depression	Edinburgh Postnatal Depression Scale (adapted to Bangladesh)[1]	Once during pregnancy (≥ 6 months gestation), once when child is born and once when child is 6-12 months of age
Women's nutrition	Dietary diversity	Information on 21 food groups[2 3]	Every 6 months for all women<br>Every 2 months during pregnancy
	Weight	Weight of women in kg using Tanita scales	Every 12 months for all women<br>Every 2 months during pregnancy
Children's nutrition	IYCF	WHO IYCF indicators[4]	
	Dietary diversity	Information on 21 food groups[2 3]	
	Weight	Weight of children in kg using Tanita scales	When child is approximately 18 months of age
Child morbidity	Diarrhea	7-day period prevalence; 2-day point prevalence	Every two months
	Respiratory distress	7-day period prevalence	Every two months
	Sick child feeding & healthcare seeking	If child was taken to health care facility, child feeding behaviors	Every two months, when child was reported as ill
Child development	Motor Milestones	Child performance of developmental motor milestones according to age[5]	When child reaches appropriate age for milestone
	Language	Child language questionnaire adapted from Frongillo et al, 2017[6]	Every two months when child is 5-11 months of age
Agriculture	Crop diversity	Listing of vegetables, fruits, and spices grown in the past growing season.	

ANC: antenatal care; **IYCF**: Infant and young child feeding;

For peer review only - http://bmjopen.bmj.com/site/about/guidelines.xhtml

References:

1. Gausia K, Hamadani JD, Islam MM, et al. Bangla translation, adaptation and piloting of Edinburgh Postnatal Depression Scale. *Bangladesh Medical Research Council Bulletin* 2007;33(3):81-7. [published Online First: 2008/09/12]
2. Wiesmann D, Arimond M, Loechi C. Dietary diversity as a measure of the micronutrient adequacy of women's diets: results from rural Mozambique site. Washington, DC: Food and Nutrition Technical Assistance II Project, FHI 360, 2009.
3. FAO, FHI 360. Minimum Dietary Diversity for Women: A Guide to Measurement. Rome, Italy: FAO 2016.
4. World Health Organization (WHO). Indicators for assessing infant and young child feeding practices: Part 2 Measurement. Geneva, Switzerland: WHO 2010.
5. WHO Motor Development Study: windows of achievement for six gross motor development milestones. *Acta Paediatrica (Oslo, Norway: 1992) Supplement* 2006;450:86-95. [published Online First: 2006/07/05]
6. Frongillo EA, Nguyen PH, Saha KK, et al. Large-Scale Behavior-Change Initiative for Infant and Young Child Feeding Advanced Language and Motor Development in a Cluster-Randomized Program Evaluation in Bangladesh. *The Journal of Nutrition* 2017;147(2):256-63. doi: 10.3945/jn.116.240861 [published Online First: 2016/12/30]

For peer review only

Table S2: FAARM trial measures of anthropometry, biomarkers, and food contaminants

Category	*Indicator*	*Time of measurement*	*Equipment / Laboratory test*
Anthropometry	Woman weight	Baseline;<br>Routine Assessment (once per year, every 2 months during pregnancy);<br>Endline	Baseline/Endline: SECA 874 digital floor scale<br>RA: Tanita weight scales
	Woman height	Baseline;<br>Endline[a]	SECA 217 stadiometer
	Woman MUAC	Routine Assessment<br>(every 2 months during pregnancy)	SECA 212 measuring tape
	Child weight	Baseline;<br>Routine Assessment<br>(around 18 months);<br>Endline	Baseline/Endline: SECA 874 digital floor scale<br>RA: SECA 334
	Child length	Baseline;<br>Birth Registration (newborn length);<br>Endline	Baseline/Endline: SECA 416 digital baby scale<br>BR: SECA 232 measuring rod (until March 2017); locally made length board (from April 2017)
	Child MUAC	Birth Registration	SECA 212
	Child head circumference	Birth Registration	SECA 212
Biomarkers	Hemoglobin	Baseline;<br>Endline	Hemocue 201+<br>Sysmex XP-100 Hematology Analyzer
	Serum ferritin[b]	Baseline;<br>Endline	Sandwich ELISA[1]
	Serum transferrin receptor[b]	Baseline;<br>Endline	Sandwich ELISA[1]

For peer review only

	Retinol-binding protein[b]	Baseline; Endline	Sandwich ELISA[1]
	C-reactive protein[b]	Baseline; Endline	Sandwich ELISA[1]
	Serum α-1-acid glycoprotein[b]	Baseline; Endline	Sandwich ELISA[1]
	Serum zinc[b]	Endline	Colorimetric assay
	Feacal myeloperoxidase[b]	2018 (Food hygiene evaluation); Endline	Sandwich ELISA[1]
	Feacal α-1-antitrypsin[b]	2018 (Food hygiene evaluation); Endline	Sandwich ELISA[1]
	Feacal neopterin[b]	2018 (Food hygiene evaluation); Endline	Sandwich ELISA[1]
Food contaminants	Coliforms[b]	2018 (Food hygiene evaluation)	Pour plating on Tryptone Bile X-Glucuronide (TBX) Agar
	Escherichia coli[b]	2018 (Food hygiene evaluation)	Pour plating on Tryptone Bile X-Glucuronide (TBX) Agar

[a] For those women who were not measured at baseline. [b] These measurements will be conducted in a sub-sample of the population.
MUAC: mid-upper arm circumference

1. Erhardt JG, Estes JE, Pfeiffer CM, et al. Combined measurement of ferritin, soluble transferrin receptor, retinol binding protein, and C-reactive protein by an inexpensive, sensitive, and simple sandwich enzyme-linked immunosorbent assay technique. *The Journal of Nutrition* 2004;134(11):3127-32. doi: 10.1093/jn/134.11.3127

For peer review only

STANDARD PROTOCOL ITEMS: RECOMMENDATIONS FOR INTERVENTIONAL TRIALS

SPIRIT 2013 Checklist: Recommended items to address in a clinical trial protocol and related documents*

Section/item	Item No	Description	Addressed on page number
**Administrative information**			
Title	1	Descriptive title identifying the study design, population, interventions, and, if applicable, trial acronym	__1___________
Trial registration	2a	Trial identifier and registry name. If not yet registered, name of intended registry	___1__________
	2b	All items from the World Health Organization Trial Registration Data Set	_Throughout____
Protocol version	3	Date and version identifier	____n/a________
Funding	4	Sources and types of financial, material, and other support	____23_________
Roles and responsibilities	5a	Names, affiliations, and roles of protocol contributors	____1, 23_______
	5b	Name and contact information for the trial sponsor	___n/a______
	5c	Role of study sponsor and funders, if any, in study design; collection, management, analysis, and interpretation of data; writing of the report; and the decision to submit the report for publication, including whether they will have ultimate authority over any of these activities	____23________
	5d	Composition, roles, and responsibilities of the coordinating centre, steering committee, endpoint adjudication committee, data management team, and other individuals or groups overseeing the trial, if applicable (see Item 21a for data monitoring committee)	____n/a________

For peer review only - http://bmjopen.bmj.com/site/about/guidelines.xhtml

Introduction

Background and rationale	6a	Description of research question and justification for undertaking the trial, including summary of relevant studies (published and unpublished) examining benefits and harms for each intervention	______4-5_____
	6b	Explanation for choice of comparators	___5, 9_______
Objectives	7	Specific objectives or hypotheses	___5_________
Trial design	8	Description of trial design including type of trial (eg, parallel group, crossover, factorial, single group), allocation ratio, and framework (eg, superiority, equivalence, noninferiority, exploratory)	__6__________

Methods: Participants, interventions, and outcomes

Study setting	9	Description of study settings (eg, community clinic, academic hospital) and list of countries where data will be collected. Reference to where list of study sites can be obtained	__6__________
Eligibility criteria	10	Inclusion and exclusion criteria for participants. If applicable, eligibility criteria for study centres and individuals who will perform the interventions (eg, surgeons, psychotherapists)	___7-8_______
Interventions	11a	Interventions for each group with sufficient detail to allow replication, including how and when they will be administered	___9-12______
	11b	Criteria for discontinuing or modifying allocated interventions for a given trial participant (eg, drug dose change in response to harms, participant request, or improving/worsening disease)	___n/a_______
	11c	Strategies to improve adherence to intervention protocols, and any procedures for monitoring adherence (eg, drug tablet return, laboratory tests)	___13-14_____
	11d	Relevant concomitant care and interventions that are permitted or prohibited during the trial	____n/a______
Outcomes	12	Primary, secondary, and other outcomes, including the specific measurement variable (eg, systolic blood pressure), analysis metric (eg, change from baseline, final value, time to event), method of aggregation (eg, median, proportion), and time point for each outcome. Explanation of the clinical relevance of chosen efficacy and harm outcomes is strongly recommended	___16-18_____
Participant timeline	13	Time schedule of enrolment, interventions (including any run-ins and washouts), assessments, and visits for participants. A schematic diagram is highly recommended (see Figure)	_Figure 3_____

For peer review only - http://bmjopen.bmj.com/site/about/guidelines.xhtml

Sample size	14	Estimated number of participants needed to achieve study objectives and how it was determined, including clinical and statistical assumptions supporting any sample size calculations	__7___________
Recruitment	15	Strategies for achieving adequate participant enrolment to reach target sample size	_7-8_________

Methods: Assignment of interventions (for controlled trials)

Allocation:

Sequence generation	16a	Method of generating the allocation sequence (eg, computer-generated random numbers), and list of any factors for stratification. To reduce predictability of a random sequence, details of any planned restriction (eg, blocking) should be provided in a separate document that is unavailable to those who enrol participants or assign interventions	__8-9________
Allocation concealment mechanism	16b	Mechanism of implementing the allocation sequence (eg, central telephone; sequentially numbered, opaque, sealed envelopes), describing any steps to conceal the sequence until interventions are assigned	__9________
Implementation	16c	Who will generate the allocation sequence, who will enrol participants, and who will assign participants to interventions	_7-9_________
Blinding (masking)	17a	Who will be blinded after assignment to interventions (eg, trial participants, care providers, outcome assessors, data analysts), and how	_n/a_________
	17b	If blinded, circumstances under which unblinding is permissible, and procedure for revealing a participant's allocated intervention during the trial	_n/a_________

Methods: Data collection, management, and analysis

Data collection methods	18a	Plans for assessment and collection of outcome, baseline, and other trial data, including any related processes to promote data quality (eg, duplicate measurements, training of assessors) and a description of study instruments (eg, questionnaires, laboratory tests) along with their reliability and validity, if known. Reference to where data collection forms can be found, if not in the protocol	__13-18______
	18b	Plans to promote participant retention and complete follow-up, including list of any outcome data to be collected for participants who discontinue or deviate from intervention protocols	__19_____

Data management	19	Plans for data entry, coding, security, and storage, including any related processes to promote data quality (eg, double data entry; range checks for data values). Reference to where details of data management procedures can be found, if not in the protocol	__18-19______
Statistical methods	20a	Statistical methods for analysing primary and secondary outcomes. Reference to where other details of the statistical analysis plan can be found, if not in the protocol	__19______
	20b	Methods for any additional analyses (eg, subgroup and adjusted analyses)	__19______
	20c	Definition of analysis population relating to protocol non-adherence (eg, as randomised analysis), and any statistical methods to handle missing data (eg, multiple imputation)	__19______
**Methods: Monitoring**			
Data monitoring	21a	Composition of data monitoring committee (DMC); summary of its role and reporting structure; statement of whether it is independent from the sponsor and competing interests; and reference to where further details about its charter can be found, if not in the protocol. Alternatively, an explanation of why a DMC is not needed. *The FAARM trial is a behavioural trial and has only minimal risk. Therefore, a data monitoring committee was not required (Lin et al, 2014).*	________see explanation___
	21b	Description of any interim analyses and stopping guidelines, including who will have access to these interim results and make the final decision to terminate the trial	_n/a_________
Harms	22	Plans for collecting, assessing, reporting, and managing solicited and spontaneously reported adverse events and other unintended effects of trial interventions or trial conduct	__13-18, 20___
Auditing	23	Frequency and procedures for auditing trial conduct, if any, and whether the process will be independent from investigators and the sponsor	___n/a______
**Ethics and dissemination**			
Research ethics approval	24	Plans for seeking research ethics committee/institutional review board (REC/IRB) approval	_____19-20____
Protocol amendments	25	Plans for communicating important protocol modifications (eg, changes to eligibility criteria, outcomes, analyses) to relevant parties (eg, investigators, REC/IRBs, trial participants, trial registries, journals, regulators)	_____20______

For peer review only - http://bmjopen.bmj.com/site/about/guidelines.xhtml

Consent or assent	26a	Who will obtain informed consent or assent from potential trial participants or authorised surrogates, and how (see Item 32)	_____8, 20______
	26b	Additional consent provisions for collection and use of participant data and biological specimens in ancillary studies, if applicable	____8, 20_____
Confidentiality	27	How personal information about potential and enrolled participants will be collected, shared, and maintained in order to protect confidentiality before, during, and after the trial	____19_______
Declaration of interests	28	Financial and other competing interests for principal investigators for the overall trial and each study site	____23_______
Access to data	29	Statement of who will have access to the final trial dataset, and disclosure of contractual agreements that limit such access for investigators	___23________
Ancillary and post-trial care	30	Provisions, if any, for ancillary and post-trial care, and for compensation to those who suffer harm from trial participation	__n/a________
Dissemination policy	31a	Plans for investigators and sponsor to communicate trial results to participants, healthcare professionals, the public, and other relevant groups (eg, via publication, reporting in results databases, or other data sharing arrangements), including any publication restrictions	___3, 22_____
	31b	Authorship eligibility guidelines and any intended use of professional writers	_followed Vancouver criteria____
	31c	Plans, if any, for granting public access to the full protocol, participant-level dataset, and statistical code	__n/a_________
**Appendices**			
Informed consent materials	32	Model consent form and other related documentation given to participants and authorised surrogates	___n/a_______
Biological specimens	33	Plans for collection, laboratory evaluation, and storage of biological specimens for genetic or molecular analysis in the current trial and for future use in ancillary studies, if applicable	Table S2; Supp File 2

For peer review only - http://bmjopen.bmj.com/site/about/guidelines.xhtml

*It is strongly recommended that this checklist be read in conjunction with the SPIRIT 2013 Explanation & Elaboration for important clarification on the items. Amendments to the protocol should be tracked and dated. The SPIRIT checklist is copyrighted by the SPIRIT Group under the Creative Commons "Attribution-NonCommercial-NoDerivs 3.0 Unported" license.

For peer review only

Food and Agricultural Approaches to Reducing Malnutrition (FAARM): Protocol for a cluster-randomised controlled trial to evaluate the impact of a Homestead Food Production programme on undernutrition in rural Bangladesh

Journal:	*BMJ Open*
Manuscript ID	bmjopen-2019-031037.R1
Article Type:	Protocol
Date Submitted by the Author:	13-May-2019
Complete List of Authors:	Wendt, Amanda; University of Heidelberg, Heidelberg Institute of Global Health<br>Sparling, Thalia M; University of Heidelberg, Heidelberg Institute of Global Health; Tufts University, Friedman School of Nutrition Science and Policy<br>Waid, Jillian; University of Heidelberg, Heidelberg Institute of Global Health; Hellen Keller International<br>Mueller, Anna; University of Heidelberg, Heidelberg Institute of Global Health<br>Gabrysch, Sabine; University of Heidelberg, Heidelberg Institute of Global Health
**Primary Subject Heading**:	Global health
Secondary Subject Heading:	Epidemiology, Public health, Nutrition and metabolism
Keywords:	Nutrition < TROPICAL MEDICINE, Community child health < PAEDIATRICS, PUBLIC HEALTH, EPIDEMIOLOGY

Title:

Food and Agricultural Approaches to Reducing Malnutrition (FAARM): Protocol for a cluster-randomised controlled trial to evaluate the impact of a Homestead Food Production programme on undernutrition in rural Bangladesh

Authors: Amanda S. Wendt, Thalia M. Sparling, Jillian L. Waid, Anna A. Mueller, Sabine Gabrysch

Addresses / affiliations:

Heidelberg Institute of Global Health, Heidelberg University, 69120 Heidelberg, Germany

Amanda S. Wendt
Postdoctoral researcher

Thalia M. Sparling
Postdoctoral researcher

Jillian L. Waid
Doctoral student

Anna A. Müller
Postdoctoral researcher

Sabine Gabrysch
Associate professor

Helen Keller International, Bangladesh Country Office, Dhaka 1212, Bangladesh

Jillian L. Waid
Director, Research, Learning and Evaluation

Friedman School of Nutrition Science and Policy, Tufts University, Boston 02111, Massachusetts, USA

Thalia M. Sparling
Postdoctoral researcher

Correspondence to: Sabine Gabrysch, sabine.gabrysch@uni-heidelberg.de

Word count: 5,443

Abbreviations:

BMI: Body mass index
FAARM: Food and Agricultural Approaches to Reducing Malnutrition
HFP: Homestead Food Production
HKI: Helen Keller International

For peer review only

NGO: Non-governmental organization
VARD: Voluntary Association for Rural Development
WHO: World Health Organization
UNICEF: United Nations Children’s Fund

For peer review only

For peer review only - http://bmjopen.bmj.com/site/about/guidelines.xhtml

Abstract

Introduction: Chronic undernutrition affects over 150 million children worldwide and has serious consequences. The causes are complex and include insufficient dietary diversity and poor hygiene practices. Systematic reviews of nutrition-sensitive agricultural interventions concluded that while these hold promise, there is insufficient evidence for their impact on child growth. The Food and Agricultural Approaches to Reducing Malnutrition (FAARM) project is a 1:1 cluster-randomised trial aiming to evaluate the impact of a Homestead Food Production (HFP) programme implemented by Helen Keller International (HKI) on women's and children's undernutrition.

Methods and analysis: The HFP intervention comprises training of women's groups and asset distribution to support year-round home gardening, poultry rearing, and improved nutrition and hygiene practices. Formal trainings are supplemented by behaviour change communication during household visits, and facilitated links between producer groups and market actors. The FAARM trial will examine if and how this complex intervention reduces undernutrition. In 2015, FAARM enrolled married women and their children (0-3 years) in 96 rural settlements of Habiganj district in Sylhet division, Bangladesh. Covariate-constrained randomization was used to assign 48 settlements to receive a three-year HFP intervention, with the other 48 acting as controls, targeting over 2700 women. To study impact pathways, a surveillance system collects data on all participants every two months. In late 2019, children 0-3 years of age (born during the intervention period) will be surveyed, thus capturing impact during the critical first 1,000 days of life. Children's length-for-age z-scores will be compared between intervention and control arms using mixed effects linear regression. Secondary outcomes include women's and children's micronutrient status, dietary intake, dietary diversity, and other indicators of child growth, development, and morbidity.

For peer review only - http://bmjopen.bmj.com/site/about/guidelines.xhtml

Ethics and dissemination: Ethical approval was received in Bangladesh and Germany. Results will be disseminated through peer-reviewed publications and presentations in Bangladesh and internationally.

Trial Registration: ClinicalTrials.gov ID: NCT02505711. Registered: 22 July 2015.

Strengths and limitations of this study

- The trial includes 96 clusters and used covariate-constrained randomization, thus ensuring good baseline balance.
- We evaluate programme impact on children conceived after the start of the intervention who will have benefitted during their full first 1,000 days of life.
- A wide range of knowledge, behavioural, anthropometric, and biological indicators will be assessed in women and children.
- Rigorous monitoring and surveillance systems provide a platform to examine outputs and outcomes along the causal pathway.
- We measure child growth and blood parameters at baseline in early 2015 and at endline in late 2019, but not continuously over the course of the trial.

For peer review only

Introduction

Child undernutrition is responsible for an estimated 45% of child mortality, about 3.1 million deaths annually (1). Suboptimal nutrition during the first 1,000 days, from conception to two years of age, can have irreversible consequences for child development and long-term health, intelligence, educational attainment and work capacity (1, 2).

To reduce undernutrition sustainably, one must address its complex root causes. The two main direct causes according to the UNICEF and Lancet frameworks are nutrient-deficient diets and frequent gastrointestinal infections (1, 3). An inadequate diet of the mother limits the nutrients the child receives during pregnancy and from breastfeeding, and an inadequate diet of the young child further contributes to undernutrition (1). Nutrient-deficient diets of women and children are often due to insufficient household and maternal knowledge and resources, while unhygienic living conditions favour gastrointestinal infections (1, 3). In addition to the short-term negative influence of infections on appetite and nutrient absorption, frequent subclinical gastrointestinal infections may cause chronic inflammation and damage to the mucosal barrier in the gut and disrupt the intestinal microbiome, leading to "environmental enteric dysfunction", further inhibiting the absorption of nutrients (4).

Due to the many complex causes of undernutrition, programmes that address several underlying factors of child nutrition and health are needed. However, the evidence on "potentially promising and culturally relevant" interventions to diversify diets by "enhancement of agricultural production" has been weak, because most such interventions "have only been implemented at a small scale, and have not been adequately assessed", according to The Lancet's 2008 undernutrition series (5). Evaluations of nutrition-sensitive agricultural interventions, i.e. agricultural interventions focusing not only on yields, but on improving nutrition of vulnerable groups, have been relatively rare, leading to a gap in our understanding of their potential contribution to reducing child undernutrition (6).

For peer review only

Previous evaluations of nutrition-sensitive agriculture programmes found that these can improve household access and consumption of targeted foods and in some cases improve household, maternal, or child dietary diversity, however, the evidence of their impact on child anthropometric outcomes was very weak, according to several reviews in 2012-2013 (6-8). This lack of evidence was largely attributed due to methodological shortcomings of previous studies, such as lack of appropriate control groups and small sample sizes (6-8).

In 2018, Ruel et al. evaluated the recent literature (since 2014) and found several studies which had improved methodologies and examined a wide range of nutrition-sensitive agricultural interventions, including eight homestead food production programmes (9). Ruel et al. report that most studies showed increases in targeted food production, household and child dietary diversity, and in micronutrient intake when measured. Some also showed impacts on anaemia or micronutrient status. However, evidence on stunting was still lacking. Possible reasons cited were short programme durations, poor age targeting (outside the critical 1,000 days), and insufficient statistical power to detect changes in stunting prevalence (9, 10).

With the Food and Agricultural Approaches to Reducing Malnutrition (FAARM) trial, we attempt to address these limitations through a strong cluster-randomised design, sufficient sample size and duration, recruitment strategies to target children in their first 1,000 days, as well as measuring a wide range of indicators along the causal pathway.

Study aims and objectives

The study will evaluate the impact of a Homestead Food Production (HFP) programme implemented by Helen Keller International (HKI) on length-for-age z-score among children aged 0-3 years who were conceived during the intervention period.

For peer review only - http://bmjopen.bmj.com/site/about/guidelines.xhtml

Methods and analysis

Study design

The FAARM trial is a 1:1 cluster-randomised controlled trial with repeated cross-sectional surveys of children born to a cohort of women. To cover the full first 1,000 days of life, we enrolled young, married women into the HFP programme in 2015, with the target group at endline in 2019 being children under 3 years of age conceived by these women during the intervention period.

Study setting

The FAARM trial is conducted in Bangladesh where prevalence of food insecurity and undernutrition is high. In 2014, over half of women consumed inadequately diverse diets, almost a fifth had a BMI below 18.5, and 35% of children under five years were stunted (11). Child marriage and early childbearing are also common in the country: almost half of women had their first pregnancy by age 18 (11). Only 20% of pregnant women receive adequate antenatal care and about 40% of mothers practice exclusive breastfeeding during the first six months (11). Complementary feeding practices for children aged 6-23 months are poor with only 43% receiving an adequately diverse diet (11). In addition, women in Bangladesh and their families struggle with persistent poverty, gender inequality and lack of women's agency, climate change, and political volatility.

Within Bangladesh, the study area was identified through an analysis of population demographics, including proxies for wealth, results from national nutrition surveys, and information about other interventions in the area. Sylhet Division, in north-eastern Bangladesh, has a prevalence of undernutrition above the country average, with 44% of children under five stunted and 40% of pregnant women undernourished in 2014 (11). Sylhet

For peer review only

Division also has the largest wealth disparity and the highest fertility in Bangladesh, and performs worst on a range of gender indicators (12-15).

Sample size

The study sample size was calculated in relation to the primary outcome, length-for-age. A continuous z-score was used, i.e. length in standard deviations below or above the mean for a child of a given age and sex, which can then be translated into the percentage of children "moderately or severely stunted" (below -2 standard deviations). We aimed to detect a minimum difference of 0.20 to 0.25 in mean z-score at a 5% significance level. This effect size has been seen in studies using educational approaches to improve child growth (16).

The sample size for each study arm was first calculated for a simple randomized design and then multiplied by a design factor to account for the clustered design and the greater similarity of children's nutritional status within a cluster. The intra-cluster correlation of length-for-age z-score was estimated to be 0.03, and for a minimum detectable effect size of 0.22 with 90% power, sample size was calculated as 677 children per arm, assuming a cluster size of 15. To account for loss to follow-up, possibly due to late refusal, withdrawal from the study or permanent migration out of the study area, an extra 10% was added. Therefore, we aimed for approximately 745 children per arm, 1490 children in total. Assuming an average of 15 children per cluster, born to 28 women, this meant that nearly 100 clusters needed to be randomized.

Participant recruitment and cluster formation

Once the study site in Baniachong and Nabiganj sub-districts of Habiganj district, Sylhet division, Bangladesh had been selected, we enumerated all households in the area to identify

For peer review only

For peer review only - http://bmjopen.bmj.com/site/about/guidelines.xhtml

women eligible to participate in FAARM. A woman was eligible for inclusion if she reported to be less than 30 years old[1], was married and her husband stayed overnight at the household at least once in the year prior to interview, had access to at least 40 square meters of land (ideally 10 square meters near the house) and was interested to participate in a homestead food production programme (Figure 1).

Once eligible women were identified, clusters (the unit of randomization) were formed with at least 10 and no more than 65 eligible women, based on geographic location of settlements. A minimum distance of 400 meters between settlements was maintained to limit contamination. Figure 2 shows the study site within Bangladesh, and the 96 clusters within 13 unions of Nabiganj and Baniachong sub-districts.

At the start of the baseline survey, trained data collectors approached eligible women for consent who had been assigned to a cluster (Figure 3). Some women were not enrolled into the trial because they had migrated since enumeration, were repeatedly not at home at the time of the survey visits, or refused to participate. During the baseline survey, age was again ascertained using event calendars to get more accurate estimates. In cases where the newly estimated age at baseline was over 30 years, we did not exclude these women from the trial. The youngest biological child of an enrolled woman was assessed at baseline if the child was born after March 1, 2012, three years prior to the first day of the baseline survey training.

During the first year of implementation, we additionally invited women (and their youngest biological children) to enrol who had married into households already participating in the trial

[1] With limited birth records, and official birth records frequently incorrect, many adults cannot accurately report their age.

For peer review only - http://bmjopen.bmj.com/site/about/guidelines.xhtml

(Figure 1). The number of women enrolled in this way was nearly identical in the intervention and control groups.

Cluster randomization

After the baseline survey, 96 clusters were randomly assigned to the intervention or control group (Figure 3). We used covariate-constrained randomization to ensure good balance, i.e. that important baseline characteristics do not differ greatly between arms (17, 18). The randomization was constrained by covariates on the cluster, household, woman, and child level.[2]

The intervention

HKI has been implementing HFP programmes in Bangladesh since 1988, working in partnership with over 70 local non-governmental organizations (NGOs) (19). The objectives of the HFP programmes are to increase household access and consumption of nutrient-dense foods through gardening, poultry rearing and nutrition counselling, and to empower women through increased control of assets and through group leadership and participation. Together these changes are thought to improve the health and nutrition of women and children. Over time, HKI has adapted the programme based on community needs and evolving evidence and expanded to several countries in Africa and Asia (20-22).

Similar to earlier HFP projects, FAARM's HFP programme is implemented jointly with a local partner NGO, the Voluntary Association for Rural Development (VARD). Three full-time technical staff from HKI manage the local project office and provide support and direction to eight full-time project staff, a project coordinator and an accountant from VARD.

[2] Cluster: union and number of women; Household: religion, food consumption score, household food insecurity access score, log of agricultural land size; Women: height, BMI, haemoglobin; Child: age, age-adjusted z-scores of length-for-age and weight-for-length, haemoglobin, diarrhoea prevalence.

For peer review only - http://bmjopen.bmj.com/site/about/guidelines.xhtml
For peer review only

Each project staff member is responsible for a certain geographic region for which they have monthly targets for trainings, individual counselling and asset distribution.

Prior to programme activities, 64 households with existing home gardens were visited in six villages in the project area, interviewed on previous experiences and all food crop species were identified by agricultural researchers. Most gardens had few species and plants and were only active few months per year. Key issues reported were pests, poor soil fertility, excessive shade, and flooding exposure (preventing year-round gardening). Strategies to overcome these were incorporated into the HFP curriculum.

Programme activities begin with organizing project participants into village-level “women farmer groups” of around 16 households each (range from 8 to 26). This is the key intervention unit. Within these groups, members select a lead farmer family, who oversees a village model farm on their property and acts as a multiplier. The lead family receives additional training and is encouraged to implement all recommended improved techniques, produce seeds and seedlings for distribution to other participants, and host trainings and meetings. In larger clusters where one village model farm may provide for several groups, additional women serve as group leaders. At project closeout, at least one motivated participant in each group will be selected as a peer educator to remind neighbours of promoted activities after the intervention has concluded.

FAARM’s HFP intervention includes a series of trainings, usually held at the village model farm. All participants will receive trainings on year-round vegetable gardening, fruit tree production, poultry rearing, and the implications of household production and consumption on health. In the first year, one-day trainings are given on winter and summer crop cultivation, seed production and preservation, and poultry rearing, while half-day refresher trainings are

For peer review only - http://bmjopen.bmj.com/site/about/guidelines.xhtml

given in subsequent years. The trainings emphasize sustainable gardening techniques, including the use of raised beds, live fencing, integrated pest management, composting and soil management, and seed preservation. In the third year of the intervention, once homestead production and consumption are established and there is surplus production, marketing training is given to build linkages with local vendors in order to provide opportunities for income generation for women. In response to issues previously identified in the area, additional trainings are given on sack gardening (during the rainy season when gardens are prone to flooding) and on production and application of urine-enriched biochar fertilizer (to enhance soil fertility), implemented as part of a separate study (23).

Furthermore, project staff conduct courtyard sessions every two months for participants and family members, covering nutrition, health and hygiene topics. An adult learning approach is used, which includes dialogue, story-telling, role play and drawing as well as joint activities such as food preparation. Session content follows the global Essential Nutrition Actions approach, including optimal breastfeeding, infant and young child feeding and nutrition for women (24).

In addition to formal trainings and meetings, project staff visit all participants individually in their households every two months to review nutrition, health and hygiene messages as well as provide technical support for gardening and poultry rearing activities. These interpersonal counselling sessions provide opportunities for participants to bring up issues they are facing that may be difficult to discuss in a group setting and for project staff to tailor messages to the unique situation of the participant and her family.

In the third year of the intervention, an additional food hygiene training is added to reinforce messages on dietary diversity and cover food-related hygiene topics more intensively, in

For peer review only - http://bmjopen.bmj.com/site/about/guidelines.xhtml

particular hand-washing, clean utensils, food storage, and re-heating (see Supplementary File 1). This intervention is delivered by eight female staff and consists of eight structured events, four group events and four household visits.

FAARM's HFP intervention also transfers assets. Seeds are distributed to all project households twice a year to coincide with the main planting seasons and include nutrient-rich, seasonally and locally appropriate vegetables such as red amaranth, water spinach, tomato, pumpkin, string bean and cabbage. Similarly, tree saplings, chosen for nutrient-rich fruits such as papaya, lemon and jujube, were transferred to households with greater land availability. Participants also receive poultry assets, primarily a reimbursement for the majority of the cost of a constructed, improved poultry shed, as well as starter feed and watering stations.

A gender-equitable approach is integrated into the HFP structure with the programme aiming to increase women's status in the household through asset transfer and control of productive resources as well as the skill transfer associated with HFP trainings. Improving women's status and agency is thought to further support programme activities and improve child nutrition (25).

Programme theory

A conceptual framework (Figure 4) was constructed to illustrate the HFP programme's theory of change. Training on gardening and poultry rearing can increase household access to nutrient-dense foods, counselling on health, hygiene, and child care topics may lead to improved practices, and the marketing of surplus products can increase household income. Throughout the programme, the focus on women's participation and leadership may increase women's empowerment by increasing their decision-making role and social support. When

For peer review only

these outcomes are achieved, we expect to see improvements in dietary intake and reduced infections, leading to reduced maternal and child undernutrition and better child development.

Control arm

In both intervention and control settlements, there are certain benefits delivered alongside the data collection activities. These include free pregnancy tests, birth counselling to establish a birth plan, and a visit within three days of birth where staff weigh and measure the child and provide breastfeeding counselling to the mother.

Monitoring plan

FAARM's monitoring plan consists of three major elements: intervention activity records, output monitoring of intervention households, and a multi-part surveillance system that includes both intervention and control households (Figure 3). Indicators measured through these systems are outlined in a detailed project monitoring plan based on identified project impact pathways. All systems gather data using ODK Collect software (www.ona.io) on android mobile devices.

Intervention activity records

Project monitoring forms were designed to track programme contact points, including asset distribution, attendance of each participant and her family members at courtyard sessions and trainings, and tracking when and what topics were included in counselling sessions. In addition, supervisors document their monitoring of project staff activities.

Output monitoring

Project staff conduct monitoring interviews of FAARM intervention participants on a rolling basis, with each respondent visited once per year (Table 1). In these interviews, outputs and

For peer review only

outcomes, such as garden diversity and yields, poultry production, and knowledge gained from training modules are assessed in the intervention arm (Figure 4).

Surveillance

FAARM's surveillance system in intervention and control households contains three components explained in detail below: 1) routine assessment, 2) food consumption assessment, and 3) birth registration (Figure 3). It serves to examine indicators on the two main pathways, diet and infection, through which the intervention could impact child undernutrition, as well as assessing maternal undernutrition and intrauterine growth retardation (Figure 4). All surveillance activities are conducted by locally-recruited, female data collectors. Extensive training with field practice is provided before any data collector begins work. Before each two-month data collection period, a 1-2-day refresher training is held to review and troubleshoot as well as train on any questionnaire aspects that have been added or changed. Collected data are downloaded and reviewed regularly for real-time monitoring and analysis. The surveillance system is summarized in Table 1, while information on indicators collected is available in Table S1.

A routine assessment round is completed every two months. Women report their last menstrual periods using provided calendars to mark the day. Pregnancies are identified with the help of pregnancy tests, which are provided for free to women for voluntary self-administration. Pregnant women are asked about antenatal care, and their weight and mid-upper arm circumference are measured at every visit, while non-pregnant women are weighed once per year. Dietary diversity information is recorded from all pregnant women each round and from non-pregnant women every six months using 21 food groups (26). The Edinburgh Perinatal/Postnatal Depression Scale is administered during pregnancy, shortly after delivery, and after the child has attained six months of age (27, 28). For all children who are below three years of age (at the first day of each two-month round), we ask mothers about recent

For peer review only

diarrhoea and acute respiratory infection episodes. Mothers of children 0 to 18 months are asked all questions from the standard WHO Infant and Young Child Feeding survey module each round, while children above 18 months are asked these questions every six months (29). Starting in the second year of the trial, the six WHO motor milestones are measured on all children as appropriate for their age (30). Starting in the third year of the trial, all children have their weight recorded around 18 months of age. Every four months, women are asked about the different crop species grown in their gardens in the previous season (cool-dry, hot-dry, rainy) to assess diversity of production. Annually, questions are asked about dietary habits during Ramadan. Periodically, additional questions are added, e.g. about poultry production, garden characteristics, household hygiene, and food security.

Detailed quantitative food consumption information is recorded through dietary recall on a randomly selected subset of nine households per cluster, i.e. a total of 864 households. To achieve even distribution over seasons and arms, the 96 clusters were divided into two phases of 48 clusters, with 24 intervention and 24 control clusters in each phase. Data are collected every two months, i.e. six times per year, alternating between phases, with each cluster thus being visited three times per year to interview three households each time. Total food consumed by the household and how this was distributed among all household members is reported by the primary food preparer. A separate module about snacks and food intake between meals is asked to all available household members separately. In a third of the sample each round (one of the three households per cluster), diets are recorded on two non-consecutive days, less than ten days apart, in order to enable estimation of usual intake and the proportion of the population at risk of inadequate intake. The methods used in data collection are derived from those designed by International Food Policy Research Institute

For peer review only

(IFPRI) for the Bangladesh Integrated Household Survey [18]. This procedure is repeated annually in the same households in the same season.

Birth registration activities are initiated on all identified pregnancies in the third trimester. Pregnant mothers are provided with free phone cards and encouraged to notify the team when they have delivered. An automated calling list is used by birth registration team members to give reminder calls to the pregnant women before their estimated date of birth. As soon as notified, ideally within 72 hours of birth, a trained female data collector visits the family, measures the newborn's weight, length, and head circumference, interviews the mother on the delivery, and provides initial lactation counselling. Birth registration data collectors are trained on standard anthropometric measurement techniques for newborns and calibrate measurement equipment on each day of use.

Evaluation plan

Trial outcomes and measurements

Primary outcome

The primary outcome measure is length-for-age z-score in children under three years of age at endline, using the 2006 WHO child growth standards (31).

Secondary outcomes

Secondary outcome measures include other measures of child growth, namely weight-for-length and head circumference-for-age z-scores in children 0-3 years; and length-for-age z-score in children 6-30 months, as well as growth outcomes at birth (length-for-gestational-age, weight-for-gestational-age, and head circumference-for-gestational-age). For women's

For peer review only

439 nutritional status, indicators are BMI in non-pregnant women, and mid-upper arm
440 circumference and gestational weight gain in pregnant women. Micronutrient status is
441 measured through anaemia (haemoglobin), vitamin A deficiency (retinol-binding protein),
442 iron deficiency (serum ferritin, soluble transferrin receptor), and zinc deficiency (serum zinc)
443 in women and children, adjusting for inflammation (serum C-reactive protein, serum α-1-acid
444 glycoprotein) when recommended (Table S2 & Supplementary File 2).

445 Dietary diversity is measured according to published recommendations, using 10 food groups
446 for women and 7 food groups for children (26, 29). In addition, dietary adequacy is assessed
447 for a subsample of women and children using the mean probability of adequate intake for 11
448 micronutrients (thiamine, riboflavin, niacin, vitamin B6, folate, vitamin B12, vitamin C,
449 vitamin A, calcium, iron, zinc). Indicators for infections in children comprise 7-day period
450 and 2-day point prevalences of diarrhoea and of acute respiratory infection. Early child
451 development is assessed using WHO motor milestones (30, 32).

452 Food hygiene indicators include a hygiene behaviour score measured through structured
453 observation in mothers with children 6-18 months of age, faecal contamination (total
454 coliforms and *E. coli*) of complementary food samples, and enteropathy biomarkers (faecal
455 myeloperoxidase, faecal α1-antitrypsin, faecal neopterin, serum C-reactive protein, serum α-
456 1-acid glycoprotein). This is conducted through the "Food Hygiene to reduce Environmental
457 Enteric Dysfunction" study nested into the FAARM trial (see Supplementary File 1).

458 *Additional outcomes*

459 The FAARM trial provides an ideal platform on which to explore other research topics, such
460 as linkages between food production and diets, women's agency and mental health, child
461 language development, dietary influences on arsenic methylation capacity, mycotoxin
462 exposure, (epi-)genetic factors influencing innate immunity and micronutrient metabolism, as
463 well as diversity and composition of the mother's and the child's gut microbiome through

For peer review only

464 which the above effects may be modified. Several of these additional research components
465 will be explored during the course of the trial, contingent on time and funding.
466
467 All measurements and samples are taken in accordance with current standards and
468 methodology, building on questionnaire tools from HKI and James P Grant School of Public
469 Health at BRAC University, including from the Food Security and Nutritional Surveillance
470 Project in Bangladesh (11). HKI employs experienced field research assistants who receive
471 specific training and supervision on anthropometric measurement and interview techniques.
472 All blood sampling is undertaken by clinically trained staff, familiar with the appropriate
473 methods of extraction, storage and processing.
474
475 *Data collection*
476 Most trial outcomes are measured during the endline survey in late 2019 (Figure 3). A week-
477 long training is held for all data collectors, with separate trainings conducted for questionnaire
478 interviewers, anthropometry and blood sampling staff, following standard measurement
479 procedures (33-35). HKI staff conduct trainings for interviewers, covering survey question
480 content, question-asking techniques, and cultural sensitivity, and for the anthropometry team,
481 standardization exercises with local volunteers. Staff from icddr,b are responsible for training
482 and supervision of blood and stool sampling and processing. Supervisors and trainers conduct
483 quality control in the field and refresher trainings are given when needed. Data are collected
484 via tablets and uploaded after each interview or at the end of the day. Datasets are examined
485 for quality and adjustments to the questionnaires or refresher trainings with the data collectors
486 are done as needed.
487
488 ***Data management***

For peer review only - http://bmjopen.bmj.com/site/about/guidelines.xhtml

Questionnaires have been programmed to prevent common data collection errors. Data are downloaded and compiled on a regular basis, and data quality checks are conducted. Data concerns are immediately communicated to the data collectors or research team for resolution. All quantitative and qualitative data are stored on password-protected servers and computers. Only deidentified data are shared with collaborators for analysis.

Statistical analysis plan

The main analysis will be to compare the mean length-for-age z-score in children, aged 0-35 months at the start of the endline survey, between intervention and control arms. This will be done using mixed effects linear regression, i.e. modelling between-cluster variation as a random effect in a full probability model. This method will be statistically more efficient than analysing results at the cluster level and also allows for analysis of individual-level covariates (36). In order to reduce between-cluster variation and thus increase power and precision of the study, we will adjust for baseline values of the main outcomes of interest (e.g. mean length-for-age at cluster level) as well as potentially other relevant predictors. Secondary and other outcomes that are normally distributed will be analysed in the same way. Logarithmic transformation will be used for continuous variables with a skewed distribution (e.g. agricultural yields). Binary outcomes (e.g. health service use) will be analysed with mixed effects logistic regression models. Missing data on outcome measurements, including loss to follow-up, will be compared between intervention and control arms with respect to baseline covariates to assess the potential for selection bias.

Patients and public involvement

Participants were not involved in the study design or in the setting of research objectives or outcomes. Intervention implementation is adapted to participant concerns and constraints. Key stakeholders will be involved in dissemination of research findings.

For peer review only

Ethics and dissemination

Ethical approvals

The full FAARM trial protocol was positively reviewed by the University of Heidelberg in Germany (Reference: S-121/2014). The trial protocol without biological sample collection was approved by the James P. Grant School of Public Health, BRAC University (IRB Registration: IRB00003441) and the blood sampling by the Bangladesh Medical Research Council (Reference: BMRC/NREC/2013-2016/844). The food hygiene intervention and evaluation protocol including stool sampling and blood sampling at endline was approved by the University of Heidelberg (Reference: S-606/2017) and the International Centre for Diarrhoeal Disease Research, Bangladesh (Reference: PR-17126). Any protocol changes are reported to the respective ethical boards for approval. The FAARM trial is registered at Clinicaltrials.gov (ID: NCT02505711).

Consent

All enrolled FAARM participants provide informed, written consent by signature, or thumbprint if illiterate, for themselves and for their children, if applicable. All participants receive an information sheet with contact information of the local FAARM manager for any questions or concerns.

Risks and benefits

The risks from participation (time expenditure over four years, anthropometric measurement and biological sampling) are minimal when compared to the potential knowledge gained from this study. There are some benefits for all participants, including referral for serious conditions when detected during surveys, early pregnancy identification (through distribution

For peer review only

of pregnancy tests), and initial breastfeeding counselling. Participants in the intervention arm additionally benefit from intervention activities as they receive assets, training and support to adopt promoted behaviours.

All participants are informed about their anthropometric measurements in cases of low BMI or child growth, and haemoglobin values when anemic. The results of the trial will be disseminated to all research and implementation partners, published in peer-reviewed journals, and presented at national and international conferences and events. Programme- and policy-related findings will be distributed through the HKI network and other agricultural and nutrition fora.

Project status

As of April 2019, participant enrolment, baseline survey and intervention activities have been completed. The monitoring, and surveillance activities are ongoing and the endline survey is planned for late 2019.

Discussion

To our knowledge, this is the first randomized controlled trial to assess the impact of a complex, nutrition-sensitive agricultural programme on children's nutritional status that enrols women prior to pregnancy, thus capturing the full first 1,000 days of child development. It is particularly relevant and well-placed in rural Bangladesh where stunting and micronutrient deficiencies are common. We will be able to assess not only *whether* the intervention has impacted each outcome but also *how* this occurs, due to a comprehensive monitoring and surveillance system through which we track programme fidelity and intensity, participation, and outcomes along impact pathways. By assessing participant knowledge and behaviours as well as anthropometric and biological measures, we will be able to examine

For peer review only

changes on a wide range of indicators. In addition, the rotating data collection pattern of the surveillance system will allow the project to map out changes in diet over seasons and between intervention and control households over time. Data on the sources of foods consumed by households will enable tracking of changes in food consumption habits, comparing garden production to market purchases. A limitation of our study is the lack of continuous child growth monitoring and blood sampling during surveillance, due to financial and logistic constraints. Data collection of children will also end at three years of age, which may miss growth or child development impacts which appear later.

We expect the findings from this trial will contribute to national policy discussions regarding the contribution of complex nutrition-sensitive agricultural interventions to improving nutritional status and other outcomes. Furthermore, trial results may be generalizable to other settings where poverty, food insecurity and climate change affect nutrition and health.

Acknowledgements

The authors would like to thank the generous funders that make this research possible. We also thank all researchers who contributed their time and knowledge to this study, in particular Tarique Huda, Shafinaz Sobhan, Philipp Zanger, Nicholas Kyei, Andreas Deckert, Andrea Seiermann, Simone Jacobs, Malabika Sarker, Irmgard Jordan, Katja Kehlenbeck, Sebastian Kern, Hans-Peter Schmidt, Lisa Hefele, Wolf-Peter Schmidt, Jody Harris, Om Prasad Gautam, and Sheela Sinharoy as well as our administrator Elke Braun-van der Hoeven, and Gabriele Stieglbauer for assistance with data management. We furthermore thank HKI for their support in establishing and conducting the FAARM trial, in particular the leadership provided by Zaman Talukder, Meredith Jackson-de Graffenried, Sheela Sinharoy, Amin Uddin and Shirin Afroz at the HKI country office, Nancy Haselow and Ame Stormer at the HKI regional office, and Rolf Klemm and Jennifer Nielsen at HKI headquarters. Most

For peer review only

importantly, the trial would not be possible without the dedicated research and implementation staff working on the ground to carry the project to fruition. We thank the HKI research team led by Abdul Kader, with field leadership from Shobha Tripura and Sailen Akhter and analysis support from Chowdhury Asif, Md. Arafat Rahman, and Masum Ali. We also warmly thank our implementation team from HKI – Zillur Rahman, Basana Marma and Md. Wali-Ul Islam – and the VARD team carrying out the daily work in the villages. Lastly, we want to thank all the women, children and families in Sylhet who generously give their time and their effort to participate in FAARM.

Author contributions

SG conceptualized the study initially, then jointly with JLW. SG, JLW and TMS selected the study site. SG, JLW, ASW, TMS and AAM developed and oversaw surveys, monitoring and surveillance systems. All authors contributed to the development, review, and finalization of this study protocol manuscript.

Funding statement

The FAARM trial is primarily supported by the German Ministry for Education and Research (BMBF) (Award number: 01ER1201). Additional funding for the surveillance programme and other project components comes from the Nutrition Embedding Evaluation Programme (NEEP), funded by the UK Department for International Development (DFID), the Food & Business Applied Research Fund (ARF) of the Dutch Ministry of Foreign Affairs, the Thrasher Research Fund, and the Alexander von Humboldt Foundation. Further support through charitable donations to HKI for the implementation of the HFP programme is provided by the Carrefour social responsibility programme and the Best in Brands social marketing programme. Funding organizations did not have a role in the study design, hiring or training of staff, intervention delivery, data collection, analysis, or interpretation of data.

For peer review only

616

617 **Declaration of interests**

618 The authors declare no conflict of interest. The content of this publication is solely the

619 responsibility of the authors.

For peer review only

References

1. Black RE, Victora CG, Walker SP, Bhutta ZA, Christian P, de Onis M, et al. Maternal and child undernutrition and overweight in low-income and middle-income countries. Lancet. 2013;382(9890):427-51.

2. Ruel M, Hoddinott J. Investing in early childhood nutrition. IFPRI Policy Briefs. Washington DC: International Food Policy Research Institute; 2008.

3. UNICEF. Strategy for improved nutrition of children and women in development countries. New York, USA: UNICEF; 1990.

4. Harper KM, Mutasa M, Prendergast AJ, Humphrey J, Manges AR. Environmental enteric dysfunction pathways and child stunting: A systematic review. PLoS Negl Trop Dis. 2018;12(1):e0006205.

5. Bhutta ZA, Ahmed T, Black RE, Cousens S, Dewey K, Giugliani E, et al. What works? Interventions for maternal and child undernutrition and survival. Lancet. 2008;371(9610):417-40.

6. Masset E, Haddad L, Cornelius A, Isaza-Castro J. Effectiveness of agricultural interventions that aim to improve nutritional status of children: systematic review. BMJ. 2012;344:d8222.

7. Ruel MT, Alderman H. Nutrition-sensitive interventions and programmes: how can they help to accelerate progress in improving maternal and child nutrition? Lancet. 2013;382(9891):536-51.

8. Girard AW, Self JL, McAuliffe C, Olude O. The effects of household food production strategies on the health and nutrition outcomes of women and young children: a systematic review. Paediatr Perinat Epidemiol. 2012;26 Suppl 1:205-22.

9. Ruel MT, Quisumbing AR, Balagamwala M. Nutrition-sensitive agriculture: What have we learned so far? Global Food Security. 2018;17:128-53.

10. Harris J, Thompson S, Sparling T. Leveraging Development Programs: Homestead Food Production. In: Ferranti P, Berry EM, Anderson JR, editors. Encyclopedia of Food Security and Sustainability. Oxford: Elsevier; 2019. p. 396-400.

11. Helen Keller International (HKI), James P. Grant School of Public Health (JPGSPH). State of Food Security and Nutrition in Bangladesh: 2014. Dhaka, Bangladesh: HKI and JPGSPH; 2016.

12. Gottlieb CA, Maenner MJ, Cappa C, Durkin MS. Child disability screening, nutrition, and early learning in 18 countries with low and middle incomes: data from the third round of UNICEF's Multiple Indicator Cluster Survey (2005-06). Lancet. 2009;374(9704):1831-9.

13. Monitoring the Situation of Children and Women, Bangladesh: Multiple Indicator Cluster Survey 2009. Bangladesh Bureau of Statistics, Ministry of Planning and United Nations Children's Fund (UNICEF); 2010.

14. Suan Pheng Kam MLB, Tahmina Latif, M A H Chowdhury, S Ghulam Hussain, Mahbub Ahmed, Anwar Iqbal, L Villano, Mahabub Hossain. Geographical Concentration of Rural Poverty in Bangladesh. Dhaka, Bangladesh: Center for Policy Dialogue; 2004.

15. National Institute of Population Research Training - NIPORT/Bangladesh, Mitra Associates, ICF International. Bangladesh Demographic and Health Survey 2014. Dhaka, Bangladesh: NIPORT, Mitra and Associates, and ICF International; 2016.

16. Dewey KG, Adu-Afarwuah S. Systematic review of the efficacy and effectiveness of complementary feeding interventions in developing countries. Matern Child Nutr. 2008;4 Suppl 1:24-85.

For peer review only

17. Ivers NM, Halperin IJ, Barnsley J, Grimshaw JM, Shah BR, Tu K, et al. Allocation techniques for balance at baseline in cluster randomized trials: a methodological review. Trials. 2012;13:120.
18. Lorenz E, Gabrysch S. Covariate-constrained randomization routine for achieving baseline balance in cluster-randomized trials. Stata Journal. 2017;17(2):503-10.
19. Helen Keller International. Homestead Food Production [Available from: http://www.hki.org/reducing-malnutrition/homestead-food-production/.
20. Olney DK, Vicheka S, Kro M, Chakriya C, Kroeun H, Hoing LS, et al. Using program impact pathways to understand and improve program delivery, utilization, and potential for impact of Helen Keller International's homestead food production program in Cambodia. Food Nutr Bull. 2013;34(2):169-84.
21. Nielsen JN, Olney DK, Ouedraogo M, Pedehombga A, Rouamba H, Yago-Wienne F. Process evaluation improves delivery of a nutrition-sensitive agriculture programme in Burkina Faso. Matern Child Nutr. 2018;14(3):e12573.
22. Improving diets and nutrition: food-based approaches. Thompson B, Amoroso L, editors. Rome, Italy: CABI International and FAO; 2014.
23. Scaling-up “Biochar-urine nutrient cycling for health” in Bangladesh (BUNCH2Scale) 2017 [cited 2019 January 9]. Available from: http://knowledge4food.net/research-project/bangladesh-bunch2scale/.
24. World Health Organization (WHO). Essential Nutrition Actions: Improving maternal, newborn, infant and young child health and nutrition. Geneva, Switzerland: WHO; 2013.
25. Talukder A, Haselow NJ, Osei AK, Villate E, Reario D, Kroeun H, et al. Homestead food production model contributes to improved household food security and nutrition status of young children and women in poor populations. Field Actions Science Reports. 2000(Special Issue 1).
26. FAO, FHI 360. Minimum Dietary Diversity for Women: A Guide to Measurement. Rome, Italy: FAO; 2016.
27. Cox JL, Holden JM, Sagovsky R. Detection of postnatal depression. Development of the 10-item Edinburgh Postnatal Depression Scale. Br J Psychiatry. 1987;150:782-6.
28. Gausia K, Hamadani JD, Islam MM, Ali M, Algin S, Yunus M, et al. Bangla translation, adaptation and piloting of Edinburgh Postnatal Depression Scale. Bangladesh Med Res Counc Bull. 2007;33(3):81-7.
29. World Health Organization (WHO). Indicators for assessing infant and young child feeding practices: Part 2 Measurement. Geneva, Switzerland: WHO; 2010.
30. WHO Motor Development Study: windows of achievement for six gross motor development milestones. Acta Paediatr Suppl. 2006;450:86-95.
31. World Health Organization (WHO). WHO child growth standards : length/height-for-age, weight-for-age, weight-for-length, weight-for-height and body mass index-for-age : methods and development. Geneva, Switzerland: WHO; 2006.
32. WHO Anthro for personal computers: Software for assessing growth and development of the world’s children. 3.2.2 ed. Geneva: WHO; 2010.
33. World Health Organization (WHO). WHO guidelines on drawing blood: best practices in phlebotomy. Geneva, Switzerland: WHO; 2010.
34. Cogill B. Anthropometric Indicators Measurement Guide. Washington, D.C.: Food and Nutrition Technical Assistance Project, Academy for Educational Development; 2003.

For peer review only

35. Cashin K, Oot L. Guide to anthropometry: a practical tool for program planners, managers, and implementers. Washington, D.C.: Food and Nutrition Technical Assistance III Project (FANTA) / FHI 360; 2018.

36. Hayes RJ, Moulton LH. Cluster Randomised Trials: Chapman & Hall/CRC; 2009.

For peer review only

726

Table 1: FAARM data sources and data collection methods

	Data source	Data collection device	Responsible team	Population	Frequency
Survey	Baseline	Tablet	Survey team	Intervention and control group	Once in 2015
Programme monitoring	Intervention activity records	Paper, then Smartphone	Implementation team	Intervention group	Rolling: During or after programme activities
Programme monitoring	Output monitoring	Smartphone	Implementation team	Intervention group	Annually on a rolling basis
Surveillance	Routine assessment	Tablet	Surveillance team	Intervention and control group	Every 2 months
Surveillance	Food consumption	Tablet	Surveillance team	Sub-sample: Intervention and control group	Annually on a rolling basis
Surveillance	Birth registry	Tablet	Surveillance team	At birth: Intervention and control group	Within 72 hours of childbirth
Survey	Endline	Tablet	Survey team	Intervention and control group	Once in 2019

727

728

For peer review only

Figure titles and legends

Figure 1: Flowchart of FAARM participant selection and randomization
FAARM: Food and Agricultural Approaches to Reducing Malnutrition

Figure 2: FAARM study site

The large map shows the FAARM study site with 96 clusters in 13 unions of 2 sub-districts (Nabiganj and Baniachong) and the small map on the top shows its location within Habiganj District and Sylhet Division in Bangladesh.
FAARM: Food and Agricultural Approaches to Reducing Malnutrition

Figure 3: Activity timeline for FAARM trial in Sylhet, Bangladesh
FAARM: Food and Agricultural Approaches to Reducing Malnutrition

Figure 4: FAARM conceptual framework
The blue boxes represent components of Homestead Food Production intervention trainings and counselling sessions. These will influence the outcomes listed (the green boxes) with women's empowerment interacting with all aspects. Better outcomes then lead to improved impacts including child development and growth.
FAARM: Food and Agricultural Approaches to Reducing Malnutrition

For peer review only

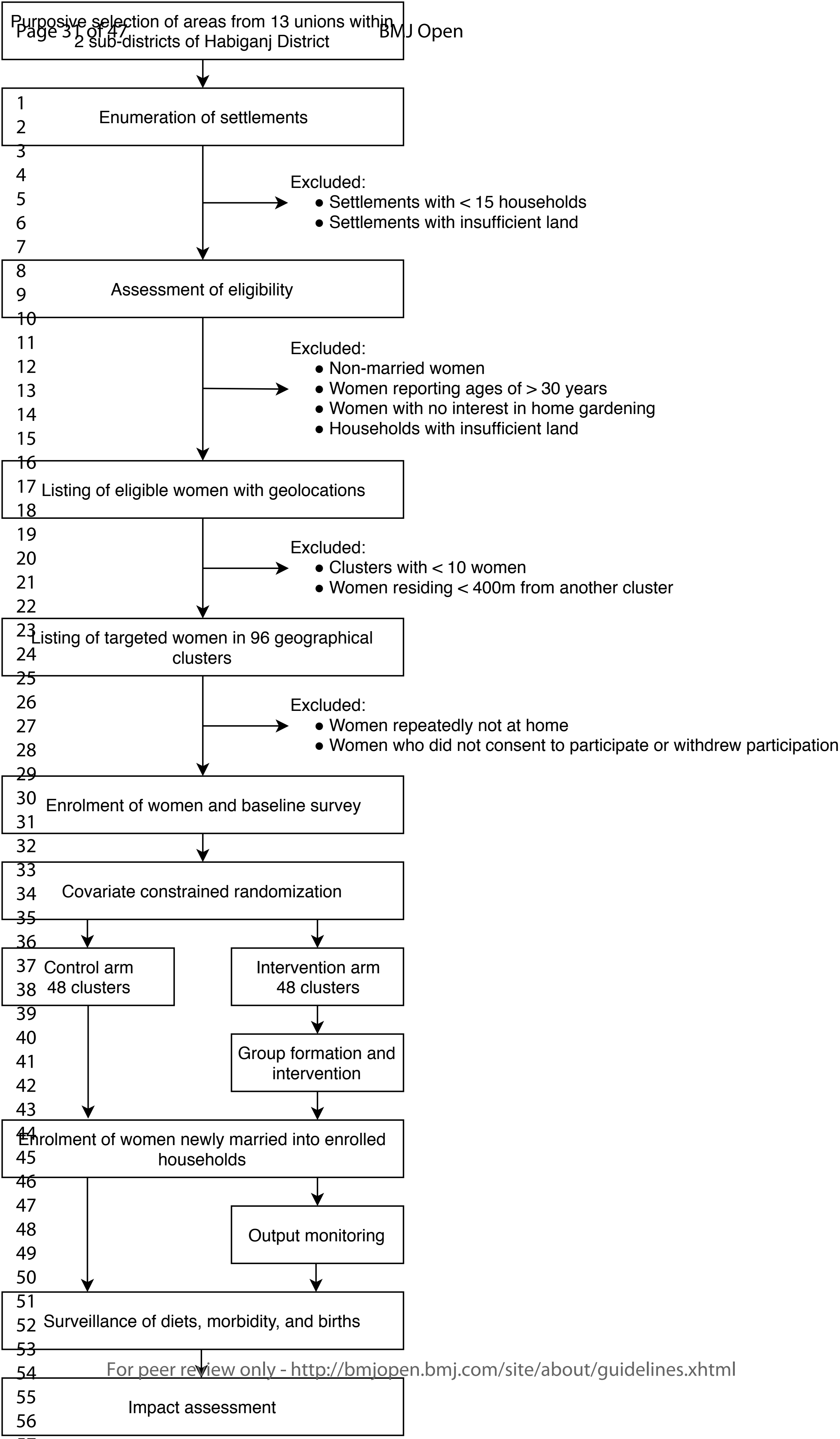
Purposive selection of areas from 13 unions within 2 sub-districts of Habiganj District
Enumeration of settlements
Excluded:
● Settlements with < 15 households
● Settlements with insufficient land
Assessment of eligibility
Excluded:
● Non-married women
● Women reporting ages of > 30 years
● Women with no interest in home gardening
● Households with insufficient land
Listing of eligible women with geolocations
Excluded:
● Clusters with < 10 women
● Women residing < 400m from another cluster
Listing of targeted women in 96 geographical clusters
Excluded:
● Women repeatedly not at home
● Women who did not consent to participate or withdrew participation
Enrolment of women and baseline survey
Covariate constrained randomization
Control arm 48 clusters
Intervention arm 48 clusters
Group formation and intervention
Enrolment of women newly married into enrolled households
Output monitoring
Surveillance of diets, morbidity, and births
Impact assessment

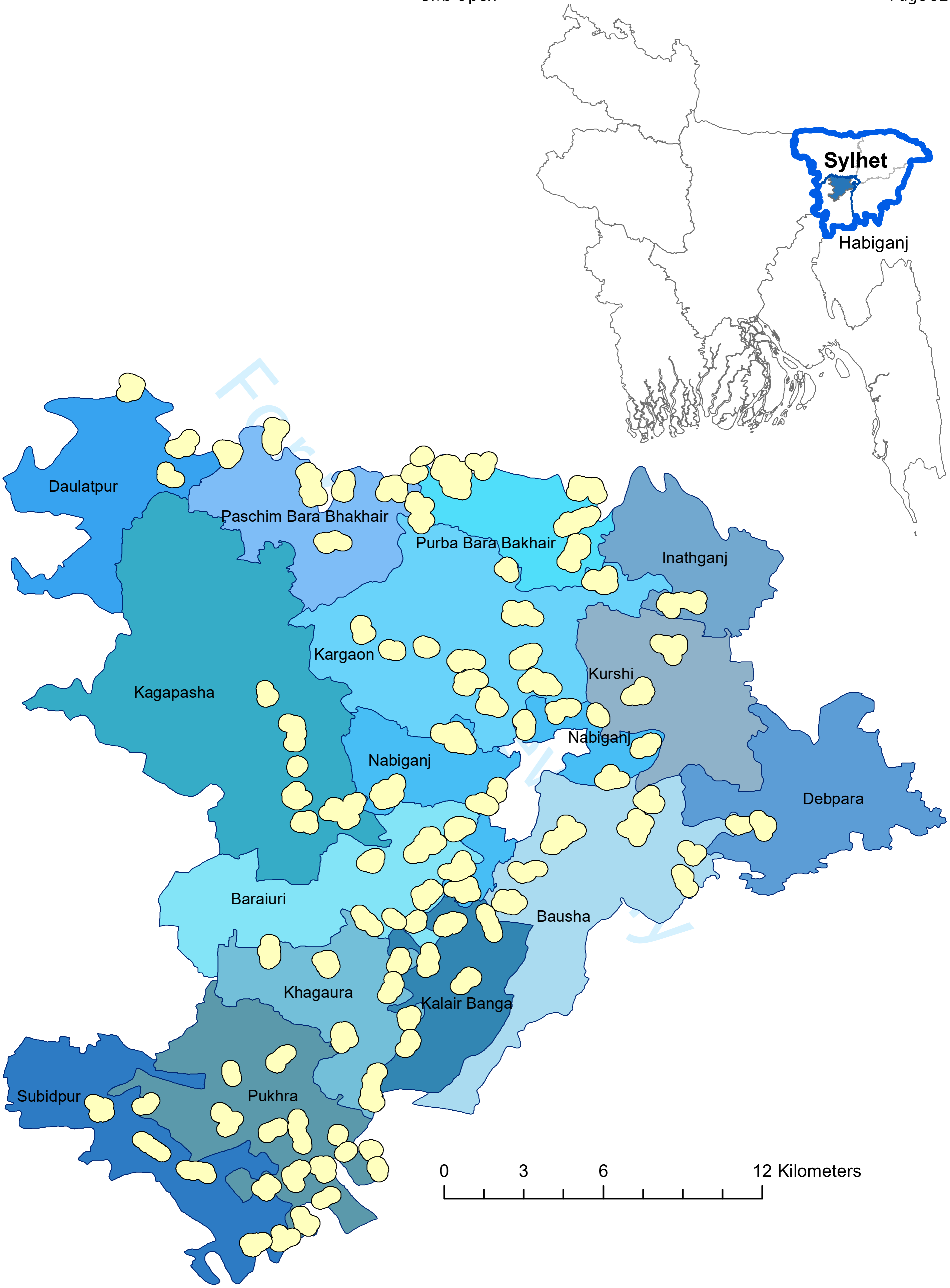
Sylhet
Habiganj
Daulatpur
Paschim Bara Bhakhair
Purba Bara Bakhair
Inathganj
Kargaon
Kurshi
Kagapasha
Nabiganj
Nabiganj
Debpara
Baraiuri
Bausha
Khagaura
Kalair Banga
Subidpur
Pukhra
0
3
6
12 Kilometers

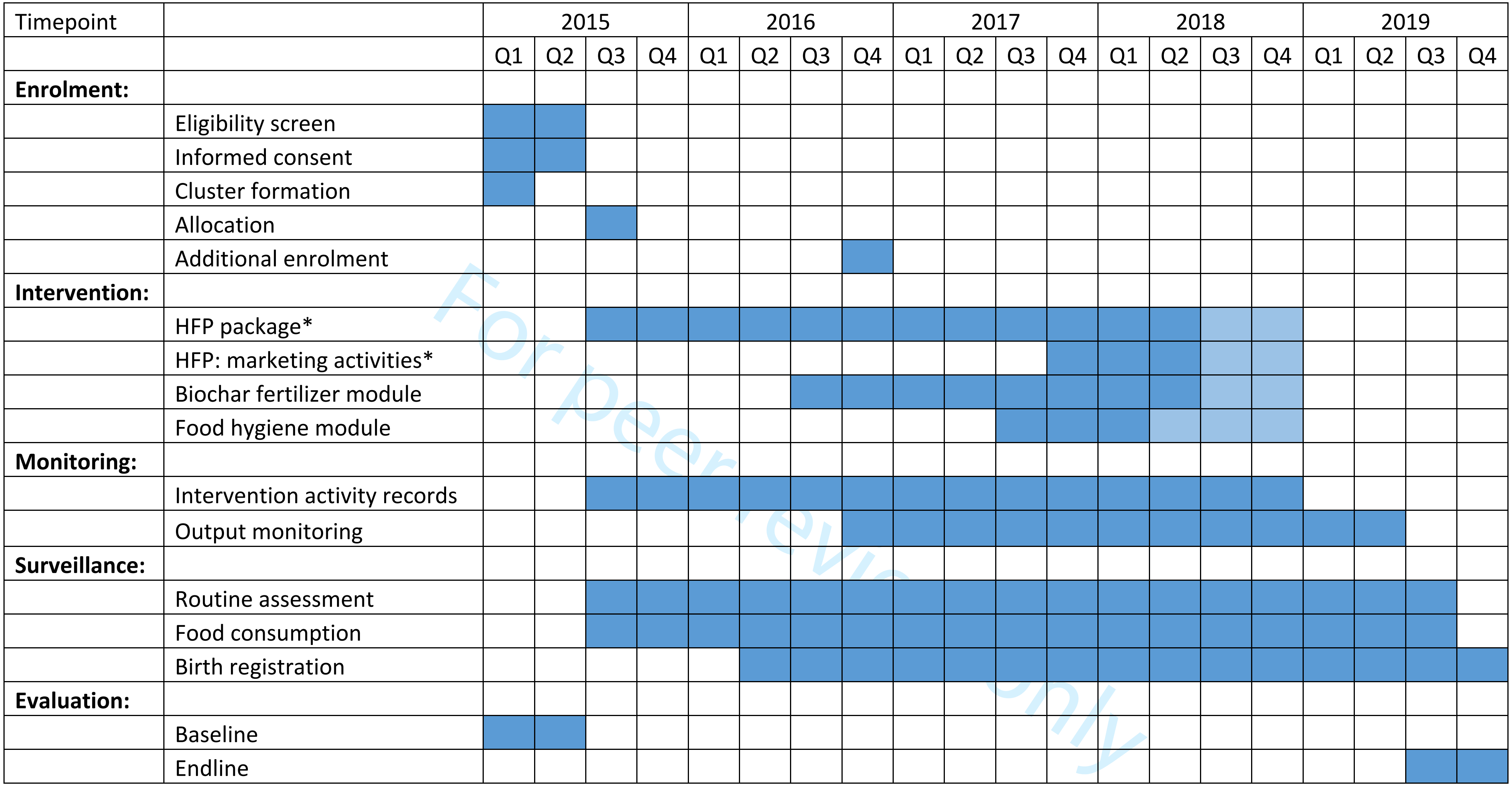

* Lighter blue denotes a gradual lowering of the intervention intensity, with only household counselling sessions.

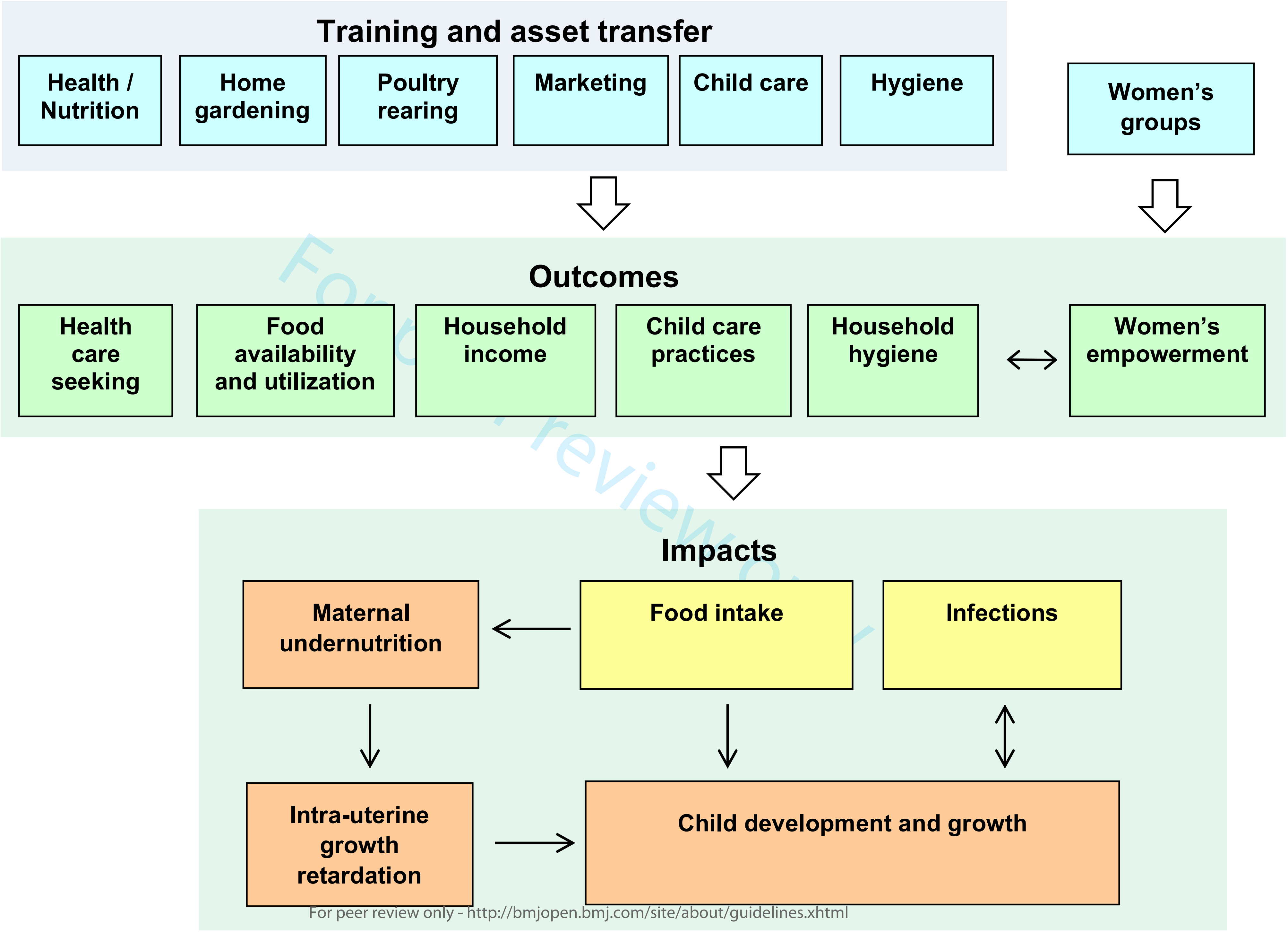

For peer review only - http://bmjopen.bmj.com/site/about/guidelines.xhtml

Supplementary File 1: Food Hygiene to reduce Environmental Enteric Dysfunction (FHEED)

In addition to promoting homestead gardens and nutrition education, a special behavior change intervention has been designed to strengthen the food hygiene component in FAARM. This food hygiene intervention uses a behavior-centered design by employing emotional drivers (such as pride, disgust and nurture) as well as engaging group activities (including games and competitions) and repeated individual household visits to improve common food hygiene practices among study participants.

The intervention focuses on the improvement of six identified key optimal feeding and food hygiene behaviors: exclusive breastfeeding up to 6 months, improved diversity and nutritional value of food (to achieve integration with the Homestead Food Production messages), cleanliness of serving utensils, proper hand-washing before feeding/eating, adequate food storage, and thorough reheating of stored food.

The food hygiene intervention is delivered to groups of 5 to 25 women by eight food hygiene promoters through two core components: group events, which facilitate exchange and interactive discussions, and individual household visits, which provide the opportunity of one-on-one counselling on key behaviors and individual questions. In total, the food hygiene promoters conducted four group events per women group and four household visits per woman over an eight-month period (July 2017 to February 2018).

The FHEED study was added as sub-study to the FAARM trial to evaluate the impact of food hygiene on the development of intestinal infections and Environmental Enteric Dysfunction (EED), as a second key pathway to child growth alongside nutrition. It specifically studies factors on the pathway from food hygiene to EED, including women´s food hygiene behaviour (measured through structured observation), complementary food contamination (log-transformed counts of total coliforms and *E.coli* in food samples), intestinal pathogen load (pathogen-specific qRT-PCR), diarrheal episodes and EED biomarkers (fecal myeloperoxidase, fecal α1-antitrypsin fecal neopterin, serum C-reactive protein, serum α1-acid glycoprotein). The FHEED study will assess these parameters in children aged 0-18 months in two cross-sectional surveys (in 2018 and 2019). In addition, children born in 2018 after the end of the food hygiene intervention will be enrolled in a cohort and sampled every two months over a 12-month period.

For peer review only

Supplementary File 2:

Blood samples:

During the baseline survey in 2015, 300 µL of whole capillary blood was collected (in Microvette™ tubes) from enrolled women and children. Blood samples were then transported to the field laboratory maintaining a cold chain (+4°C to +8°C at all times) and stored in a refrigerator at 4°C over night. The following morning, samples were centrifuged in an Eppendorf Mini-Spin centrifuge for 10 minutes at 13,400 rounds per minute. The supernatant was pipetted into 50 µL aliquots (approximately 2-3 per sample). Serum and red blood cells were then stored in a -20°C freezer on site. Samples that were transported to Germany on dry ice were again stored in a -20°C freezer before analysis.

At the endline survey in 2019, we will collect venous blood from enrolled woman (in total 8 mL) and children (in total 5 mL) into the Vacutainer™ collection system EDTA and serum tubes, through puncture of a cubital vein. Blood samples will be transported to the field laboratory maintaining a cold chain (+4°C to +8°C at all times) and stored in a refrigerator at 4°C over night. The following morning, EDTA samples will be used for complete blood count analysis. Serum and red blood cells will be stored in a -20°C freezer on site. Samples will then be transported to Germany on dry ice and again stored in a -20°C freezer before analysis.

Faecal samples:

Stool samples are collected during a cross-sectional survey in all children aged 0-18 months in 2018 and at study endline in 2019. In a subset (150 households), we additionally collect maternal stool samples. Stool collection sets (stool collection tubes, a sterile stick for stool collection and gloves) are handed to the mothers with instructions one day prior to collection. We collect one native stool sample and one stool sample preserved in 7 mL RNA-later for nucleic acid extraction. Stool samples are aliquoted and stored in a -20°C freezer on site until shipment to Germany on dry ice for further analysis and long-term storage at -80°C.

For peer review only

Table S1: Surveillance indicators collected during routine assessment of the FAARM trial in Sylhet, Bangladesh

Category	*Indicator*	*Definition*	*Frequency of collection*
Pregnancy & birth identification	Last menstrual period	First day of last menstrual period	Every two months
	Pregnancy status	Identification of new, continued, or loss of pregnancy	Every two months
	Newborn identification	Newborn date of birth record	Every two months
Prenatal and postnatal health	Intended pregnancy	If the woman had intended to get pregnant at this time	Once pregnancy is identified
	ANC attendance	Antenatal care-seeking and home visits by health workers	Every two months during pregnancy
	ANC services	Woman's weight taken, blood pressure measured, urine sample taken, blood sample taken, ultrasonography done, child position checked, eye check for anemia, tetanus toxoid injection given	Every two months during pregnancy
	Iron and folic acid consumption	Intake of iron and folic acid in the previous 7 days	Every two months during pregnancy
	Resting time	Hours of rest and sleep in the previous 24 hours	Every two months during pregnancy
	Night blindness	Difficulty seeing at dusk or night	Every two months during pregnancy
	Pregnancy complications	Reporting of twins, severe headaches, high blood pressure, etc.	Once per pregnancy (≥ 6 months of gestation)
	Past complications	Reporting of past pregnancy complications	Asked once during first reported pregnancy

	Birth delivery plan	Intended place of delivery	Once per pregnancy (≥ 6 months), confirmed in follow-up visits
	Depression	Edinburgh Postnatal Depression Scale (adapted to Bangladesh)[1]	Once during pregnancy (≥ 6 months gestation), once when child is born and once when child is 6-12 months of age
Women's nutrition	Dietary diversity	Information on 21 food groups[2 3]	Every 6 months for all women<br>Every 2 months during pregnancy
	Weight	Weight of women in kg using Tanita scales	Every 12 months for all women<br>Every 2 months during pregnancy
Children's nutrition	IYCF	WHO IYCF indicators[4]	
	Dietary diversity	Information on 21 food groups[2 3]	
	Weight	Weight of children in kg using Tanita scales	When child is approximately 18 months of age
Child morbidity	Diarrhea	7-day period prevalence; 2-day point prevalence	Every two months
	Respiratory distress	7-day period prevalence	Every two months
	Sick child feeding & healthcare seeking	If child was taken to health care facility, child feeding behaviors	Every two months, when child was reported as ill
Child development	Motor Milestones	Child performance of developmental motor milestones according to age[5]	When child reaches appropriate age for milestone
	Language	Child language questionnaire adapted from Frongillo et al, 2017[6]	Every two months when child is 5-11 months of age
Agriculture	Crop diversity	Listing of vegetables, fruits, and spices grown in the past growing season.	

ANC: antenatal care; **IYCF**: Infant and young child feeding;

For peer review only - http://bmjopen.bmj.com/site/about/guidelines.xhtml

References:

1. Gausia K, Hamadani JD, Islam MM, et al. Bangla translation, adaptation and piloting of Edinburgh Postnatal Depression Scale. *Bangladesh Medical Research Council Bulletin* 2007;33(3):81-7. [published Online First: 2008/09/12]
2. Wiesmann D, Arimond M, Loechi C. Dietary diversity as a measure of the micronutrient adequacy of women's diets: results from rural Mozambique site. Washington, DC: Food and Nutrition Technical Assistance II Project, FHI 360, 2009.
3. FAO, FHI 360. Minimum Dietary Diversity for Women: A Guide to Measurement. Rome, Italy: FAO 2016.
4. World Health Organization (WHO). Indicators for assessing infant and young child feeding practices: Part 2 Measurement. Geneva, Switzerland: WHO 2010.
5. WHO Motor Development Study: windows of achievement for six gross motor development milestones. *Acta Paediatrica (Oslo, Norway: 1992) Supplement* 2006;450:86-95. [published Online First: 2006/07/05]
6. Frongillo EA, Nguyen PH, Saha KK, et al. Large-Scale Behavior-Change Initiative for Infant and Young Child Feeding Advanced Language and Motor Development in a Cluster-Randomized Program Evaluation in Bangladesh. *The Journal of Nutrition* 2017;147(2):256-63. doi: 10.3945/jn.116.240861 [published Online First: 2016/12/30]

For peer review only

Table S2: FAARM trial measures of anthropometry, biomarkers, and food contaminants

Category	*Indicator*	*Time of measurement*	*Equipment / Laboratory test*
Anthropometry	Woman weight	Baseline;<br>Routine Assessment (once per year, every 2 months during pregnancy);<br>Endline	Baseline/Endline: SECA 874 digital floor scale<br>RA: Tanita weight scales
	Woman height	Baseline;<br>Endline[a]	SECA 217 stadiometer
	Woman MUAC	Routine Assessment<br>(every 2 months during pregnancy)	SECA 212 measuring tape
	Child weight	Baseline;<br>Routine Assessment<br>(around 18 months);<br>Endline	Baseline/Endline: SECA 874 digital floor scale<br>RA: SECA 334
	Child length	Baseline;<br>Birth Registration (newborn length);<br>Endline	Baseline/Endline: SECA 416 digital baby scale<br>BR: SECA 232 measuring rod (until March 2017); locally made length board (from April 2017)
	Child MUAC	Birth Registration	SECA 212
	Child head circumference	Birth Registration	SECA 212
Biomarkers	Hemoglobin	Baseline;<br>Endline	Hemocue 201+<br>Sysmex XP-100 Hematology Analyzer
	Serum ferritin[b]	Baseline;<br>Endline	Sandwich ELISA[1]
	Serum transferrin receptor[b]	Baseline;<br>Endline	Sandwich ELISA[1]

For peer review only

	Retinol-binding protein[b]	Baseline; Endline	Sandwich ELISA[1]
	C-reactive protein[b]	Baseline; Endline	Sandwich ELISA[1]
	Serum α-1-acid glycoprotein[b]	Baseline; Endline	Sandwich ELISA[1]
	Serum zinc[b]	Endline	Colorimetric method
	Feacal myeloperoxidase[b]	2018 (Food hygiene evaluation); Endline	Sandwich ELISA[1]
	Feacal α-1-antitrypsin[b]	2018 (Food hygiene evaluation); Endline	Sandwich ELISA[1]
	Feacal neopterin[b]	2018 (Food hygiene evaluation); Endline	Sandwich ELISA[1]
Food contaminants	Coliforms[b]	2018 (Food hygiene evaluation)	Pour plating on Tryptone Bile X-Glucuronide (TBX) Agar
	Escherichia coli[b]	2018 (Food hygiene evaluation)	Pour plating on Tryptone Bile X-Glucuronide (TBX) Agar

[a] For those women who were not measured at baseline. [b] These measurements will be conducted in a sub-sample of the population.
MUAC: mid-upper arm circumference

1. Erhardt JG, Estes JE, Pfeiffer CM, et al. Combined measurement of ferritin, soluble transferrin receptor, retinol binding protein, and C-reactive protein by an inexpensive, sensitive, and simple sandwich enzyme-linked immunosorbent assay technique. *The Journal of Nutrition* 2004;134(11):3127-32. doi: 10.1093/jn/134.11.3127

For peer review only

STANDARD PROTOCOL ITEMS: RECOMMENDATIONS FOR INTERVENTIONAL TRIALS

SPIRIT 2013 Checklist: Recommended items to address in a clinical trial protocol and related documents*

Section/item	Item No	Description	Addressed on page number
**Administrative information**			
Title	1	Descriptive title identifying the study design, population, interventions, and, if applicable, trial acronym	__1___________
Trial registration	2a	Trial identifier and registry name. If not yet registered, name of intended registry	___1__________
	2b	All items from the World Health Organization Trial Registration Data Set	_Throughout____
Protocol version	3	Date and version identifier	____n/a________
Funding	4	Sources and types of financial, material, and other support	____23_________
Roles and responsibilities	5a	Names, affiliations, and roles of protocol contributors	____1, 23_______
	5b	Name and contact information for the trial sponsor	___n/a______
	5c	Role of study sponsor and funders, if any, in study design; collection, management, analysis, and interpretation of data; writing of the report; and the decision to submit the report for publication, including whether they will have ultimate authority over any of these activities	____23________
	5d	Composition, roles, and responsibilities of the coordinating centre, steering committee, endpoint adjudication committee, data management team, and other individuals or groups overseeing the trial, if applicable (see Item 21a for data monitoring committee)	____n/a________

For peer review only - http://bmjopen.bmj.com/site/about/guidelines.xhtml

**Introduction**			
Background and rationale	6a	Description of research question and justification for undertaking the trial, including summary of relevant studies (published and unpublished) examining benefits and harms for each intervention	_____4-5_____
	6b	Explanation for choice of comparators	___5, 9_______
Objectives	7	Specific objectives or hypotheses	___5_________
Trial design	8	Description of trial design including type of trial (eg, parallel group, crossover, factorial, single group), allocation ratio, and framework (eg, superiority, equivalence, noninferiority, exploratory)	__6__________
**Methods: Participants, interventions, and outcomes**			
Study setting	9	Description of study settings (eg, community clinic, academic hospital) and list of countries where data will be collected. Reference to where list of study sites can be obtained	__6__________
Eligibility criteria	10	Inclusion and exclusion criteria for participants. If applicable, eligibility criteria for study centres and individuals who will perform the interventions (eg, surgeons, psychotherapists)	___7-8_______
Interventions	11a	Interventions for each group with sufficient detail to allow replication, including how and when they will be administered	___9-12______
	11b	Criteria for discontinuing or modifying allocated interventions for a given trial participant (eg, drug dose change in response to harms, participant request, or improving/worsening disease)	___n/a_______
	11c	Strategies to improve adherence to intervention protocols, and any procedures for monitoring adherence (eg, drug tablet return, laboratory tests)	___13-14_____
	11d	Relevant concomitant care and interventions that are permitted or prohibited during the trial	____n/a______
Outcomes	12	Primary, secondary, and other outcomes, including the specific measurement variable (eg, systolic blood pressure), analysis metric (eg, change from baseline, final value, time to event), method of aggregation (eg, median, proportion), and time point for each outcome. Explanation of the clinical relevance of chosen efficacy and harm outcomes is strongly recommended	___16-18_____
Participant timeline	13	Time schedule of enrolment, interventions (including any run-ins and washouts), assessments, and visits for participants. A schematic diagram is highly recommended (see Figure)	_Figure 3_____

For peer review only - http://bmjopen.bmj.com/site/about/guidelines.xhtml

Sample size	14	Estimated number of participants needed to achieve study objectives and how it was determined, including clinical and statistical assumptions supporting any sample size calculations	__7___________
Recruitment	15	Strategies for achieving adequate participant enrolment to reach target sample size	_7-8_________

Methods: Assignment of interventions (for controlled trials)

Allocation:

Sequence generation	16a	Method of generating the allocation sequence (eg, computer-generated random numbers), and list of any factors for stratification. To reduce predictability of a random sequence, details of any planned restriction (eg, blocking) should be provided in a separate document that is unavailable to those who enrol participants or assign interventions	__8-9________
Allocation concealment mechanism	16b	Mechanism of implementing the allocation sequence (eg, central telephone; sequentially numbered, opaque, sealed envelopes), describing any steps to conceal the sequence until interventions are assigned	__9________
Implementation	16c	Who will generate the allocation sequence, who will enrol participants, and who will assign participants to interventions	_7-9_________
Blinding (masking)	17a	Who will be blinded after assignment to interventions (eg, trial participants, care providers, outcome assessors, data analysts), and how	_n/a_________
	17b	If blinded, circumstances under which unblinding is permissible, and procedure for revealing a participant's allocated intervention during the trial	_n/a_________

Methods: Data collection, management, and analysis

Data collection methods	18a	Plans for assessment and collection of outcome, baseline, and other trial data, including any related processes to promote data quality (eg, duplicate measurements, training of assessors) and a description of study instruments (eg, questionnaires, laboratory tests) along with their reliability and validity, if known. Reference to where data collection forms can be found, if not in the protocol	__13-18______
	18b	Plans to promote participant retention and complete follow-up, including list of any outcome data to be collected for participants who discontinue or deviate from intervention protocols	__19_____

Data management	19	Plans for data entry, coding, security, and storage, including any related processes to promote data quality (eg, double data entry; range checks for data values). Reference to where details of data management procedures can be found, if not in the protocol	__18-19______
Statistical methods	20a	Statistical methods for analysing primary and secondary outcomes. Reference to where other details of the statistical analysis plan can be found, if not in the protocol	__19______
	20b	Methods for any additional analyses (eg, subgroup and adjusted analyses)	__19______
	20c	Definition of analysis population relating to protocol non-adherence (eg, as randomised analysis), and any statistical methods to handle missing data (eg, multiple imputation)	__19______
**Methods: Monitoring**			
Data monitoring	21a	Composition of data monitoring committee (DMC); summary of its role and reporting structure; statement of whether it is independent from the sponsor and competing interests; and reference to where further details about its charter can be found, if not in the protocol. Alternatively, an explanation of why a DMC is not needed. *The FAARM trial is a behavioural trial and has only minimal risk. Therefore, a data monitoring committee was not required (Lin et al, 2014).*	________see explanation___
	21b	Description of any interim analyses and stopping guidelines, including who will have access to these interim results and make the final decision to terminate the trial	_n/a_________
Harms	22	Plans for collecting, assessing, reporting, and managing solicited and spontaneously reported adverse events and other unintended effects of trial interventions or trial conduct	__13-18, 20___
Auditing	23	Frequency and procedures for auditing trial conduct, if any, and whether the process will be independent from investigators and the sponsor	___n/a______
**Ethics and dissemination**			
Research ethics approval	24	Plans for seeking research ethics committee/institutional review board (REC/IRB) approval	_____19-20____
Protocol amendments	25	Plans for communicating important protocol modifications (eg, changes to eligibility criteria, outcomes, analyses) to relevant parties (eg, investigators, REC/IRBs, trial participants, trial registries, journals, regulators)	_____20______

Consent or assent	26a	Who will obtain informed consent or assent from potential trial participants or authorised surrogates, and how (see Item 32)	_____8, 20______
	26b	Additional consent provisions for collection and use of participant data and biological specimens in ancillary studies, if applicable	____8, 20_____
Confidentiality	27	How personal information about potential and enrolled participants will be collected, shared, and maintained in order to protect confidentiality before, during, and after the trial	____19_______
Declaration of interests	28	Financial and other competing interests for principal investigators for the overall trial and each study site	____23_______
Access to data	29	Statement of who will have access to the final trial dataset, and disclosure of contractual agreements that limit such access for investigators	___23________
Ancillary and post-trial care	30	Provisions, if any, for ancillary and post-trial care, and for compensation to those who suffer harm from trial participation	__n/a________
Dissemination policy	31a	Plans for investigators and sponsor to communicate trial results to participants, healthcare professionals, the public, and other relevant groups (eg, via publication, reporting in results databases, or other data sharing arrangements), including any publication restrictions	___3, 22_____
	31b	Authorship eligibility guidelines and any intended use of professional writers	_followed Vancouver criteria____
	31c	Plans, if any, for granting public access to the full protocol, participant-level dataset, and statistical code	__n/a_________
**Appendices**			
Informed consent materials	32	Model consent form and other related documentation given to participants and authorised surrogates	___n/a_______
Biological specimens	33	Plans for collection, laboratory evaluation, and storage of biological specimens for genetic or molecular analysis in the current trial and for future use in ancillary studies, if applicable	Table S2; Supp File 2

For peer review only - http://bmjopen.bmj.com/site/about/guidelines.xhtml

*It is strongly recommended that this checklist be read in conjunction with the SPIRIT 2013 Explanation & Elaboration for important clarification on the items. Amendments to the protocol should be tracked and dated. The SPIRIT checklist is copyrighted by the SPIRIT Group under the Creative Commons "Attribution-NonCommercial-NoDerivs 3.0 Unported" license.

For peer review only
