# Supplementary material for: Food and Agricultural Approaches to Reducing Malnutrition (FAARM): protocol for a cluster-randomised controlled trial to evaluate the impact of a Homestead Food Production programme on undernutrition in rural Bangladesh
Source: BMJ Open. 2019 Jul 4;9(7):e031037. doi: 10.1136/bmjopen-2019-031037 (PMC6615849; doi:10.1136/bmjopen-2019-031037)
Supplement: Reviewer comments [file bmjopen-2019-031037.reviewer_comments.pdf]

PEER REVIEW HISTORY

BMJ Open publishes all reviews undertaken for accepted manuscripts. Reviewers are asked to complete a checklist review form (**http://bmjopen.bmj.com/site/about/resources/checklist.pdf**) and are provided with free text boxes to elaborate on their assessment. These free text comments are reproduced below.

ARTICLE DETAILS

**TITLE (PROVISIONAL)**	Food and Agricultural Approaches to Reducing Malnutrition (FAARM): Protocol for a cluster-randomised controlled trial to evaluate the impact of a Homestead Food Production programme on undernutrition in rural Bangladesh
**AUTHORS**	Wendt, Amanda; Sparling, Thalia M; Waid, Jillian; Mueller, Anna; Gabrysch, Sabine

This article was not externally reviewed at BMJ Open. Protocols that have been independently assessed prior to submission to BMJ Open are usually fast-tracked to publication on the grounds that further substantial changes will not be possible. This independent assessment will usually be external, independent review for both a substantial grant award from a non-commercial or government funder and ethics approval.
